# Supplementary material for: Complexity of avian evolution revealed by family-level genomes
Source: Nature. 2024 Apr 1;629(8013):851–60. doi: 10.1038/s41586-024-07323-1 (PMC11111414; doi:10.1038/s41586-024-07323-1)
Supplement: Supplementary file 1 — This file contains Supplementary methods and results. [file 41586_2024_7323_MOESM1_ESM.docx]

**Supplementary Information**

**Fossil calibrations, Supplementary Methods, Supplementary Results**

**Complexity of avian evolution revealed by family-level genomes**

Josefin Stiller, Shaohong Feng, Al-Aabid Chowdhury, Iker Rivas-González, David A. Duchêne, Qi Fang, Yuan Deng, Alexey Kozlov, Alexandros Stamatakis, Santiago Claramunt, Jacqueline M. T. Nguyen, Simon Y. W. Ho, Brant C. Faircloth, Julia Haag, Peter Houde, Joel Cracraft, Metin Balaban, Uyen Mai, Guangji Chen, Rongsheng Gao, Chengran Zhou, Yulong Xie, Zijian Huang, Zhen Cao, Zhi Yan, Huw A. Ogilvie, Luay Nakhleh, Bent Lindow, Benoit Morel, Jon Fjeldså, Peter A. Hosner, Rute R. da Fonseca, Bent Petersen, Joseph A. Tobias, Tamás Székely, Jonathan David Kennedy, Andrew Hart Reeve, Andras Liker, Martin Stervander, Agostinho Antunes, Dieter Thomas Tietze, Mads Bertelsen, Fumin Lei, Carsten Rahbek, Gary R. Graves, Mikkel H. Schierup, Tandy Warnow, Edward L. Braun, M. Thomas P. Gilbert, Erich D. Jarvis, Siavash Mirarab, Guojie Zhang

Correspondence to: Josefin Stiller (josefin.stiller@bio.ku.dk), Siavash Mirarab (smirarabbaygi@ucsd.edu), Guojie Zhang ([guojiezhang@zju.edu.cn](mailto:guojiezhang@zju.edu.cn))

**Table of Contents**

[**Fossil Calibrations 3**](#_5raepkulw44v)

[PALAEOGNATHAE 4](#_kghokelczxiy)

[NEOGNATHAE 12](#_m6b8hvtq6t32)

[GALLOANSERES 12](#_1ze6zrxte35t)

[NEOAVES 17](#_78ipydbjvkks)

[PHOENICOPTERIMORPHAE 17](#_udoo5w1t56sj)

[OTIDIMORPHAE 19](#_z51r2ubmobr)

[STRISORES 21](#_7ekianqqcew6)

[CURSORIMORPHAE 25](#_li48bfje5ikx)

[AEQUORNITHES 33](#_vrmr9a4v9oqw)

[TELLURAVES 49](#_b88k4x6t95k6)

[**Supplementary Methods 79**](#_9egkmkjnej46)

[Extraction of intergenic regions and filtering 79](#_fm1zh2bvikk1)

[Extraction of introns and filtering 81](#_epz9hnhp7ynm)

[Alignment and filtering of protein-coding regions 81](#_4z894t3iqsrf)

[Extraction, alignment, and filtering of Ultraconserved Elements (UCEs) 82](#_ant29necsycj)

[Inference of gene trees and summary into species trees 82](#_mst9w9zi6ylv)

[Concatenation-based species tree 83](#_260oijfnxma8)

[Fossil calibrations and derivation of calibration densities 84](#_oeeg4jjh1c7n)

[Molecular dating of the species tree 85](#_v86jshcr5zkx)

[Subsetting analyses 87](#_1zt9mu9jkgnd)

[By taxon sampling 87](#_h8iviqsvpzg8)

[By data quantity 88](#_1cfgdha8uyte)

[By data type 89](#_nesm65njbj8k)

[By genomic characteristics 89](#_hjco416tp1qp)

[By chromosome and chromosomal category 90](#_i8cglssvmtjy)

[​​Phylogenetic model adequacy 91](#_aofxt0a0sbb8)

[CoalHMM analyses 91](#_pgmb7stuhjfa)

[GC content differences within Palaeognathae 92](#_sxkmxrgk9ywo)

[Inference of effective population size 93](#_bb75gi99j75p)

[Analysis of molecular evolutionary rates 94](#_479ihq8elf9y)

[Analysis of phylogenetic signal 94](#_wz0qs9hl5fhg)

[Analysis of body mass and brain size evolution 95](#_9se7b7t3kyfc)

[**Supplementary Results 97**](#_b3acgru0fb0o)

[Relationships within Passeriformes 97](#_4nbymzh4p75q)

[Impact of taxon sampling on phylogenetic signal 97](#_smol62vge8so)

[**References 98**](#_g7u5gl9tixvn)

#

# Fossil Calibrations

**Format follows the following template:**

**Calibration: (clade name or indication)**

**Category: A - primary calibration based on top-quality fossils; B - secondary calibration**

**MRCA of:** {taxon1 and taxon2} (a definition of the clade based on descendant taxa)

**Clade definition in tree:** {sp1, sp2} (using species actually included in the B10k tree whose MRCA corresponds to the node to be calibrated)

**Oldest fossil:** (Species, material, and geological and geographical provenance of the oldest fossil that can be confidently considered part of the crown clade.)

**Phylogenetic placement justification:** (Evidence for phylogenetic placement, ideally a cladistic analysis but putative derived states can also be used as evidence in some cases)

**Minimum age:** (the minimum possible age of the oldest fossil).

**Minimum age justification:** Evidence for the time interval allocation or the point estimate of the age of the oldest fossil, in particular regarding the minimum age stated above.

**First occurrences:** (table with the list of fossils that represent the first occurrence of the clade in each major landmass (for widespread clades) or first occurrences of fossils species from different formations (for clades restricted to a single landmass).

**Fossil record remarks:** (comments on fossils used and other details)

**Clade age estimation:** (summary of the output from the function clade.date (CladeDate R package, Claramunt 2022), including the result of a Kolmogorov-Smirnov test of uniformity of the fossil ages, quantiles of the distribution generated by clade.date, and the parameters of a fitted skew-Student function.

**References:** (references cited in the previous text)

## PALAEOGNATHAE

**Calibration: crown Casuariidae**

**Category: A**

**MRCA of:** *Casuarius* and *Dromaius*

**Clade definition in tree:** *Casuarius casuarius*, *Dromaius novaehollandiae*

**Oldest fossil:** *Emuarius gidju* (Patterson & Rich, 1987). The oldest material of this taxon (a tibiotarsus QM F16827 and a tarsometatarsus QM F56127) comes from White Hunter Site, Riversleigh World Heritage Area, Queensland, Australia.

**Phylogenetic placement justification:** A cladistic analysis of *Emuarius gidju* (coded from several specimens including the holotype), other 24 paleognaths, three galloansereans and 179 morphological characters recovered *Emuarius gidju* as sister to *Dromaius* with 76% bootstrap support (Worthy *et al.* 2014). Therefore, *Emuarius gidju* sets a minimum age for crown Casuariidae.

**Minimum age:**  23.04 Ma.

**Minimum age justification:** White Hunter Site is allocated to Riversleigh Faunal Zone A (late Oligocene) based on biocorrelation of vertebrate faunas with those from the Etadunna Formation (e.g., Archer et al. 1989; Travouillon et al. 2006). This formation was estimated to be 26–24 Ma based on magnetostratigraphy and biocorrelation of land mammals and forams (Woodburne et al. 1994). We use the upper boundary of the late Oligocene (Chattian) (23.04 Ma) as a minimum age for the fossil.

**Table of first fossil occurrences:**

- *Emuarius gidju* (Patterson & Rich, 1987) from White Hunter Site (late Oligocene, 26–23.04 Ma), Riversleigh, Queensland, Australia (Boles 1992, Worthy *et al.* 2014)
- *Dromaius arleyekweke* Yates & Worthy, 2019 from the Waite Formation (Miocene, 12–5 Ma) at Main Pit, Alcoota Scientific Reserve, Northern Territory, Australia
- *Dromaius novaehollandiae* (Latham, 1790) from Chinchilla (Pliocene, 5.3–2.6 Ma), Darling Downs, Queensland, Australia (Louys & Price 2013)
- *Dromaius ocypus* Miller, 1963 from the Lawson-Daily Quarry (Pliocene, 5.3–2.6 Ma), Lake Palankarinna, South Australia, Australia (Petterson & Rich 1987)
- *Casuarius* cf. *bennetti* Gould, 1857 from Awe (Pliocene, 3.3–2.5 Ma, Hoch & Holm 1986), Watut River, Papua New Guinea (Plane 1967)

**Fossil record remarks:** Because the clade is endemic to the Australian continent, we used the first occurrence of all species. A late Oligocene fossil described as *Emuarius guljaruba* Boles, 2001 from the Ngama Local Fauna is here considered to be synonymous with *E. gidju* (Worthy *et al.* 2014). *Casuarius lydekkeri* Rothschild, 1911 is not considered here because its provenance and age are unknown (Worthy & Nguyen 2020).

**Clade age estimation:** Strauss-Sadler, Kolmogorov-Smirnov test of uniformity:

D = 0.4926, p-value = 0.1221

**Quantiles**:

0% 50% 95% 97.5%

23.10 27.82 42.61 48.91

**Parameters of the skewStudent function:**

offset xi omega alpha nu

23.104 24.055 5.002 9.522 2.492

**
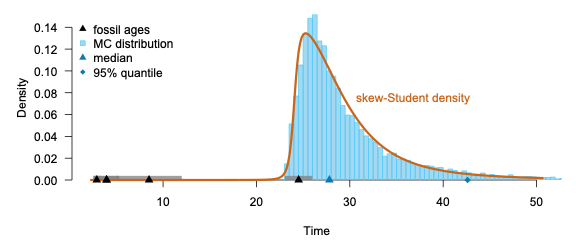
**

**References:**

Archer, M., Godthelp, H., Hand, S. J., & Megirian, D. 1989. Fossil mammals of Riversleigh, northwestern Queensland: Preliminary overview of biostratigraphy, correlation and environmental change. *Australian Zoologist*, *25*, 29–65.

Boles, W. E. 1992. Revision of *Dromaius gidju* Patterson and Rich 1987 from Riversleigh, Northwestern Queensland, Australia, with a reassessment of its generic position. In K. E. Campbell Jr. (ed.) ‘Papers in Avian Paleontology Honoring Pierce Brodkorb’. *Natural History Museum of Los Angeles County, Science Series* **36**:195–208.

Boles, W. R. 2001. A new emu (Dromaiinae) from the Late Oligocene Etadunna Formation. Emu 101(4):317-321.

Hoch E. & P. M. Holm. 1986. New K/Ar age determinations of the Awe fauna gangue, Papua New Guinea: consequences for Papuaustralian late Cenozoic biostratigraphy. Modern Geology 10, 181-195.

Louys, J. & Price, G.J. 2015. The Chinchilla Local Fauna: An exceptionally rich and well-preserved Pliocene vertebrate assemblage from fluviatile deposits of south-eastern Queensland, Australia. Acta Palaeontologica Polonica 60(3): 551–572.

Patterson, C., & P. V. Rich 1987. The fossil history of the emus, *Dromaius* (Aves: Dromaiinae). Records of The South Australian Museum 21:85-117.

Plane, M.D. 1967. Stratigraphy and vertebrate fauna of the Otibanda Formation, New Guinea. Bureau of Mineral Resources, Geology and Geophysics (Australia) Bulletin 86:1-64.

Travouillon, K. J., M Archer, S. J. Hand & H. Godthelp. 2006 Multivariate analyses of Cenozoic mammalian faunas from Riversleigh, northwestern Queensland. Alcheringa 30(S1):323-349.

Woodburne, M. O., MacFadden, B. J., Case, J. A., Springer, M. S., Pledge, N. S., Power, J. D., Woodburne, J. M., & Springer, K. B. (1994). Land mammal biostratigraphy and magnetostratigraphy of the Etadunna Formation (Late Oligocene) of South Australia. Journal of Vertebrate Paleontology, 13(4), 483–515.

Worthy, T. H., S. J. Hand, M. Archer. 2014. Phylogenetic relationships of the Australian Oligo-Miocene ratite *Emuarius gidju* Casuariidae. Integrative Zoology 9: 148–166.

Worthy, T. H. & J. M. T. Nguyen 2020. An annotated checklist of the fossil birds of Australia. Transactions of the Royal Society of South Australia 144 (1):66-108.

Yates, A. M., & T. H. Worthy. 2019. A diminutive species of emu (Casuariidae: Dromaiinae) from the late Miocene of the Northern Territory, Australia. Journal of Vertebrate Paleontology 39(4): DOI: 10.1080/02724634.2019.1665057.

**Calibration: crown Rheidae**

**Category**: B

**MRCA of:** *Rhea americana* and *R. pennata*

**Clade definition in tree:** *Rhea americana*, *Rhea pennata*

**Oldest fossil:** *Opisthodactylus horacioperezi* Agnolin & Chafrat, 2015, represented by a distal end of a left tibiotarsus (holotype MPCN-PV-380), an incomplete lateral condyle of a right tibiotarsus (MPCN-PV-378) and an incomplete distal end of a right tarsometatarsus (MPCN-PV-376) from the Chichinales Formation at Paso Córdoba, Río Negro province, Argentina.

**Phylogenetic placement justification:** A cladistic analysis found a monophyletic *Opisthodactylus*, including *O. horacioperezi*, *O. patagonicus*, and *O. kirchenri* as sister to (*Rhea pennata* + *R. mesopotamica*), with *Rhea americana* in a more basal position (Noriega *et al.* 2017). The analysis included only 21 characters of the hindlimb bones, and the outgroup was the tinamou *Eudromia elegans*.

**Minimum age:** 20.1 Ma

**Minimum age justification:** The Chichinales Formation is correlated to the Colhuehuapian (early Miocene) South American Land Mammal Age on the basis of its mammalian fauna (Agnolin & Chafrat 2015). Dunn *et al.* (2013) refined the age of the Colhuehuapian to 21.1–20.1 Ma based on U-Pb dating.

**First occurrences:**

- *Opisthodactylus horacioperezi* Agnolin & Chafrat, 2015 from the Chichinales Formation (early Miocene, Colhuehuapian, 21.1–20.1 Ma) at Paso Córdoba, Rio Negro province, Argentina.
- *Opisthodactylus patagonicus* Ameghino, 1891 from the Santa Cruz Formation at Cerro Observatorio (Miocene, 17.8 – 16.3 Ma, Perkins *et al*. 2012) , Argentina (Picasso *et al*. 2022).
- *Opisthodactylus kirchneri* Noriega *et al.*, 2017 from the lower levels of the Andalhuala Formation (late Miocene, Huayquerian, 8.7–6.8 Ma), Tucumán, Argentina.
- *Pterocnemia mesopotamica* Agnolin & Noriega, 2012 from the Ituzaingó Formation (late Miocene, Huayquerian, 8.7–6.8 Ma) at Entre Rios, Argentina.
- *Protorhea azarae* Moreno & Mercerat, 1891 from Farola Monte Hermoso (early Pliocene, Montehermosan, 5–4.5 Ma), Argentina. (Also from the same site: *Heterorhea dabbenei* Rovereto, 1914 and *Hinasuri nehuensis* Tambussi, 1995)
- *Rhea americana*, a distal tarsometatarsus from Estación Anchorena, Martinez Locality (Pleistocene, Ensenadan, 1.8–0.781 Ma), Buenos Aires, Argentina (Picasso & Mosto, 2016).
- *Pterocnemia pennata* from Paso Otero (Late Pleistocene, Lujanian, 0.781–0.012 Ma), Buenos Aires, Argentina (Tonni & Laza 1980)

**Fossil record remarks**: Given the exclusively South American distribution of Rheidae, the oldest record of each Rheidae species was used to estimate age uncertainty.

**Clade age estimation:**

One-sample Kolmogorov-Smirnov test

data: Mages

D = 0.37568, p-value = 0.2141

alternative hypothesis: two-sided

Quantiles:

0% 50% 95% 97.5%

20.12 22.74 31.69 34.78

Parameters of the skewStudent function:

offset xi omega alpha nu

20.116 20.439 3.077 18.423 2.580

**
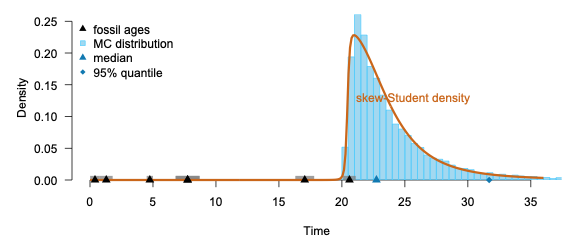
**

**References:**

Agnolin, F., & P. Chafrat. 2015. New fossil bird remains from the Chichinales Formation (Early Miocene) of northern Patagonia, Argentina. Annales de Páleontologie 101:87–94.

Agnolin, F., & J. I. Noriega. 2012. Una nueva especie de nandú (Aves: Rheidae) del Mioceno tardío de la Mesopotamia argentina. Ameghiniana 49:236–246.

Ameghino, F. 1891. Enumeración de las aves fósiles de la República Argentina. Revista Argentina de Historia Natural 1:441-453

Dunn, R.E., Madden, R.H., Kohn, M.J., Schmitz, M.D., Strömberg, C.A., Carlini, A.A., Ré, G.H. and Crowley, J., 2013. A new chronology for middle Eocene–early Miocene South American land mammal ages. Geological Society of America Bulletin 125(3-4):539-555.

Moreno, F. P. & A. Mercerat. 1891. Catalogue des Oiseaux Fossiles de la Republique Argentine conserves au Musee de la Plata. Anales del Museo de la Plata 1:1-71

Noriega, J. I., E. A. Jordan, R. I. Vezzosi & J. I. Areta. 2017. A new species of *Opisthodactylus* Ameghino, 1891 (Aves, Rheidae), from the late Miocene of northwestern Argentina, with implications for the paleobiogeography and phylogeny of rheas. Journal of Vertebrate Paleontology 37(1): e1278005.

Perkins, M.E., Fleagle, J.G., Heizler, M.T., Nash, B., Bown, T.M., Tauber, A.A., Dozo, M.T., 2012. Tephrochronology of the Miocene Santa Cruz and Pinturas formations, Argentina. In: Vizcaíno, S.F., Kay, R.F., Bargo, M.S. (Eds.), Early Miocene Paleobiology in Patagonia: High-latitude Paleocommunities of the Santa Cruz Formation. Cambridge University Press.

Picasso, M. B. J. & C. Mosto. 2016 The new taxonomic status of *Rhea anchorenensis* (Ameghino and Rusconi, 1932) (Aves, Palaeognathae) from the Pleistocene of Argentina. Annales de Paléontologie 102(4):237-231

Picasso, M. B. J., Hospitaleche, C. A. & C. Mosto. 2022. An overview and update of South American and Antarctic fossil rheidae and putative ratitae (Aves, Palaeognathae). Journal of South American Earth Sciences 115:103731.

Tonni, E. P, & J. H. Laza 1980 Las aves de la fauna local Paso Otero (Pleistoceno tardío) de la provincia de Buenos Aires, su significación ecológica, climática y zoogeográfica. Ameghiniana 42:313-322.

**Calibration: crown Nothurinae**

**Category: A**

**MRCA of:** *Eudromia* and *Nothoprocta*

**Clade definition in tree:** *Eudromia elegans*, *Nothoprocta perdicaria*

**Oldest fossil:** “*Eudromia”* sp., nearly complete right coracoid (MLP 87-XI-20-3) from the Cerro Azul Formation at Salinas Grandes de Hidalgo (formerly Epecuén Formation), La Pampa Province, Argentina (Tambussi 1987; Cenizo *et al.* 2012).

**Phylogenetic placement justification:** Cladistic analysis (Bertelli *et al.* 2014; Bertelli 2017) placed this fossil as sister to the *Eudromia*-*Tinamotis* clade thus immediately above the most recent common ancestor of Nothurinae.

**Minimum age:** 6.8 Ma

**Minimum age justification:** The Cerro Azul Formation at Salinas Grandes is estimated to span between 6 and 7.2 Ma ago, as determined by mammal biostratigraphy (Rasia *et al.* 2020).

**First occurrences:**

- *“Eudromia”* sp. from the Cerro Azul Formation at Salinas Grandes de Hidalgo (late Miocene, Huayquerian, 7.2–6 Ma), La Pampa province, Argentina (Tambussi 1987)
- *Eudromia olsoni* Tambussi & Tonni, 1985, humerus fragments, distal femur, incomplete pelvis and tibiotarsus from Farola Monte Hermoso (early Pliocene, Montehermosan, 5–4.5 Ma), Argentina.
- *Nothura parvula* Tambussi, 1989, distal tibiotarsus, tarsometatarsus, proximal femur and phalanges from the Chapadmalal Formation (early Pliocene, Montehermosan, 5–4.5 Ma), Buenos Aires Province, Argentina.
- *Nothura darwinii* Gray, 1867 from Bajo San José Lower Section (Pleistocene, 0.781–0.126 Ma), Buenos Aires province, Argentina.
- *Nothura paludosa* Mercerat, 1897 a femur from the Buenos Aires Formation at Arrecifes (Pleistocene, 0.126–0.012 Ma), Buenos Aires province, Argentina.
- *Nothoprocta ornata*, unspecified material from the Early Formative period (Holocene, 0.0035–0.0028 Ma, radiocarbon calibrated age) at Chiripa archeological site, Titicaca Lake, Bolivia (Steadman & Hastorf 2015)

**Fossil record remarks:** Because the clade is exclusively South American, the species-level South American record is used. Although the oldest species has not been formally named, cladistic analysis (Bertelli *et al.* 2014, Bertelli 2017) indicates that it is not any of the extant *Eudromia* species and it does not even belong in the genus *Eudromia*. Other fossils not identified at the species level are not included in the record.

**Clade age estimation:**

One-sample Kolmogorov-Smirnov test

data: Mages

D = 0.457, p-value = 0.1149

alternative hypothesis: two-sided

Quantiles:

0% 50% 95% 97.5%

6.016 7.459 10.870 12.140

Parameters of the skewStudent function:

offset xi omega alpha nu

6.016 6.371 1.468 7.309 3.383

**
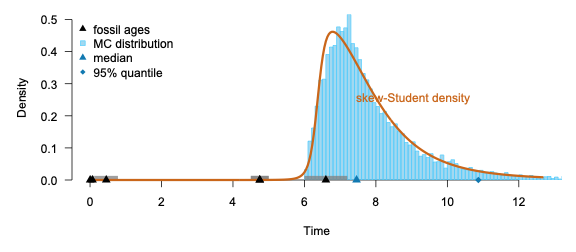
**

**References:**

Bertelli, S., 2017. Advances on tinamou phylogeny: an assembled cladistic study of the volant palaeognathous birds. Cladistics 33(4):351-374.

Bertelli, S., L. M. Chiappe & G. Mayr 2014. Phylogenetic interrelationships of living and extinct Tinamidae, volant palaeognathous birds from the New World. Zoological Journal of the Linnean Society 172(1):145-184.

Cenizo, M. M., C. P. Tambussi & C. I. Montalvo. 2012. Late Miocene continental birds from the Cerro Azul Formation in the Pampean region (central-southern Argentina). Alcheringa 36(1):47-68.

Mercerat, A. 1897. Note sur las oiseaux fossiles de la republic Argentine. *Anales de la Sociedad Cientifica Argentina* 43:222-240

Tambussi C. 1987. Catálogo crítico de los Tinamidae (Aves: Tinamiformes) fósiles de la República Argentina. Ameghiniana 24: 241–244.

Tambussi C. 1989. Las aves del Plioceno-tardío Pleistoceno-temprano de la Provincia de Buenos Aires. Unpublished dissertation, Universidad Nacional de La Plata, Argentina.

Tambussi C & Tonni EP. 1985. Un Tinamidae (Aves: Tinamiformes) del Mioceno tardío de La Pampa (República Argentina) y comentarios sobre los tinámidos fósiles argentinos. Revista de la Asociación Paleontológica Argentina 14: 4.

Rasia, L. L., R. A. Bonini & A. M. Candela. 2020. Nuevos registros de *Lagostomus* Brookes, 1828 (Rodentia, Chinchillidae) en el Mioceno tardío de Argentina y su importancia bioestratigráfica. Andean Geology 47(2).

Steadman, D. W. & C. A. Hastorf 2015. Prehistoric birds from the Lake Titicaca region, Bolivia: long-term continuity and change in an Andean bird community. The Wilson Journal of Ornithology 127(3):359-375.

**Calibration: crown Tinaminae-part**

**Category: B**

**MRCA of: *Crypturellus* and *Tinamus***

**Clade definition in tree:** *Crypturellus soui*, *Tinamus guttatus*

**Oldest fossil:** *Crypturellus reai* Chandler, 2012 a humerus (holotype AMNH:FAM 9151) from the Santa Cruz Formation at Cañadón de las Vacas, Santa Cruz province, Argentina.

**Phylogenetic placement justification:** Cladistic analyses placed the fossils in *Crypturellus* although with ambiguity regarding a stem or crown placement (Bertelli *et al.* 2014, Bertelli 2017).

**Minimum age:** 16 Ma

**Minimum age justification:** Tephrochronological correlations and radiometric estimates indicate an age range of 18–16 Ma for the coastal sections of the Santa Cruz Formation (Fleagle *et al.* 2012).

**First occurrences:**

- *Crypturellus reai* Chandler, 2012 from the Santa Cruz Formation at Cañadón de las Vacas (early Miocene, 18–16 Ma), Santa Cruz province, Argentina.
- *Crypturellus noctivagus* and *Crypturellus parvirostris*, unspecified material from Barra do Antonião (late Pleistocene, 0.042–0.0117 Ma), Piauí, Brazil (Guérin *et al.* 1996).

**Fossil record remarks:**

**Clade age estimation:**

Quantiles:

0% 50% 95% 97.5%

16.11 24.10 72.78 108.20

Parameters of the skewStudent function:

offset xi omega alpha nu

16.114 16.579 8.554 33.560 1.360


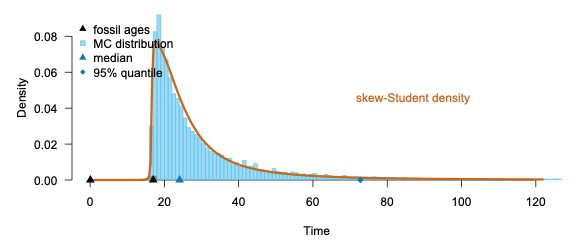


**References:**

Bertelli, S., 2017. Advances on tinamou phylogeny: an assembled cladistic study of the volant palaeognathous birds. Cladistics 33(4):351-374.

Bertelli, S., L. M. Chiappe & G. Mayr 2014. Phylogenetic interrelationships of living and extinct Tinamidae, volant palaeognathous birds from the New World. Zoological Journal of the Linnean Society 172(1):145-184.

Chandler, R. M. 2012. A new species of tinamou (Aves: Tinamiformes, Tinamidae) from the Early-Middle Miocene of Argentina. Palarch’s Journal of Vertebrate Palaeontology 9(2):1-8.

Fleagle, J.G., Perkins, M.E., Heizler, M.T., Nash, B., Bown, T.M., Tauber, A.A., Dozo, M.T. & M. F. Tejedor 2012. Tephrochronology of the Miocene Santa Cruz and Pinturas formations, Argentina. In: Vizcaíno, S.F., Kay, R.F., Bargo, M.S. (Eds.), Early Miocene Paleobiology in Patagonia: High-latitude Paleocommunities of the Santa Cruz Formation. Cambridge University Press.

Guérin, C, M. A. Curvello, M. Faure, M. Hugueney & C. Mourer-Chauviré 1996 The Pleistocene fauna of Piauí (Northeastern Brazil): paleochronological and biochronological implications. Proceedings of the International Meeting on the Peopling of the Americas, São Raimundo Nonato, Piauí, Brasil (1993). Fundhamentos 1(1):55-103.

## NEOGNATHAE

## GALLOANSERES

**Calibration: crown Galloanseres**

**Category: A**

**MRCA of: Galliformes and Anseriformes**

**Clade definition in tree:** *Gallus gallus, Anas platyrhynchos*

**Oldest fossil:** *Conflicto antarticus* Tambussi *et al.*, 2019 a nearly complete skeleton (holotype MLP 07-III-1-1) from Level 10 of the López de Bertodano Formation at Seymour Island, Antarctica.

**Phylogenetic placement justification:** A cladistic analysis including a variety of extant and extinct galloanserines placed the holotype of *Conflicto antarticus* as a stem anseriform (Tambussi *et al.* 2019), a result confirmed independently by cladistic and Bayesian analyses of a similarly diverse dataset (Field *et al.* 2020).

**Minimum age:** 65.65 Ma

**Minimum age justification:** Level 10 of the López de Bertodano Formation is the youngest stratum of the formation and sits right above the K-Pg boundary (66.04 Ma); its upper bound was estimated as 65.65 Ma based on based on dinoflagellate cyst biostratigraphy and U–Pb zircon dating (Bowman *et al.* 2016).

**First occurrences:**

- *Conflicto antarticus* Tambussi *et al*., 2019 from Level 10 (early Paleocene, 66.04–65.65 Ma, Bowman *et al.* 2016) of the López de Bertodano Formation at Seymour Island, Antarctica
- *Gastornis* sp., fragments of femur shaft from the Orp Sand Member of the Heers Formation (early to middle Selandian, 61.7–60 Ma) at Maret, Belgium (Mayr & Smith 2019).
- Unnamed anseriform, partial quadrate (QM F23019) from the Tingamarra Local Fauna (early Eocene, 54.65–54.55 Ma) of Murgon, Queensland, Australia (Elzanowski & Boles 2012)
- *Presbyornis mongoliensis* Kurochkin & Dyke, 2009, paratypes from Tsagaan Khushuu (Paleocene, 58.7–55.8 Ma), Mongolia
- *Presbyornis pervetus* Wetmore, 1926 numerous specimens from the Fossil Butte Member (early Eocene, 52.13–51.81 Ma) of the Green River Formation, Wyoming, USA.
- *Telmabates antiquus* Howard, 1955 and *T. howardae* Cracraft, 1970 from the Eocene (Casamayoran, 55.8–48 Ma) of Patagonia, Argentina.
- *Namaortyx sperrgebietensis* Mourer-Chauvire *et al.*, 2011 from Silica South (Middle Eocene, 49–47 Ma), Sperrgebiet, Namibia.
- *Manuherikia lacustrina* Worthy *et al*., 2007, isolated long bones from Bannockburn Formation (early Miocene, Altonian, 18.26–15.97 Ma), Otago, New Zealand (Worthy & Lee 2008).
- *Alopochen sirabensis* from Ampasambazimba (Holocene, 0.0229–0.0222 Ma, radiocarbon dated), Madagascar (Goodman 1999).

**Fossil record remarks:**

**Clade age estimation:** Neogene fossils (*Manuherikia lacustrina* and *Alopochen sirabensis*) excluded from the estimation to improve record uniformity. Kolmogorov-Smirnov test before exclusion: D = 0.44, p-value = 0.039.

One-sample Kolmogorov-Smirnov test

data: Mages

D = 0.21158, p-value = 0.8542

alternative hypothesis: two-sided

Quantiles:

0% 50% 95% 97.5%

65.68 67.74 75.60 78.11

Parameters of the skewStudent function:

offset xi omega alpha nu

65.681 65.787 2.517 42.974 2.335

**
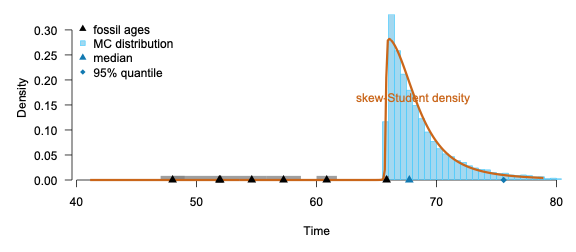
**

**References:**

Bowman, V., J. Ineson, J. Riding, J. Crame, J. Francis, D. Condon, R. Whittle & F. Ferraccioli. 2016. The Paleocene of Antarctica: Dinoflagellate cyst biostratigraphy, chronostratigraphy and implications for the palaeo-Pacific margin of Gondwana. Gondwana Research 38:132-148.

Cracraft. 1970. A new species of Telmabates (Phoenicopteriformes) from the lower Eocene of Patagonia. Condor 72:479-480.

Elzanowski A, Boles WE. 2012 Australia’s oldest Anseriform fossil: a quadrate from the Early Eocene Tingamarra Fauna. Palaeontology 55, 903–911.

Field, D. J., J. Benito, A. Chen, J. W. M. Jagt, D. T. Ksepka 2020. Late Cretaceous neornithine from Europe illuminates the origins of crown birds. Nature 579 (7799): 397–401.

Goodman, S.M. 1999. Holocene bird subfossils from the sites of Ampasambazimba, Antsirabe and Ampoza, Madagascar: Changes in the avifauna of south-central Madagascar over the past few millennia. In: Adams, N.J. & Slotow, R.H. (eds) Proc. 22 Int. Ornithol. Congr., Durban: 3071-3083. Johannesburg: BirdLife South Africa.

Howard, H. 1955. A new wading bird from the Eocene of Patagonia. American Museum Novitates 1710:1-25.

Kurochkin E. N. & A. A. Dyke 2009. A large collection of *Presbyornis* (Aves, Anseriformes, Presbyornithidae) from the late Paleocene and early Eocene of Mongolia. Geological Journal 45:375-387.

Mayr, G., & T. Smith 2019. New Paleocene bird fossils from the North Sea Basin in Belgium and France. Geologica Belgica 22:35-46.

Mourer-Chauvire, Pickford & Senut 2011. The first Palaeogene Galliform from Africa. Journal of Ornithology 152 3:617-622

Tambussi, C.P., F.J. Degrange, R.S. De Mendoza, E. Sferco & S. Santillana. 2019. A stem anseriform from the early Palaeocene of Antarctica provides new key evidence in the early evolution of waterfowl. Zoological Journal of the Linnean Society 186(3):673-700. doi: 10.1093/zoolinnean/zly085

Wetmore, A. (1926): Fossil birds from the Green River Deposits of Eastern Utah. Annals of the Carnegie Museum 16: 391–402.

Worthy, T. H., A. J. D. Tennyson, C. Jones, J. A. McNamara & B. J. Douglas. 2007. Miocene waterfowl and other birds from Central Otago, New Zealand. Journal of Systematic Palaeontology 5:1–39.

Worthy T. H., & M. S. Y. Lee. 2008. Affinities of Miocene (19–16 Ma) waterfowl (Anatidae: *Manuherikia*, *Dunstanetta* and *Miotadorna*) from the St Bathans Fauna, New Zealand. Palaeontology 51: 677–708.

**Calibration: crown Anatidae**

**Category: A**

**MRCA of: Anatinae and Anserinae**

**Clade definition in tree:** *Anas platyrhynchos, Anser cygnoides*

**Oldest fossil:** *Pinpanetta tedfordi* Worthy, 2009, complete right humerus (holotype SAMA P.41257) from the Etadunna Formation at Young Bucks Quarry, Lake Palankarinna, South Australia, Australia. Shared with three other contemporaneous fossil ducks: *Pinpanetta vickersrichae* and *Pinpanetta fromensis*, and *Australotadorna alecwilsoni* (Worthy 2009).

**Phylogenetic placement justification:** A cladistic analysis of *Pinpanetta* (including *P. tedfordi*) placed it as a stem member of the Erismaturinae (Worthy 2009). Given the uncertainty in the relationships among anatid subfamilies, this fossil is used to calibrate crown Anatidae.

**Minimum age:**  24 Ma

**Minimum age justification:** Biostratigraphy of fossil land mammals, presence of Oligocene foraminiferal fauna, and magnetostratigraphy indicate an age between 24 and 26 Ma for the Etadunna Formation (Woodburne *et al.* 1994).

**Additional remarks:** Two additional species described in the same genus from the same age (Worthy 2009) further support this calibration.

**First occurrences:**

- *Pinpanetta tedfordi* Worthy, 2009 from Faunal Zone A of the Etadunna Formation (late Oligocene, 26–25.2 Ma) at Young Bucks Quarry, Lake Palankarinna, South Australia, Australia.
- *Mionetta blanchardi* (Milne-Edwards, 1863), multiple bones from several individuals from Saint-Gérand-le-Puy (Aquitanian, 23–20.4 Ma), Allier, France (Worthy & Lee 2008)
- *Mionetta* sp., distal humerus from Grillental (early Miocene, 20 Ma), Northern Sperrgebiet, Namibia (Mourer-Chauviré 2008).
- *Manuherikia lacustrina* Worthy *et al.* 2007, isolated long bones from the Bannockburn Formation, St Bathans Fauna (early Miocene, Altonian, 18.26–15.97 Ma), Otago, New Zealand (Worthy *et al.* 2007; Worthy & Lee 2008).
- *Sharganetta mongolica* Zelenkov, 2011, *Nogusunna conflictoides* Zelenkov, 2011, *Protomelanitta gracilis* Zelenkov, 2011, *Chenoanas deserta* Zelenkov, 2012, multiple bone fragments representing multiple genera from Sharga (Middle Miocene, NMU7, MN7–8, 13.1–11.2 Ma), Shargyn-Govi area, Mongolia (Zelenkov 2011).
- *Megalodytes morejohni* Howard, 1992 from the Round Mountain Member of the Temblor Formation (Langhian, 15.99–11.63 Ma), Kern County, California, USA.
- *Chloephaga robusta* Tambussi, 1998, distal ulna from the Irene Formation (early Pliocene, 4–3 Ma), Buenos Aires, Argentina.
- *Alopochen sirabensis* from the Pleistocene of Ampasambazimba (0.0229–0.0222 Ma), Madagascar (Goodman 1999).

**Fossil record remarks:**

**Clade age estimation:**

One-sample Kolmogorov-Smirnov test

data: Mages

D = 0.16468, p-value = 0.9578

alternative hypothesis: two-sided

Quantiles:

0% 50% 95% 97.5%

25.23 27.91 37.27 40.36

Parameters of the skewStudent function:

offset xi omega alpha nu

25.229 25.486 3.243 22.053 2.566

**
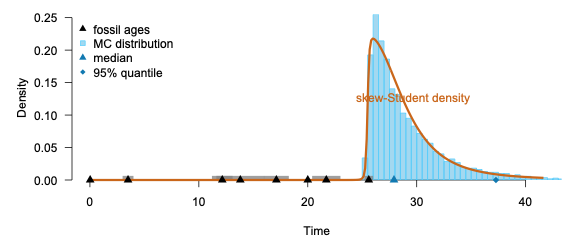
**

**References:**

Goodman, S.M. 1999. Holocene bird subfossils from the sites of Ampasambazimba, Antsirabe and Ampoza, Madagascar: Changes in the avifauna of south central Madagascar over the past few millennia. In: Adams, N.J. & Slotow, R.H. (eds) Proc. 22 Int. Ornithol. Congr., Durban: 3071-3083. Johannesburg: BirdLife South Africa.

Howard, H. (1992). New records of middle Miocene anseriform birds from Kern County, California. Papers in Avian Paleontology Honoring Pierce Brodkorb. Natural History Museum of Los Angeles County, Science Series, 36, 231-237

Mourer-Chauviré, C. (2008). Birds (Aves) from the Early Miocene of the Northern Sperrgebiet, Namibia. Geology and Palaeobiology of Namib Desert, Southwestern Africa, 3, 147-167.

Tambussi C. P. 1998. Nuevo Anatidae (Aves: Anseriformes) del Plioceno de la región pampeana, Argentina. Boll. Soc Hist. Nat. Balears 41: 19-25.

Woodburne, M. O., B. J. MacFadden, J. A. Case, M. S. Springer, N. S. Pledge, J. D. Power, J. M. Woodburne & K. B. Springer. 1994. Land mammal biostratigraphy and magnetostratigraphy of the Etadunna Formation (late Oligocene) of South Australia. Journal of Vertebrate Paleontology 13(4):483-515.

Worthy, T. H., A. J. D. Tennyson, C. Jones, J. A. McNamara, and B. J. Douglas. 2007. Miocene waterfowl and other birds from Central Otago, New Zealand. Journal of Systematic Palaeontology 5:1–39.

Worthy T.H. & M. S. Y. Lee. 2008. Affinities of Miocene (19–16 Ma) waterfowl (Anatidae: *Manuherikia*, *Dunstanetta* and *Miotadorna*) from the St Bathans Fauna, New Zealand. Palaeontology 51: 677–708.

Worthy, T. H. 2009. Descriptions and phylogenetic relationships of two new genera and four new species of Oligo-Miocene waterfowl (Aves: Anatidae) from Australia. Zoological Journal of the Linnean Society 156: 411–454.

Zelenkov, N. V. (2011). Diving ducks from the Middle Miocene of western Mongolia. Paleontological Journal, 45(2), 191-199.

Zelenkov, N.V. 2012. A new duck from the Middle Miocene of Mongolia, with comments on Miocene evolution of ducks. Paleontol. J. 46: 520–530.

## NEOAVES

## PHOENICOPTERIMORPHAE

**Calibration: crown Phoenicopterimorphae**

**Category: B**

**MRCA of:** Phoenicopteriformes and Podicipediformes

**Clade definition in tree:** *Phoenicopterus ruber*, *Podiceps cristatus*

**Oldest fossil:** *Adelalopus hoogbutseliensis* Mayr & Smith, 2002, fragmentary bones (holotype IRScNB Av 71) from the Early Oligocene, MP 21, of Hoogbutsel near Boutersem, Brabant, Belgium.

**Phylogenetic placement justification:** Assigned to the Palaelodidae based on four presumed derived traits shared with the family, in addition to an overall similar morphology (Mayr & Smith 2002).

**Minimum age:** 32.6 Ma

**Minimum age justification:** The deposits of Hoogbutsel correlate with the MP 21 zone (Mayr & Smith 2002).

**First occurrences:**

- *Adelalopus hoogbutseliensis* Mayr & Smith 2002 from the Early Oligocene (MP21, 33.8–32.6 Ma) of Hoogbutsel near Boutersem, Barbant, Belgium (Mayr & Smith 2002).
- cf. *Palaelodus* from the Jebel Qatrani Formation at Quarry M (early Oligocene, (30.2–29.5 Ma, Seiffert 2006), Fayum, Egypt (Rasmussen *et al.* 1987).
- *Phoeniconotius eyrensis* Miller, 1963 from the Etadunna Formation (Faunal Zone A) (late Oligocene 26–25.2 Ma), South Australia, Australia.
- *Palaelodus* cf. *ambiguus* from the Tremembé Formation (late Oligocene-Early Miocene (28.1–23 Ma) at Taubaté, São Paulo, Brasil (Alvarenga 1990).
- *Megapaloelodus connectens* Miller, 1944 from the early Miocene (23.03–15.97 Ma) of South Dakota, USA.
- *Palaelodus aotearoa* Worthy *et al.*, 2010 from the Bannockburn Formation, St Bathans Fauna (early Miocene, Altonian, 18.26–15.97 Ma), Otago, New Zealand.
- *Palaelodus kurochkini* Zelenkov, 2013 from the Middle Miocene of Sharga (NMU7, MN7–8, 13.1–11.2 Ma), Shargyn-Govi area, Mongolia.
- *Phoenicopterus ruber* from Ampoza cave deposits (Holocene, 0.002–0 Ma) in Madagascar (Goodman 1999).

**Fossil record remarks:**

**Clade age estimation:**

One-sample Kolmogorov-Smirnov test

data: Mages

D = 0.22194, p-value = 0.75

alternative hypothesis: two-sided

Quantiles:

0% 50% 95% 97.5%

32.64 36.28 48.46 52.35

Parameters of the skewStudent function:

offset xi omega alpha nu

32.637 33.035 4.336 20.740 2.654

**
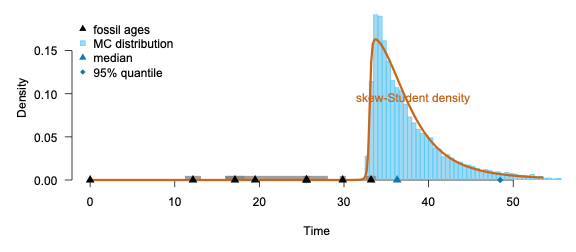
**

**References:**

Alvarenga, H. M. F. 1990. Flamingos fosseis da bacia de Taubaté, estado de São Paulo, Brasil: desçricao de nova especie. An. Acad. Brasileira de Ciências 62:335–345.

Goodman, S.M. 1999. Holocene bird subfossils from the sites of Ampasambazimba, Antsirabe and Ampoza, Madagascar:Changes in the avifauna of south central Madagascar over the past few millennia. In: Adams, N.J. & Slotow, R.H. (eds) Proc. 22 Int. Ornithol. Congr., Durban: 3071-3083. Johannesburg: BirdLife South Africa.

Mayr, G. & Smith, R. 2002. Avian remains from the lowermost Oligocene of Hoogbutsel (Belgium). Bulletin de l'Institut Royal des Sciences Naturelles de Belgique 72: 139-150.

Miller, A. H. 1944. An avifauna from the Lower Miocene of South Dakota. University of California Publications, Bulletin of the Department of Geological Sciences 27: 85–100.

Miller, A. H. 1963 Fossil flamingos from Australia. Condor 65:289-299.

Rasmussen, D. T., Olson, S. L. & E. L. Simons. 1987. Fossil birds from the Oligocene Jebel Qatrani Formation, Fayum Province, Egypt. Smithsonian Contributions to Paleobiology 62:1-20.

Seiffert, E. R. 2006. Revised age estimates for the later Paleogene mammal faunas of Egypt and Oman. Proceedings of the National Academy of Sciences of the United States of America 103:5000-5005.

Worthy, T. H., A.J.D. Tennyson, M. Archer & R. P. Scofield 2010. First record of Palaelodus (Aves: Phoenicopteriformes) from New Zealand. Rec.Austral. Mus. 62:77–88.

Zelenkov, N. V. 2013. Cenozoic Phoenicopteriform Birds from Central Asia. Paleontological Journal 47(11):1323–1330.

## OTIDIMORPHAE

**Calibration: crown Otidimorphae (sensu Houde, Cracraft & Braun 2019)**

**Category: A**

**MRCA of:** Otidiformes, Musophagiformes and Cuculiformes

**Clade definition in tree:** *Tauraco erythrolophus, Ardeotis kori*

**Oldest fossil:** *Foro panarium* Olson, 1992, nearly complete skeleton (holotype USNM 336621) from the Fossil Butte Member of the Green River Formation at Thompson Quarry, Lincoln County, Wyoming, USA.

**Phylogenetic placement justification:** *Foro panarium* referred as sister taxon to Musophagidae by the presence of these synapomorphies: “unfused midline of furcula … processus costales of axis absent … bill short and stout with broad processus maxillaris of the os nasale … trochlea metatarsi IV without large trochlea accessoria … tendon of musculus flexor hallucis longus not enclosed in bony canal” identified by inclusion in two different topologically constrained phylogenetic analyses using the dataset from Mayr *et al.* (2011) with two additional characters added (Field and Hsiang, 2018: pp. 2-3).

**Minimum age:** 51.81 Ma

**Minimum age justification:** Radiometric ^40^Ar/^39^Ar-dating of the K-spar tuff near the top of the Fossil Butte member yielded a recalibrated date of 51.97±0.16 Ma (Smith *et al.*, 2008, 2010).

**First occurrences:**

- *Foro panarium* Olson, 1992, nearly complete skeleton (holotype USNM 336621) from the Fossil Butte Member (52.13–51.81 Ma) of the Green River Formation at Thompson Quarry, Lincoln County, Wyoming, USA.
- *Chambicuculus pusillus* Mourer-Chauviré *et al.*, 2013, represented by fragments of multiple bones from the Eocene (51–44.6 Ma, Coster *et al.* 2012) of Djebel Chambi, Tunisia.
- *Eocuculus* cf. *cherpinae* Chandler, 1999, partial postcranial skeleton from the Early Oligocene (MP24, 30–28.8 Ma) of Pichovet, France (Mayr 2006).
- Otididae indet. from the Oligocene (33.9–23.0 Ma) of Tchelkar Nura, Kazakhstan (Kurochkin 1976).

**Fossil record remarks:** Only the Paleogene fossil record is considered here in order to ensure uniformity. All first occurrences in other continents are in the Quaternary.

**Clade age estimation:**

One-sample Kolmogorov-Smirnov test

data: Mages

D = 0.31854, p-value = 0.7115

alternative hypothesis: two-sided

Quantiles:

0% 50% 95% 97.5%

51.83 56.68 78.43 87.32

Parameters of the skewStudent function:

offset xi omega alpha nu

51.827 51.922 5.989 97.447 1.933

**
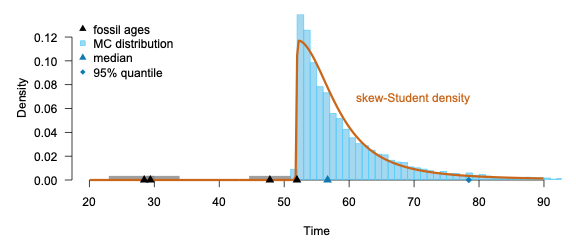
**

**References:**

Coster, P., Benammi, M., Mahboubi, M., Tabuce, R., Adaci, M., Marivaux, L., Bensalah, M., Mahboubi, S., Mahboubi, A., Mebrouk, F. & Maameri, C. 2012. Chronology of the Eocene continental deposits of Africa: Magnetostratigraphy and biostratigraphy of the El Kohol and Glib Zegdou Formations, Algeria. Geological Society of America Bulletin 124(9-10):1590-1606.

Field, D.J. & Hsiang, A.Y. (2018): A North American stem turaco, and the complex biogeographic history of modern birds. *BMC Evolutionary Biology* **18** (102), pp 1–16

Kurochkin, E. N. 1976. A survey of the Paleogene birds of Asia. Smithson. Contrib. Paleobiol. 27:75-86.

Mayr, G. (2006). A specimen of Eocuculus Chandler, 1999 (Aves, ?Cuculidae) from the early Oligocene of France. Geobios, 39(6), 865-872.

Mayr, G., Alvarenga, H. & Mourer-Chauviré, C. (2011): Out of Africa: Fossil shed light on the origin of the hoatzin, an iconic neotropic bird. *Naturwissenschaften* **98**, pp 961-966

Mourer-Chauviré, C., Essid, E.M., Khayati, H., Marivaux, L., Marzougui, W., Temani, R., Vianey-Liaud, M. & Tabuce, R. 2016. New remains of the very small cuckoo, *Chambicuculus pusillus* (Aves, Cuculiformes, Cuculidae) from the late Early or early Middle Eocene of Djebel Chambi, Tunisia. Palaeovertebrata 40(1):1-4.

Olson, S.L. (1992): A new family of primitive landbirds from the Lower Eocene Green River Formation of Wyoming. *Natural History Museum of Los Angeles County Science Series* **36**, pp 127-136

Smith, M.E., Carroll, A.R., & Singer, B.S. 2008 Synoptic reconstruction of a major ancient lake system: Eocene Green River Formation, western United States. *Geological Society of America Bulletin* **120** (1/2), pp 54-84

Smith, M.E., Chamberlain, K.R., Singer, B.S. & Carroll, A.R. (2010): Eocene clocks agree: coeval ^40^Ar/^39^Ar, U-Pb, and astronomical ages from the Green River Formation. *Geology* **38** (6), pp 527-530.

## STRISORES

**Calibration: crown Daedalornithes**

**Category: A**

**MRCA of: Aegothelidae and Apodiformes**

**Clade definition in tree:** *Aegotheles bennettii, Chaetura pelagica*

**Oldest fossil:** *Eocypselus vincenti* Harrison, 1984, referred material, a nearly complete skeleton (MGUH 29278) and two incomplete postcranial skeletons (MGUH 26729, MGUH 26730) from the Fur Formation at Isle-of-Mors, Denmark (Dyke *et al.* 2004, Mayr 2010).

**Phylogenetic placement justification:** A cladistic analysis (Ksepka et al. 2013) and Bayesian analyses with topological constraints (Chen *et al.* 2019) found *Eocypselus vincenti* as a stem Apodiformes, but a total evidence Bayesian analysis indicated a position as stem Aegothelidae instead (Chen *et al.* 2019). Either way, *Eocypselus* is confirmed as a basal crown Daedalornithes.

**Minimum age:** 54.6 Ma.

**Minimum age justification:** The bottom of the Fur Formation was deposited during the recovery phase of the Paleocene-Eocene Thermal Maximum around 55.8 Ma ago (Stokke et al. 2020a). Basalts at the top of the Formation correlate with “subphase 2b” of pyroclastic deposition in the Balder Formation, suggesting a minimum age of 54.6 Ma (King 2016, Stokke et al. 2020b).

**First occurrences:**

- *Eocypselus vincenti* Harrison, 1984 from the early Eocene (55.8–54.6 Ma) Fur Formation, Denmark.
- *Eocypselus rowei* Ksepka *et. al*, 2013 from the Fossil Butte Member of the Green River Formation (Early Eocene, 51.97±0.16 Ma), Wyoming, USA.
- *Collocalia buday* Boles, 2001 from the early Miocene Camel Sputum Site (18.53–16.97 Ma, Woodhead et al. 2016), Riversleigh, Queensland, Australia.
- *Aegotheles* *zealandivetus* Worthy et al. 2022 from the Bannockburn Formation, St Bathans Fauna (Early Miocene, Altonian, 18.26–15.97 Ma), Otago, New Zealand.
- *Tachymarptis* sp. from the upper Varswater Formation at Langebaanweg (Early Pliocene 5.33–3.6 Ma), Cape Province, South Africa (Manegold *et al*. 2013).
- *Apus pacificus* from the Late Mousterian of the Ust'-Kanskaya Peshchera caves (Holocene, 0.05 Ma), Gorno-Altai, Russia (Tyrberg 1998).
- *Streptoprocne zonaris* from the Late Pleistocene (0.129–0.0117 Ma) of Piauí, Brazil (Guérin *et al.* 1996).

**Fossil record remarks:** A record of *Apus* sp. from Madagascar (Burney *et al.* 2008) was not used as *Apus pacificus* is already included in the set.

**Clade age estimation:**

One-sample Kolmogorov-Smirnov test

data: Mages

D = 0.43177, p-value = 0.106

alternative hypothesis: two-sided

Quantiles:

0% 50% 95% 97.5%

54.65 61.02 85.50 95.65

Parameters of the skewStudent function:

offset xi omega alpha nu

54.649 55.022 7.722 37.041 2.233

**
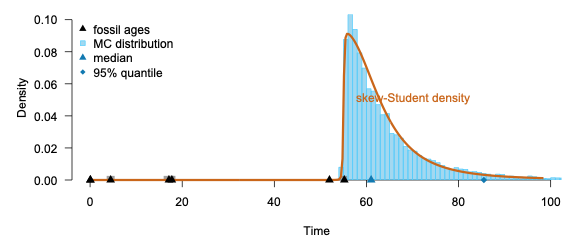
**

**References:**

Boles, W. E., 2001. A swiftlet (Apodidae: Collocaliini) from the Miocene of Riversleigh, northwestern Queensland. Memoir of the Association of Australasian Palaeontologists 25: 45–52.

Burney, D. A., Vasey, N., Godfrey, L. R., Jungers, W. L., Ramarolahy, M. F., & Raharivony, L. L. 2008. New findings at Andrahomana Cave, southeastern Madagascar. Journal of Cave and Karst Studies 70(1):13–24.

Chen, A., N. D. White, R. B. J. Benson, M. J. Braun & D. J. Field. 2019. Total-evidence framework reveals complex morphological evolution in nightbirds (Strisores). Diversity 11(9):143.

Dyke, G. J., D. M. Waterhouse & A. V. Kristoffersen 2004 Three new fossil landbirds from the early Paleogene of Denmark. Bulletin of the Geological Society of Denmark 51:77–85.

Dyke & Lindow 2009. Taphonomy and abundance of birds from the lower Eocene Fur Formation Denmark. Geological Journal 44:365-373.

Harrison, C. J. O. 1984. A revision of the fossil swifts (Vertebrata, Aves, suborder, Apodi), with descriptions of three new genera and two new species. Mededelingen van de Werkgroep voor Tertiaire en Kwartaire Geologie 21(4):157-177.

Guérin, C, M. A. Curvello, M. Faure, M. Hugueney & C. Mourer-Chauviré 1996 The Pleistocene fauna of Piauí (Northeastern Brazil): paleochronological and biochronological implications. Proceedings of the International Meeting on the Peopling of the Americas, São Raimundo Nonato, Piauí, Brasil (1993). Fundamentos 1(1):55-103.

King, C. 2016. A revised correlation of Tertiary rocks in the British Isles and adjacent areas of NW Europe. Geol. Soc. Lond. Spec. Rep. 27: 1–719.Ksepka, D. T., J. A. Clarke, S. J. Nesbitt, F. B. Kulp & L. Grande. 2013 Fossil evidence of wing shape in a stem relative of swifts and hummingbirds (Aves, Pan-Apodiformes). Proc. R. Soc. B 280: 20130580.

Manegold, A., A. Louchart, J. Carrier & A. Elzanowski 2013 The early Pliocene avifauna of Langebaanweg (South Africa): a review and update. In Paleornithological Research 2013: Proceedings of the 8th International Meeting of the Society of Avian Paleontology and Evolution pp. 135-152.

Mayr, G. 2010. Reappraisal of *Eocypselus*--a stem group apodiform from the early Eocene of Northern Europe. Paleobiology & Paleoenvironment 90:395-403.

Stokke, E. W., E. Liu & M. T. Jones 2020a. Evidence of explosive hydromagmatic eruptions during the emplacement of the North Atlantic Igneous Province. Volcanica 3(2):227-250.

Stokke, E. W., M. T. Jones, J. E. Tierney, H. H. Svensen, & J. H. Whiteside 2020b. Temperature changes across the Paleocene-Eocene Thermal Maximum – a new high-resolution TEX86 temperature record from the Eastern North Sea Basin. Earth and Planetary Science Letters 544:116388. doi: 10.1016/j.epsl. 2020.116388.

Tyrberg 1998 Pleistocene birds of the Palearctic: a catalogue. Nuttall Ornithological Club No. 27, Cambridge, Mass.

Woodhead, J., S. J. Hand, M. Archer, I. Graham, K. Sniderman, D. A. Arena, K. H. Black, H. Godthelp, P. Creaser & Price, E. (2016). Developing a radiometrically-dated chronologic sequence for Neogene biotic change in Australia, from the Riversleigh World Heritage Area of Queensland. Gondwana Research 29(1):153-167.

Worthy, T. H., R. P. Scofield, S. W. Salisbury, S. J. Hand,V. L. De Pietri, & M. Archer. 2022. Two new neoavian taxa with contrasting palaeobiogeographical implications from the early Miocene St Bathans Fauna, New Zealand. Journal of Ornithology https://doi.org/10.1007/s10336-022-01981-6.

**Calibration: crown Apodi**

**Category: A**

**MRCA of: Apodidae and Hemiprocnidae**

**Clade definition in tree:** *Hemiprocne comata, Chaetura pelagica*

**Oldest fossil:** *Scaniacypselus wardi* Harrison, 1984, articulated pectoral girdle and forelimbs (holotype BMNH A5430) from Member R6 of the Røsnæs Clay Formation in Ølst, Denmark.

**Phylogenetic placement justification:** Cladistic analyses (Mayr 2003, Ksepka *et al.* 2013) and Bayesian analyses with topological constraints (Chen *et al.* 2019) found *Scaniacypselus wardi* as a stem Apodidae.

**Minimum age:** 50.3 Ma.

**Age justification:** The top of Member R6 is overlaid conformably by the Lillebælt Clay Formation; its base coincides with a sharp transition from calcareous to non-calcareous foraminifera, including *Reticulophragmium amplectens*, which correlates with the NS18-NS19 boundary at around 50.3 Ma (King 2016, fig. 76). The lowest part of R6 (or the top of the underlying Member R5) shows the last occurrence of the dinoflagellate cysts *Ochetodinium romanum*, which represent the top of Subzone DE8b (King 2016) at around 51.5 Ma (Speijer *et al.* 2020). King (2016) shows a very narrow Subzone DE8b containing *Ochetodinium romanum* and right after the polarity reversal in the middle of C23, which is 51.72 Ma (Speijer *et al.* 2020).

**First occurrences:**

- *Scaniacypselus wardi* Harrison, 1984, articulated pectoral girdle and forelimbs (BMNH A5430) from Bed R6 (50.3–51.5 Ma) of the Røsnæs Clay Formation in Ølst, Denmark.
- *Collocalia buday* Boles, 2001 from the early Miocene Camel Sputum Site (18.53–16.97 Ma, Woodhead *et al.* 2016), Riversleigh, Queensland, Australia.
- *Collocalia* sp. from the Bannockburn Formation, St Bathans Fauna (Early Miocene, Altonian, 18.26–15.97 Ma), Otago, New Zealand (Worthy *et al.* 2007). Although not identified at the species level, it is estimated to be much smaller than *Collocalia buday* (Worthy *et al.* 2007).
- *Tachymarptis* sp. from the upper Varswater Formation at Langebaanweg (Early Pliocene 5.33–3.6 Ma), Cape Province, South Africa (Manegold *et al.* 2003).
- *Apus pacificus* from the Late Pleistocene (Late Mousterian) of the Ust'-Kanskaya Peshchera caves in Gorno-Altai, Russia (Tyrberg 1998).
- *Streptoprocne zonaris* from the Late Pleistocene (0.129–0.0117 Ma) at Barra do Antonião, Piauí, Brazil (Guérin *et al.* 1996).
- *Chaetura pelagica* from the Late Pleistocene (0.1–0.0117 Ma) of Clark's Cave, Bath County, Virginia, USA (Guilday *et al.* 1977).

**Fossil record remarks:** A record of *Apus* sp. from Madagascar (Burney et al. 2008) was not used as *Apus pacificus* is already included in the set. Quaternary record excluded to preserve uniformity.

**Clade age estimation:**

One-sample Kolmogorov-Smirnov test

data: Mages

D = 0.4552, p-value = 0.2799

alternative hypothesis: two-sided

Quantiles:

0% 50% 95% 97.5%

50.32 59.82 103.40 121.40

Parameters of the skewStudent function:

offset xi omega alpha nu

50.32 50.68 11.39 61.85 1.90


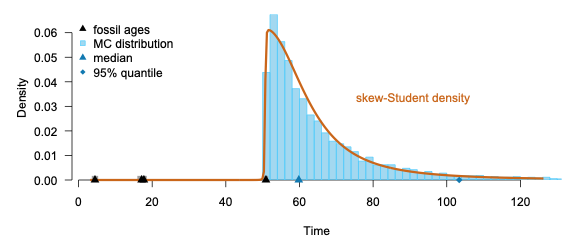


**References:**

Boles, W. E., 2001. A swiftlet (Apodidae: Collocaliini) from the Miocene of Riversleigh, northwestern Queensland. Memoir of the Association of Australasian Palaeontologists 25: 45–52.

Chen, A., N. D. White, R. B. J. Benson, M. J. Braun & D. J. Field. 2019. Total-evidence framework reveals complex morphological evolution in nightbirds (Strisores). *Diversity* 11(9):143.

Guérin, C, M. A. Curvello, M. Faure, M. Hugueney & C. Mourer-Chauviré 1996 The Pleistocene fauna of Piauí (Northeastern Brazil): paleochronological and biochronological implications. Proceedings of the International Meeting on the Peopling of the Americas, São Raimundo Nonato, Piauí, Brasil (1993). Fundamentos 1(1):55-103.

Guilday, J. E., P. W. Parmalee, and H. W. Hamilton. 1977. The Clark's Cave bone deposit and the Late Pleistocene paleoecology of the central Appalachian Mountains of Virginia. Bulletin of Carnegie Museum of Natural History 2:1-87.

Harrison, C. J. O. 1984. A revision of the fossil swifts (Vertebrata, Aves, suborder, Apodi), with descriptions of three new genera and two new species. Mededelingen van de Werkgroep voor Tertiaire en Kwartaire Geologie 21(4):157-177.

King, C. 2016. A revised correlation of Tertiary rocks in the British Isles and adjacent areas of NW Europe. Geol. Soc. Lond. Spec. Rep. 27: 1–719.

Ksepka, D. T., J. A. Clarke, S. J. Nesbitt, F. B. Kulp & L. Grande. 2013 Fossil evidence of wing shape in a stem relative of swifts and hummingbirds (Aves, Pan-Apodiformes). Proc. R. Soc. B 280: 20130580.

Manegold, A., A. Louchart, J. Carrier & A. Elzanowski 2013 The early Pliocene avifauna of Langebaanweg (South Africa): a review and update. In Paleornithological Research 2013: Proceedings of the 8th International Meeting of the Society of Avian Paleontology and Evolution pp. 135-152.

Mayr, G. 2003 Phylogeny of early Tertiary swifts and hummingbirds (Aves: Apodiformes). Auk 120:145-151.

Speijer, R. P., Pälike, H., Hollis, C. J., Hooker, J. J., & Ogg, J. G. 2020. The Paleogene Period. In F. M. Gradstein, J. G. Ogg, M. D. Schmitz, & G. M. Ogg (Eds.), Geologic Time Scale 2020 (pp. 1087–1140). Elsevier B.V.: Amsterdam.

Tyrberg 1998 Pleistocene birds of the Palearctic: a catalogue. Nuttall Ornithological Club No. 27, Cambridge, Mass.

Woodhead, J., S. J. Hand, M. Archer, I. Graham, K. Sniderman, D. A. Arena, K. H. Black, H. Godthelp, P. Creaser & Price, E. (2016). Developing a radiometrically-dated chronologic sequence for Neogene biotic change in Australia, from the Riversleigh World Heritage Area of Queensland. Gondwana Research 29(1):153-167.

Worthy, T. H., A. J. D. Tennyson, C. Jones, J. A. McNamara, and B. J. Douglas. 2007. Miocene waterfowl and other birds from Central Otago, New Zealand. Journal of Systematic Palaeontology 5:1–39.

## CURSORIMORPHAE

**Calibration: crown Gruiformes**

**Category: B**

**MRCA of: Gruoidea and Ralloidea**

**Clade definition in tree:** *Grus americana*, *Heliornis fulica*

**Oldest fossil:** *Pellornis mikkelseni* Bertelli *et al.*, 2011, a nearly complete articulated skeleton (holotype MGUH 29278) from the Fur Formation near Sundby, Isle of Mors, Jutland, Denmark (Hoch 1997, Musser *et al.* 2019).

**Phylogenetic placement justification:** Initial cladistic analyses indicated a position of Messelornithidae, including *Pellornis mikkelseni*, as stem Rallidae (Bertelli *et al.* 2011). More recent analyses including additional characters from a more complete preparation of the holotype revealing a complete skull among other parts, indicated a more basal position as stem Ralloidea (Musser *et al.* 2019, Musser & Clarke 2020), thus setting a minimum age for crown Gruiformes.

**Minimum age:** 55.6 Ma

**Minimum age justification:** MGUH 29278 is preserved in silicified diatomite, and thus must derive from one of two horizons near ash layers -19 or -21 in the lower part of the Knudeklint Member. The bottom of the Fur Formation was deposited during the recovery phase of the Paleocene-Eocene Thermal Maximum around 55.8 Ma ago (Stokke *et al.* 2020a). The younger ash layer -17 has been dated at 55.6 ± 0.12 Ma (corrected ^40^Ar/^39^Ar ages, Stokke *et al.* 2020b), providing a minimum age for the fossil.

**First occurrences:**

- *Pellornis mikkelseni* Bertelli *et al.*, 2011, a nearly complete articulated skeleton (holotype MGUH 29278) (Musser *et al.* 2019), from the Fur Formation near Sundby (55.8–55.6 Ma), Isle of Mors, Jutland, Denmark.
- *Songzia acutunguis* Hou, 1990, articulated skeletons (Wang *et al.* 2012) from the Yangxi Formation (55.8–48.6 Ma), China, phylogenetically placed in the Messelornithidae by cladistic analysis (Musser *et al.* 2019).
- *Messelornis nearctica* Hesse, 1992, several skeletons (Weidig 2010) from the Fossil Butte Member of the Green River Formation (51.97 ± 0.16 Ma), Wyoming, USA. See Musser *et al.* (2019) for phylogenetic analysis.
- Indeterminate Rallidae from the Jebel Qatrani Formation (early Oligocene, 33.9–28.4 Ma) at Fayum, Egypt, (Rasmussen *et al.* 1987).
- *Australlus disneyi* (Boles, 2005), specimens from White Hunter Site (Faunal Zone A, late Oligocene, 26–23.04 Ma), Riversleigh, Queensland, Australia. Phylogenetic position determined by cladistic analysis (Worthy & Boles 2011).
- *?Aptornis proasciarostratus* Worthy *et al*., 2011, thoracic vertebrae (holotype NMNZ S.52350 and paratype NMNZ S.52353) from the Bannockburn Formation (early Miocene, Altonian, 18.26–15.97 Ma) of Otago, New Zealand. Although the Miocene fossils are fragmentary, the analysis of Quaternary fossils indicated that *Aptornis* belongs in crown Gruiformes, either in the Ralloidea (Boast et al. 2019) or sister to Psophiidae (Musser & Cracraft 2019).
- *Gallirallus* sp., *Porzana* sp. from the Late Pleistocene (0.129–0.0117 Ma) at Barra do Antonião Piauí, Brazil (Guérin et al. 1996).
- *Gallinula chloropus*, *Porphyrio porphyrio*, *Hovacrex roberti* from Antsirabe (Holocene, 0.002–0 Ma), Madagascar (Goodman 1999).

**Fossil record remarks:**

**Clade age estimation:**

One-sample Kolmogorov-Smirnov test

data: Mages

D = 0.24827, p-value = 0.6216

alternative hypothesis: two-sided

Quantiles:

0% 50% 95% 97.5%

55.63 60.76 80.40 86.71

Parameters of the skewStudent function:

offset xi omega alpha nu

55.629 55.680 6.745 160.041 2.502

**
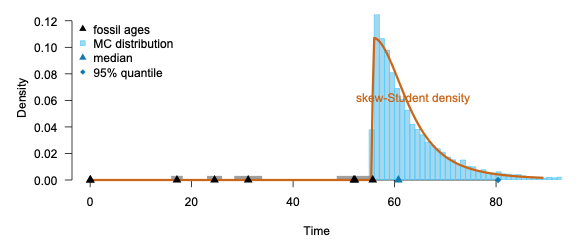
**

**References:**

Bertelli, S., L. M. Chiappe & G. Mayr. 2011. A new Messel rail from the Early Eocene Fur Formation of Denmark (Aves, Messelornithidae). Journal of Systematic Palaeontology 9.4: 551-562. Musser, G., Ksepka, D.T. and Field, D.J., 2019. New material of Paleocene-Eocene Pellornis (Aves: Gruiformes) clarifies the pattern and timing of the extant gruiform radiation. Diversity, 11(7):1-25.

Boast, A.P., Chapman, B., Herrera, M.B., Worthy, T.H., Scofield, R.P., Tennyson, A.J., Houde, P., Bunce, M., Cooper, A. and Mitchell, K.J., 2019. Mitochondrial Genomes from New Zealand’s Extinct Adzebills (Aves: Aptornithidae: Aptornis) Support a Sister-Taxon Relationship with the Afro-Madagascan Sarothruridae. Diversity, 11(2), p.24.

Boles, W.E., 2005. A new flightless gallinule (Aves: Rallidae: Gallinula) from the Oligo-Miocene of Riversleigh, northwestern Queensland, Australia. Records of the Australian Museum *57*(2), p.179.

Goodman, S.M. 1999. Holocene bird subfossils from the sites of Ampasambazimba, Antsirabe and Ampoza, Madagascar:Changes in the avifauna of south central Madagascar over the past few millennia. In: Adams, N.J. & Slotow, R.H. (eds) Proc. 22 Int. Ornithol. Congr., Durban: 3071-3083. Johannesburg: BirdLife South Africa.

Guérin, C, M. A. Curvello, M. Faure, M. Hugueney & C. Mourer-Chauviré 1996 The Pleistocene fauna of Piauí (Northeastern Brazil): paleochronological and biochronological implications. Proceedings of the International Meeting on the Peopling of the Americas, São Raimundo Nonato, Piauí, Brasil (1993). Fundamentos 1(1):55-103.

Hesse, A. 1992. A new species of Messelornis (Aves: Gruiformes: Messelornithidae) from the Middle Eocene Green River Formation. Papers in Avian Paleontology honoring Pierce Brodkorb.-Los Angeles County Museum of Natural History, Science Series, 36, 171-178.

Hoch, E. 1997. Notes on palaeornithology and on a New Bird from the Early Tertiary North Sea Region. Geological Society of Denmark, Online Series 1.

Hou, L.H. 1990 An Eocene bird from Songzi, Hubei province. Vertebr. Palasiat. 28:34-42.

Musser G., D. T. Ksepka & D. J. Field 2019. New Material of Paleocene-Eocene *Pellornis* (Aves: Gruiformes) Clarifies the Pattern and Timing of the Extant Gruiform Radiation. Diversity 11(7):1-25. <https://doi.org/10.3390/d11070102>

Musser, G.M. & J. Cracraft 2019. A new morphological dataset reveals a novel relationship for the adzebills of New Zealand (*Aptornis*) and provides a foundation for total evidence neoavian phylogenetics. American Museum Novitates 2019(3927):1-70.

Musser, G. & J. A. Clarke 2020. An exceptionally preserved specimen from the Green River Formation elucidates complex phenotypic evolution in Gruiformes and Charadriiformes. Frontiers in Ecology and Evolution 8(326):1-18.

Rasmussen, D. T., Olson, S. L. & E. L. Simons. 1987. Fossil birds from the Oligocene Jebel Qatrani Formation, Fayum Province, Egypt. Smithsonian Contributions to Paleobiology 62:1-20. Seiffert, E. R. 2006. Revised age estimates for the later Paleogene mammal faunas of Egypt and Oman. Proceedings of the National Academy of Sciences of the United States of America 103:5000-5005.

Stokke, E. W., E. Liu & M. T. Jones 2020a. Evidence of explosive hydromagmatic eruptions during the emplacement of the North Atlantic Igneous Province. Volcanica 3(2):227-250.

Stokke, E. W., M. T. Jones, J. E. Tierney, H. H. Svensen, & J. H. Whiteside 2020b. Temperature changes across the Paleocene-Eocene Thermal Maximum – a new high-resolution TEX86 temperature record from the Eastern North Sea Basin. Earth and Planetary Science Letters 544:116388. doi: 10.1016/j.epsl. 2020.116388.

Wang, Min, Gerald Mayr, Jiangyong Zhang, and Zhonghe Zhou. 2012. Two new skeletons of the enigmatic, rail-like avian taxon Songzia Hou, 1990 (Songziidae) from the early Eocene of China. Alcheringa: An Australasian Journal of Palaeontology 36(4): 487-499.

Weidig, I. 2010. New Birds from the Lower Eocene Green River Formation, North America. Records of the Australian Museum 62: 29–44.

Wang, M., Mayr, G., Zhang, J. and Zhou, Z., 2012. Two new skeletons of the enigmatic, rail-like avian taxon Songzia Hou, 1990 (Songziidae) from the early Eocene of China. Alcheringa, 36(4), pp.487-499.

Weidig, I., 2010. New birds from the lower Eocene Green River Formation, North America. *Records of the Australian Museum*, *62*(1), pp.29-44.

Worthy, T. H., & Boles, W. E. (2011). *Australlus*, a new genus for *Gallinula disneyi* (Aves: Rallidae) and a description of a new species from Oligo-Miocene deposits at Riversleigh, northwestern Queensland, Australia. Records of the Australian Museum, 63(1), 61-77.

Worthy, T.H.; Tennyson, A.J.; Scofield, R.P. Fossils reveal an early Miocene presence of the aberrant gruiform Aves: Aptornithidae in New Zealand. J. Ornithol. 2011, 152, 669–680.

**Calibration: crown Jacanida**

**Category: B**

**MRCA of:**  Jacanidae, Rostratulidae, Pedionomidae and Thinocoridae

**Clade definition in tree:** *Jacana jacana, Thinocorus orbignyianus*

**Oldest fossil:** *Nupharanassa bulotorum* Rasmussen *et al.*, 1987, distal end of right tarsometatarsus (holotype DPC 3848) from the upper sequence of the Jebel Qatrani Formation at Quarry M (30.2–29.5 Ma), Fayum Province, Egypt.

**Phylogenetic placement justification:** *Nupharanassa bulotorum* was included in a combined osteological and molecular phylogenetic analysis of Charadriiformes and found to form a clade with *Hydrophasianus* (the representative extant Jacanidae) (Smith & Clarke 2015). Although samples of Rostratulidae, Pedionomidae, and Thinocoridae, the sister families of Jacanidae were not included in the analysis, *Nupharanassa* shows a synapomorphy of Jacanidae not observed in Rostratulidae—a large distal vascular foramen—that would support its affinities with Jacanidae, as originally hypothesized by Rasmussen *et al.* (1987). Therefore, *Nupharanassa bulotorum* is used to constrain the minimum age of crown Jacanida.

**Minimum age:** 29.5 Ma

**Minimum age justification:** The youngest Fayum quarries, I and M, are estimated to be between 30.2 and 29.5 based on magnetostratigraphic correlations (Seiffert 2006).

**First occurrences:**

- *Nupharanassa bulotorum* Rasmussen *et al*., 1987 distal tarsometatarsi the Jebel Qatrani Formation at Quarry M (late Oligocene, 30.2–29.5 Ma, Seiffert 2006), Fayum Province, Egypt.
- *Oligonomus milleri* De Pietri *et al.*, 2015, left coracoid from member 7 of the Etadunna Formation (Faunal Zone B) (late Oligocene, 25.2–24.9 Ma, Megirian *et al.* 2010) at Steve's Site, Lake Palankarinna, South Australia, Australia. Affinities with Pedionomidae based on combination of similarities.
- *Hakawai melvillei* De Pietri *et al.*, 2016, several bones from Bed HH4 of the Manuherikia River near St Bathans (early Miocene, Altonian, 18.26–15.97 Ma), Otago, South Island, New Zealand. Derived characters suggest affinities with Pedionomidae (De Pietri *et al.* 2016).
- *Rostratula pulia* Mlíkovský, 1998 distal right tarsometatarsus from the early Miocene (MN4, 17.2–16.4 Ma) at Dolnice, West Bohemia, Czech Republic.
- Thinocoridae indeterminate, distal tarsometatarsus (INGEO-PV 032) from La Colmena Member of the Loma de Las Tapias Formation (late Miocene, Chasicoan, 10–9 Ma) at Loma de Las Tapias, San Juan province, Argentina (Agnolin *et al.* 2015). Affinities based on inferred derived characters (Agnolin *et al.* 2015).
- *Jacana farrandi* Olson, 1976, distal tarsometatarsus from the Alachua Formation at “Rhino Hole” McGehee Farm (late Miocene, Hemphillian 1, 9–7.5 Ma), Florida, USA. The morphology matches very closely the distinctive morphology of Jacana (Olson 1976).

**Fossil record remarks:**

**Clade age estimation:**

One-sample Kolmogorov-Smirnov test

data: Mages

D = 0.18859, p-value = 0.9553

alternative hypothesis: two-sided

Quantiles:

0% 50% 95% 97.5%

29.51 32.47 43.88 48.13

Parameters of the skewStudent function:

offset xi omega alpha nu

29.513 29.710 3.625 31.595 2.299

**
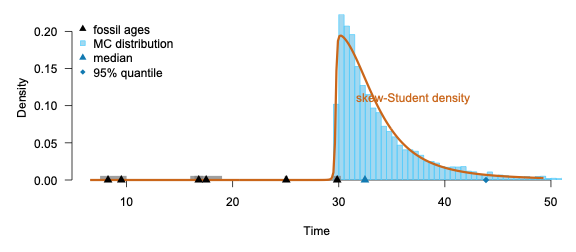
**

**References:**

Agnolín, F. L., Tomassini, R. L., & Contreras, V. H. (2015, December). Oldest record of Thinocoridae (Aves, Charadriiformes) from South America. In Annales de Paléontologie. Elsevier Masson.

De Pietri, V.L., Camens, A.B. and Worthy, T.H., 2015. A Plains‐wanderer (Pedionomidae) that did not wander plains: a new species from the Oligocene of South Australia. Ibis, 157(1), pp.68-74.

De Pietri, V. L., R. P. Scofield, A. J. D. Tennyson, S. J. Hand & T. H. Worthy. 2016. Wading a lost southern connection: Miocene fossils from New Zealand reveal a new lineage of shorebirds (Charadriiformes) linking Gondwanan avifaunas. Journal of Systematic Palaeontology: 1-14.

Megirian, D., Prideaux, G. J., Murray, P. F., & Smit, N. 2010. An Australian land mammal age biochronological scheme. Paleobiology, 36(4), 658-671.

Mlíkovský J. 1998 A new painted snipe (Aves: Rostratulidae) from the early Miocene of the Czech Republic. Casopis Národního muzea Rada Prírodovedná 167:99-101.

Olson, S. 1976 A jacana from the Pliocene of Florida (Aves: Jacanidae). Proceedings of the Biological Society of Washington 89(19):259–264.

Rasmussen, D. T., Olson, S. L. & E. L. Simons. 1987. Fossil birds from the Oligocene Jebel Qatrani Formation, Fayum Province, Egypt. Smithsonian Contributions to Paleobiology 62:1-20.

Seiffert, E. R. 2006. Revised age estimates for the later Paleogene mammal faunas of Egypt and Oman. Proceedings of the National Academy of Sciences of the United States of America 103:5000-5005.

Smith, N. A., & J. A. Clarke 2015. Systematics and evolution of the Pan‐Alcidae (Aves, Charadriiformes). Journal of Avian Biology 46(2):125-140.

**Calibration: crown Alcini**

**Category: A**

**MRCA of: *Alca* and *Uria***

**Clade definition in tree:** *Alca torda, Uria aalge*

**Oldest fossil:** *Miocepphus bohaski* Wijnker & Olson, 2009, distal humerus (paratype USNM 237142) from the lower Calvert Formation, Popes Creek Sand Member, Hanover County, Virginia, USA.

**Phylogenetic placement justification:** A combined analysis of osteological and molecular data found *Miocepphus*, including *Miocepphus bohaski*, as sister to the Little Auk *Alle alle* (Smith & Clarke 2015), thus nested in the Alcini. The holotype specimen is more complete (a partial postcranial skeleton, USNM 237270) but it is younger (Smith 2015).

**Minimum age:** 15.97 Ma

**Minimum age justification:** Biostratigraphic correlations indicate a Burdigalian age for the lower Calvert Formation in Hanover County (de Verteuil & Norris 1996), therefore the bounds of the Burdigalian (20.44–15.97 Ma) are adopted here (see Smith 2015).

**First occurrences:**

- *Miocepphus bohaski* Wijnker & Olson, 2009, distal humerus (paratype USNM 237142) from the lower Calvert Formation (Burdigalian, 20.44–15.97 Ma), Popes Creek Sand Member, Hanover County, Virginia, USA.
- *Uria brodkorbi* Howard, 1981, associated partial skeleton impressions (UF-PB 7960) from the Monterey Formation (Tortonian, 11.6–7.2 Ma) at Lompoc Quarry, Santa Barbara County, California, USA. Cladistic analysis confirmed original generic assignment (Smith & Clarke 2015).
- *Alca stewarti* Martin et al., 2000, left ulna (holotype BMNH A 7050) from the Kattendijk Formation (early Pliocene, 5.3–3.6 Ma) at Kallo, Beveren, Belgium. Cladistic analysis confirmed original generic assignment (Smith & Clarke 2015).
- *Uria onoi* Watanabe, Matsuoka & Hasegawa, 2016, a right humerus (holotype NSMT-PV 23722) from Shiriya, Locality 2 (Pleistocene, MIS9, 0.32 Ma), Japan.

**Clade age estimation:** Fossils were chosen from four geographic regions: E Pacific (California, USA), W Pacific (Japan), E Atlantic (Belgium), and W Atlantic (Virginia, USA).

One-sample Kolmogorov-Smirnov test

data: Mages

D = 0.43717, p-value = 0.3268

alternative hypothesis: two-sided

Quantiles:

0% 50% 95% 97.5%

16.03 21.70 38.48 45.73

Parameters of the skewStudent function:

offset xi omega alpha nu

16.032 17.415 5.605 6.973 2.520


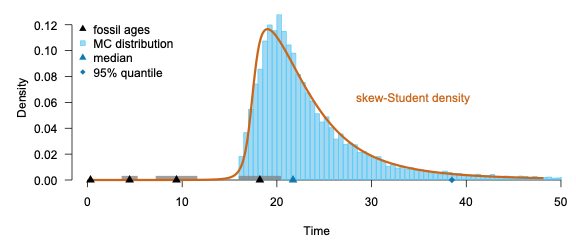


**References:**

de Verteuil, L. and Norris, G. 1996. Dinoflagellate cyst zonation and allostratigraphy of the Chesapeake Group, p. 1-82. In de Verteuil, L. and Norris, G. (eds.), Miocene Dinoflagellate Stratigraphy and Systematics of Maryland and Virginia. Micropaleontology, 42 (supplement). American Museum of Natural History, New York.

Howard. 1981. A new species of murre, genus Uria, from the Late Miocene of California (Aves: Alcidae). Bulletin of the Southern California Academy of Sciences 80(1):1-12.

Martin, J. W. R., C. A. Walker, H. C. Bonser and G. J. Dyke. 2000. A new species of large auk from the Pliocene of Belgium. Oryctos 3:53-60.

Smith, N. A. 2015. Sixteen vetted fossil calibrations for divergence dating of Charadriiformes (Aves, Neognathae). Palaeontologia Electronica 18:1.4FC:1-18. <https://doi.org/10.26879/410>

Smith, N. A., & J. A. Clarke 2015. Systematics and evolution of the Pan‐Alcidae (Aves, Charadriiformes). Journal of Avian Biology 46(2):125-140.

Watanabe, J., H. Matsuoka, and Y. Hasegawa. 2016. Two species of *Uria* (Aves: Alcidae) from the Pleistocene of Shiriya, northeast Japan, with description and body mass estimation of a new species. Bulletin of the Gunma Museum of Natural History 20:59-72

Wijnker, E. & Olson, S.L. 2009. A revision of the fossil genus *Miocepphus* and other Miocene Alcidae (Aves: Charadriiformes) of the western north Atlantic Ocean. Journal of Systematic Palaeontology 7:471-487.

##

## AEQUORNITHES

**Calibration: crown Phaethontimorphae**

**Category: A**

**MRCA of:** Phaethontiformes and Eurypygiformes

**Clade definition in tree:** *Phaethon lepturus, Eurypyga helias*

**Oldest fossil:** *Lithoptila abounensis* Bourdon, 2005, neurocranium (holotype CP.DEK/GE 1087) from the bed IIa of the Ouled Abdoun Phosphates Series (Thanetian), Ouled Abdoun Basin, Morocco (Bourdon *et al.* 2005).

**Phylogenetic placement justification:** A cladistic analysis using 47 osteological characters and 17 terminals found *L. abounensis* sister to *Prophaethon*, forming a stem clade of Phaethontidae (Bourdon *et al.* 2005, see also Smith 2010). However, the encompassing clade Phaethontiformes was sister to the Procellariiformes and no Eurypygiformes was included.

**Minimum age:** 55.8 Ma

**Minimum age justification: “**The specimen is dated as Thanetian (bed IIa of the mining lithostratigraphical terminology, Fig. 2) on the basis of the selachians identified in the matrix…” (Bourdon *et al.* 2005), whose upper boundary is 55.8 Ma.

**First occurrences:**

- *Lithoptila abounensis* Bourdon, 2005, neurocranium (holotype) from the bed IIa of the Ouled Abdoun Phosphates Series (Thanetian, 58.7–55.8 Ma), Ouled Abdoun Basin, Morocco (Bourdon *et al.* 2005).
- *Zhylgaia aestiflua* Nessov, 1988, two partial humeri from the late Paleocene (58.7–55.8 Ma) of Kazakhstan. Bourdon *et al.* (2008) proposed placement in Prophaethontiformes. *Tshulia litorea* Nessov, 1988, based on a tarsometatarsus from the same locality, represents the same taxon according to Mayr & Scofield (2016).
- *Prophaethon* sp., distal humerus (USNM 483158) from the Aquia Formation (Thanetian, 58.7–55.8 Ma) at Capital Beltway and Central Avenue, Prince George’s County, Maryland, USA (Olson 1994).
- *Prophaethon shrubsolei* Andrews, 1899 from the Walton Member of the London Clay Formation at Walton-on-the-Naze (54–54.9 Ma, King 2016), Essex, United Kingdom (Mayr 2015).

**Fossil record remarks:**

**Clade age estimation:** Quaternary record not used to preserve uniformity.

One-sample Kolmogorov-Smirnov test

data: Mages

D = 0.53326, p-value = 0.1384

alternative hypothesis: two-sided

Quantiles:

0% 50% 95% 97.5%

55.95 58.78 62.31 63.71

Parameters of the skewStudent function:

offset xi omega alpha nu

55.952 57.879 1.273 1.892 2.901


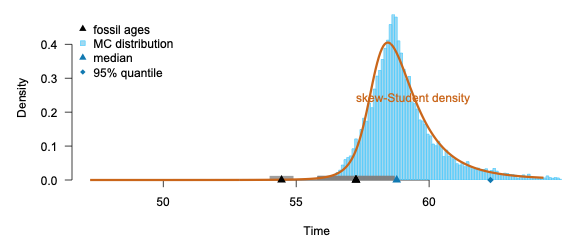


**References:**

Andrews C. W. 1899. On the remains of a new bird from the London Clay of Sheppey. Proceedings of the Zoological Society of London 1899:776-785.

Bourdon, E., B. Bouya & M. Iarchene 2005. Earliest African Neornithine Bird: A New Species of Prophaethontidae (Aves) from the Paleocene of Morocco. J. Vert. Paleontol. 25:157-170.

Bourdon, E., C. Mourer-Chauviré, M. Amaghzaz & Bouya 2008. New specimens of *Lithoptila abdounensis* (Aves, Prophaethontidae) from the lower Paleogene of Morocco. J. Vert. Paleontol. 28: 751-761.

Collinson, M. E., Adams, N. F., Manchester, S. R., Stull, G. W., Herrera, F., Smith, S. Y., Andrew, M. J., Kenrick, P. & Sykes, D. 2016. X-ray micro-computed tomography (micro-CT) of pyrite-permineralized fruits and seeds from the London Clay Formation (Ypresian) conserved in silicone oil: a critical evaluation. Botany 94:697–711. doi:10.1139/cjb-2016-0078

King, C. 2016. A revised correlation of Tertiary rocks in the British Isles and adjacent areas of NW Europe. Geol. Soc. Lond. Spec. Rep. 27: 1–719.

Mayr, G. & R. P. Scofield. 2016. New avian remains from the Paleocene of New Zealand: the first early Cenozoic Phaethontiformes (tropicbirds) from the Southern Hemisphere. J. Vert. Paleontol.: e1031343.

Mayr, G. 2015. New remains of the Eocene *Prophaethon* and the early evolution of tropicbirds (Phaethontiformes). Ibis 157(1):54-67.

Nessov, L. A. 1988. [New Cretaceous and Paleocene birds of Soviet Mid- dle Asia and Kazakhstan and their environments]. Trudy Zoologiceskogo Instituta Akademii Nauk SSSR 182:116–123. [Russian]

Olson, S. L. 1994. A giant *Presbyornis* (Aves, Anseriformes) and other birds from the Paleocene Aquia Formation of Maryland and Virginia. Proceedings of the Biological Society of Washington 107:429-435.

Smith, N. D. 2010 Phylogenetic analysis of Pelecaniformes (Aves) based on osteological data: implications for waterbid phylogeny and fossil calibration studies. PLoS One 5:e13354.

**Calibration: crown Procellariimorphae**

**Category: A**

**MRCA of: Sphenisciformes and Procellariiformes**

**Clade definition in tree:** *Pygoscelis adeliae, Fulmarus glacialis*

**Oldest fossil:** *Waimanu manneringi* Jones, Ando & Fordyce, 2006, and associated partial postcranial skeleton (holotype CM zfa35) from the Waipara Greensand Formation, Waipara River, Canterbury, New Zealand (Slack *et al.* 2006)

**Phylogenetic placement justification:** The affinities of *Waimanu manneringi* as a stem Sphenisciformes are well established based on inferred apomorphies and results of several parsimony and Bayesian phylogenetic analyses (Slack *et al.*, 2006, Ksepka et al. 2006, Ksepka et al. 2012, Blokland et al. 2019)

**Minimum age:** 60.5 Ma

**Minimum age justification:** Dated by nannofossil biostratigraphy. The matrix of the holotype CM zfa35 contained the coccolithophores *Chiasmolithus bidens* and *Hornibrookina teurensis* which indicate an age between 61.6 and 60.5 Ma (Slack *et al.* 2006). *Waimanu manneringi* is nearly coeval with several other fossil penguins but its age is better constrained, and its minimum possible age, in particular, is older than the others (Blokland et al. 2019).

**First occurrences:**

- *Waimanu manneringi* Jones, Ando & Fordyce 2006, and associated partial postcranial skeleton (CM zfa35) from the Waipara Greensand (Paleocene, 61.6–60.5 Ma), Waipara River, Canterbury, New Zealand (Slack *et al.* 2006).
- *Crossvallia unienwillia* Tambussi *et al.*, 2005, humerus, femur, partial tibiotarsus, thoracic vertebra (holotype Museo de La Plata 00-I-10-1) from the Bahía Pingüino Member of the Cross Valley Formation at Seymour Island (Late Paleocene, 56–55 Ma).
- *Perudyptes devriesi* Clarke *et al.*, 2007, partial skeleton (MUSM 889) from the Paracas Formation (Middle Eocene, 42 – 42 Ma) at Quebrada Perdida, Peru.
- "*Pachydyptes*" *simpsoni* Jenkins, 1974, a partial skeleton (SAMA P14157) from the Blanche Point Formation (Late Eocene, 38.0–36.5 Ma, Park & Fitzgerald 2012) Australia. Phylogenetically placed by Ksepka *et al.* (2006) and Ksepka & Clarke (2012).
- *Makahala mirae* Mayr, 2015 (Procellariiformes), wing bones (SMF Av 603) from Jansen Creek Member of the Makah Formation (Rupelian, 33.9–28.4 Ma), near the mouth of Bullman Creek, Clallam County, Washington, USA.
- *Rupelornis definitus* van Beneden, 1871, (Diomedeoididae, De Pietri et al. 2009, Mayr & Smith 2012) from Frauenweiler (Rupelian, 32–30.5 Ma, Maxwell *et al.* 2016), Germany. Diomedeoididae most probably a stem group Procellariiformes (De Pietri et al. 2009, Mayr & Smith 2012).
- *Inguza predemersus* (Simpson, 1971), *Dege hendeyi* Simpson, 1979, *Nucleornis insolitus* Simpson, 1979 and *Palaeospheniscus*? *huxleyorum* Simpson, 1973 from the Varswater Formation at Saldanha Steel (late Miocene, 11.6–7.2 Ma), South Africa (Thomas & Ksepka 2013).
- *Phoebastria* cf. *albatrus*, a right humeral shaft (KUGM FA 2018.007) from the Hirayama Formation (early Pleistocene, 1.7 Ma), Japan (Watanabe *et al.* 2019).
- *Puffinus* *pacificus* (Gmelin, 1789) from Holocene deposits (1755 - 365 a) in Marais de l'Ermitage, Reunión Island, Mascarene Islands (Mourer-Chauviré *et al.* 1999).

**Fossil record remarks:**

**Clade age estimation:**

One-sample Kolmogorov-Smirnov test

data: Mages

D = 0.1971, p-value = 0.8121

alternative hypothesis: two-sided

Quantiles:

0% 50% 95% 97.5%

60.53 65.85 84.70 91.09

Parameters of the skewStudent function:

offset xi omega alpha nu

60.530 60.845 6.749 37.833 2.655

**
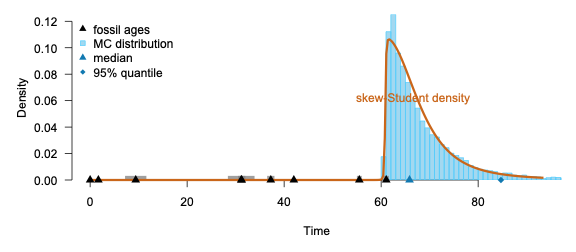
**

**References:**

Blockland, J. C., C. M. Reid, T. H. Worthy, A. J. D. Tennyson, J. A. Clarke, & R. P. Scofield. 2019. Chatham Island Paleocene fossils provide insight into the palaeobiology, evolution, and diversity of early penguins (Aves, Sphenisciformes). Palaeontologia Electronica 22 (3):78.

Clarke, J. A., D. T. Ksepka, M. Stucchi, M. Urbina, N. Giannini, S. Bertelli, Y. Narváez & C. A. Boyd. 2007. Paleogene equatorial penguins challenge the proposed relationship between biogeography, diversity, and Cenozoic climate change. Proceedings of the National Academy of Sciences 104:11545-11550.

Jenkins 1974 A new giant penguin from the Eocene of Autralia. Paleontology 17:291-310.

Ksepka, D. T., & J. A. Clarke. 2012. The basal penguin (Aves: Sphenisciformes) *Perudyptes devriesi* and a phylogenetic evaluation of the penguin fossil record. Bulletin of the American Museum of Natural History 337:1-77.

Ksepka, D. T., S. Bertelli & N. P. Giannini. 2006. The phylogeny of the living and fossil Sphenisciformes (penguins). Cladistics 22(5): 412-441.

Mayr, G. 2015. A new Paleogene procellariiform bird from western North America. Neues Jahrbuch für Geologie und Paläontologie-Abhandlungen 275(1):11-17.

Mayr, G., & T. Smith .2012. Phylogenetic affinities and taxonomy of the Oligocene Diomedeoididae, and the basal divergences amongst extant procellariiform birds. Zoological Journal of the Linnean Society, 166:854-875.

Mourer-Chauviré, C., R. Bour, S. Ribes & F. Moutou 1999. The avifauna of Réunion Island (Mascarene Islands) at the time of the arrival of the first Europeans. Pp. 1-38 in S. Olson (ed) Avian paleontology at the close of the 20th century. Smithsonian Contributions to Paleontology 89.

Park, T., & E. M. G. Fitzgerald 2012. A review of Australian fossil penguins (Aves: Sphenisciformes). Memoirs of Museum Victoria 69:309-325.

Slack, K. E., C. M. Jones, T. Ando, G. L. Harrison, R. E. Fordyce, U. Arnason & D. Penny 2006. Early penguin fossils, plus mitochondrial genomes, calibrate avian evolution. Mol. Biol. Evol. 23 (6):1144-1155.

Tambussi, C. P., M. A. Reguero, S. A. Marenssi, & S. N. Santillana 2005. Crossvallia unienwillia, a new Spheniscidae (Sphenisciformes, Aves) from the Late Paleocene of Antarctica. Geobios 38:667-675.

Thomas, D. B., & D. T. Ksepka. 2013. A history of shifting fortunes for African penguins. Zoological Journal of the Linnean Society 168:207-2019

Watanabe, J., Koizumi, A., Nakagawa, R., Takahashi, K., Tanaka, T. and Matsuoka, H., 2019. Seabirds (Aves) from the Pleistocene Kazusa and Shimosa Groups, Central Japan. Journal of Vertebrate Paleontology,39(5):e1697277.

**Calibration: crown Spheniscidae**

**Category:** B (nested within calibration node)

**MRCA of:** *Pygoscelis adeliae, Aptenodytes forsteri*

**Clade definition in tree:**

**Oldest fossil:** *Madrynornis mirandus* Acosta Hospitaleche, Tambussi, Donato & Cozzuol, 2007**,** a nearly complete and articulated skeleton (holotype MEF− PV 100) from the lower levels of the Puerto Madryn Formation at the southern coast of Golfo San José (Playa Villarino), Península Valdés, Chubut prov., Argentina (Acosta Hospitaleche et al. 2007).

**Phylogenetic placement justification:** The original cladistic analysis of osteological characters placed *Madrynornis* as sister to *Eudyptes* (Acosta Hospitaleche *et al.* 2007) but in a tree that was inconsistent with most other assessment of penguin phylogeny and subsequent analyses indicated alternative placements of *Madrynornis*, including a position outside crown Spheniscidae (see Degrange *et al.* 2017 for a review). Degrange *et al.* (2017) reexamined the specimen of *Madrynornis* and included it in a new cladistic analysis combining osteological and molecular characters and including numerous other fossil penguins. They found *Madrynornis* sister to the *Eudyptula*-*Spheniscus* clade, a position supported by derived features of the cranium. More recent calibrated Bayesian analyses placed *Madrynornis* as a direct ancestor (Gavryushkina *et al.* 2017) or sister (Thomas *et al.* 2020) to the *Megadyptes*-*Eudyptes* clade. Despite the discrepancy, both analyses placed *Madrynornis* as a basal taxon in the broader *Eudyptula*-*Spheniscus + Megadyptes*-*Eudyptes* clade and therefore it sets a minimum age for this clade and crown Spheniscidae.

**Minimum age:** 9 Ma

**Minimum age justification:** The Puerto Madryn Formation has been dated as between 11.9 and 9 Ma based on radiometric dating analyses (del Rio *et al.* 2018).

**First occurrences:**

- *Madrynornis mirandus* Acosta Hospitaleche, Tambussi, Donato & Cozzuol, 2007**,** a nearly complete and articulated skeleton (holotype MEF− PV 100) from the lower levels of the Puerto Madryn Formation (late Miocene, 11.9–9 Ma, del Rio *et al.* 2018) at the southern coast of Golfo San José (Playa Villarino), Península Valdés, Chubut prov., Argentina.
- *Pygoscelis tyreei* Simpson, 1972, partial articulated skeleton (holotype CM AV 16527) from Motunau Beach, North Canterbury, New Zealand. Stem *Pygoscelis* according to Bayesian time-calibrated tree (Thomas et al. 2020). Age poorly constrained but likely Waiauan-Tongaporutuan (11.5–6 Ma, Feldmann & Keyes 1992).
- *Spheniscus* sp., almost complete right humerus (IB/P/B-0965) and complete left radius (IB/P/B-0966) from the Fisher Bench Formation at Prince Charles Mountains, Fisher Massif (10.2 Ma based on Strontium Isotope Stratigraphy), Mac Robertson Land, East Antarctica (Jadwiszczak *et al.* 2013).
- *Nucleornis insolitus* Simpson 1979 from the Varswater Formation at the Koeberg Nuclear Power Station (Zanclean, 5.3–3.6 Ma). South Africa.
- *Eudyptes pachyrhynchus* (ANWC B21141), subfossil pelvis from Stockyard Site (late Holocene 760 ± 70 a), Hunter Island, Tasmania, Australia (van Tets et al. 1983, Worthy & Nguyen 2020).

**Fossil record remarks:**

**Clade age estimation:**

One-sample Kolmogorov-Smirnov test

data: Mages

D = 0.1994, p-value = 0.9627

alternative hypothesis: two-sided

Quantiles:

0% 50% 95% 97.5%

10.20 12.50 19.67 22.89

Parameters of the skewStudent function:

offset xi omega alpha nu

10.201 10.210 3.043 215.169 3.207

**
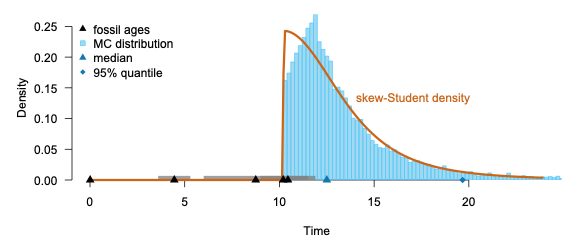
**

**References:**

Acosta Hospitaleche, C., C. Tambussi, M. Donato & M. Cozzuol. 2007. A new Miocene penguin from Patagonia and its phylogenetic relationships. Acta Palaeontologica Polonica 52:299–314.

Degrange, F. J., D. T. Ksepka & C. P. Tambussi 2018 Redescription of the oldest crown clade penguin: cranial osteology, jaw myology, neuroanatomy, and phylogenetic affinities of *Madrynornis mirandus*. J. Vertebr. Paleontol. 38:e1445636. doi:10.1080/02724634.2018.1445636

del Río, C. J., S. A. Martínez, J. M. McArthur, M. F. Thirlwall & L. M. Pérez 2018. Dating late Miocene marine incursions across Argentina and Uruguay with Sr-isotope stratigraphy. Journal of South American Earth Sciences 85: 312–324.

Gavryushkina, A., T.A. Heath, D. T. Ksepka, T. Stadler, D. Welch & A.J. Drummond 2017. Bayesian total-evidence dating reveals the recent crown radiation of penguins. Systematic Biology 66(1):57-73.

Jadwiszczak, P., K. P. Krajewski, Z. Pushina, A. Tatur, and G. Zielinski. 2013. The first record of fossil penguins from East Antarctica. Antarctic Science 25(3):397-408

Simpson, G. G., 1972. Pliocene penguins from North Canterbury, New Zealand. Records of the Canterbury Museum 9(2):159-182.

Simpson, G. G. 1979. Tertiary penguins from the Duinefontein site, Cape Province, South Africa. Annals of the South African Museum 79(1):1-7

Thomas, D. B., A. J. D. Tennyson, R. P. Scofield, T. A. Heath, W. Pett & D. T. Ksepka. 2020. Ancient crested penguin constrains timing of recruitment into seabird hotspot. Proceedings of the Royal Society B 287(1932): 20201497.

van Tets, G.F., and O’Connor, S. 1983. The Hunter Island penguin, an extinct new genus and species from a Tasmanian midden. Records of the Queen Victoria Museum 81: 1–13.

Worthy, T. H. & J. M. T. Nguyen 2020. An annotated checklist of the fossil birds of Australia. Transactions of the Royal Society of South Australia 144 (1):66-108.

**Calibration: crown Procellariiformes**

**Category: B**

**MRCA of:** Diomedeidae and Procellariidae

**Clade definition in tree:** *Thalassarche chlororhynchos, Fulmarus glacialis*

**Oldest fossil:** *Notoleptos giglii* Acosta Hospitaleche & Gelfo 2017, left tarsometatarsus in two fragments (holotype MLP 12-I-20-305) from Submeseta III Allomember (37.2–33.9 Ma), La Meseta Formation, Locality DPV 16/84, Seymour Island, West Antarctica.

**Phylogenetic placement justification:** A cladistic analysis of the holotype indicated closer affinities with *Diomedea* than with *Puffinus*, *Oceanites*, *Pachyptila* or *Pelecanoides*, although with minimal support (bootstrap support < 50%, Bremer support 1, Acosta Hospitaleche & Gelfo 2017) and the topology among extant taxa (*e.g.* Dioemedeidae nested within Procellariidae) is inconsistent with current estimates.

**Minimum age:** 33.9 Ma

**Minimum age justification:** Fossiliferous strata at Locality DPV 16/84 correspond to the upper Submeseta III Allomember that has been determined as Priabonian in age (Acosta Hospitaleche & Gelfo 2017), therefore, the bounds of the Priabonian are adopted here as age bounds for the holotype.

**First occurrences:**

- *Notoleptos giglii* Acosta Hospitaleche & Gelfo, 2017 (Diomedeidae), left tarsometatarsus in two fragments (MLP 12-I-20-305) from Submeseta III Allomember (37.2–33.9 Ma), La Meseta Formation, Locality DPV 16/84, Seymour Island, West Antarctica.
- *Tydea septentrionalis* Mayr & Smith, 2012 (Diomedeidae), multiple bones from the wings and shoulder girdle, eroded, cracked and fragmented that show derived traits of Diomedeidae from the Boom Formation (Rupelian, 33.9–28.4 Ma) at Terhagen, Belgium.
- *Makahala mirae* Mayr, 2015 (Procellariiformes), associated wing bones (SMF Av 603) from the Jansen Creek Member of the Makha Formation at Bullman Creek (Rupelian, 33.9–28.4 Ma), Clallam County, Washington State, USA.
- *Pelecanoides miokuaka* Worthy *et al.*, 2007 (Pelecanoididae) complete right humerus from Bannockburn Formation, St Bathans Fauna (Altonian, 18.26–15.97 Ma), Otago, New Zealand.
- *Pachyptila* sp., incomplete skull (MPC-601) from Bahía Inglesa Formation (11.6–5.33 Ma) near Bahía Inglesa, Atacama Region, Chile (Sallaberry *et al.* 2007).
- *Diomedea thyridata* Wilkinson, 1969, incomplete premaxilla (Holotype NMV P24172) from the N17-18 zone in the Sandringham Sandstone Member of the Black Rock Sandstone Formation at Beaumaris (Messinian, 6.2–53 Ma), Victoria, Australia.
- *Oceanites zaloscarthmus* Olson, 1985 (type: L25214, complete right humerus) *Pachyptila salax* Olson, 1985 (type: L25187, complete left humerus) and *Pelecanoides cymatotrypetes* Olson, 1985 (type: Complete left humerus, L14564) from the Quartzose Sand Member of the Varswater Formation at Langebaanweg (Early Pliocene 5.33–3.6 Ma) Cape Province, South Africa.
- *Phoebastria* cf. *albatrus* right humeral shaft (KUGM FA 2018.007) from the Hirayama Formation (Pleistocene, 1.7 Ma), Japan (Watanabe *et al.* 2019)
- *Puflinus* *pacificus* (Gmelin 1789) from Holocene deposits (1755–365 a) Marais de l'Ermitage, Reunión Island, Mascarene Islands (Mourer-Chauviré *et al.* 1999).

**Fossil record remarks:**

**Clade age estimation:**

One-sample Kolmogorov-Smirnov test

data: Mages

D = 0.39365, p-value = 0.09084

alternative hypothesis: two-sided

Quantiles:

0% 50% 95% 97.5%

33.91 38.50 49.73 53.69

Parameters of the skewStudent function:

offset xi omega alpha nu

33.909 35.015 4.861 7.850 3.607

**
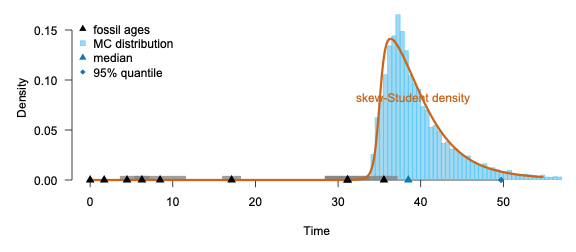
**

**References:**

Acosta Hospitaleche, C. & N. J. Gelfo 2017. Procellariiform remains and a new species from the latest Eocene of Antarctica. Historical Biology 29(6):755-769.

Mayr, G. 2015. A new Paleogene procellariiform bird from western North America. Neues Jahrbuch für Geologie und Paläontologie-Abhandlungen 275(1):11-17.

Mayr, G. & T. Smith 2012. A fossil albatross from the early Oligocene of the North Sea basin. The Auk 129(1):87−95.

Mourer-Chauviré, C., R. Bour, S. Ribes & F. Moutou 1999. The avifauna of Réunion Island (Mascarene Islands) at the time of the arrival of the first Europeans. Pp. 1-38 in S. Olson (ed) Avian paleontology at the close of the 20th century. Smithsonian Contributions to Paleontology 89.

Olson, S. L. 1985. Early Pliocene Procellariiformes (Aves) from Langebaanweg, South-Western Cape Province, South Africa. Annals of the South African Museum 95(3):123-145.

Sallaberry, M., D. Rubial-Rogers, M. E. Suárez & C. S. Gustein. 2007. The skull of a fossil Prion (Aves: Procellariiformes) from the Neogene (Late Miocene) of northern Chile. Revista Geológica de Chile 34 (1):147-154.

Watanabe, J., Koizumi, A., Nakagawa, R., Takahashi, K., Tanaka, T. and Matsuoka, H., 2019. Seabirds (Aves) from the Pleistocene Kazusa and Shimosa Groups, Central Japan. Journal of Vertebrate Paleontology,39(5):e1697277.

Wilkinson, H. E. 1969. Description of an Upper Miocene albatross from Beaumaris, Victoria, Australia, and a review of the fossil Diomedeidae. Memoirs of the National Museum of Victoria 29:41-51.

Worthy, T. H., A. J. D. Tennyson, C. Jones, J. A. McNamara, and B. J. Douglas. 2007. Miocene waterfowl and other birds from Central Otago, New Zealand. Journal of Systematic Palaeontology 5:1–39.

**Calibration: crown Fregatidae + Suloidea**

**Category: A**

**MRCA of: Fregatidae and Suloidea**

**Clade definition in tree:** *Fregata magnificens, Sula dactylatra*

**Oldest fossil:** *Limnofregata azygosternon* Olson, 1977, Nearly complete, partially flattened skeleton with feather impressions (USNM 22753) from the Fossil Butte Member of the Green River Formation at Highway 30 North, Lincoln County, Wyoming, USA.

**Phylogenetic placement justification:** *Limnofregata azygosternon* shows several derived traits of Fregatidae (Olson 1977) and has been recovered as a stem Fregatidae (Bremer support 3, Bootstrap support 95%) in a cladistic analysis of extant and fossil Pelecaniformes including 464 characters and 59 species (Smith 2010).

**Minimum age:** 51.97 Ma

**Minimum age justification:** Radiometrical ^40^Ar/^39^Ar-dating of the K-spar tuff near the top of the Fossil Butte member yielded a recalibrated date of 51.97 ± 0.16 Ma (Smith *et al.* 2008, 2010).

**First occurrences:**

- *Limnofregata azygosternon* Olson 1977 Nearly complete, partially flattened skeleton with feather impressions (USNM 22753) from the Fossil Butte Member of the Green River Formation 51.97 ± 0.16 Ma) at Highway 30 North, Lincoln County, Wyoming, USA.
- *Masillastega rectirostris* Mayr 2002 (Sulidae) a skull from Messel (48.25 - 47.41 MaMa), Germany.
- Phalacrocoracidae indeterminate, rostrum from the Jebel Qatrani Formation at Quarry M (early Oligocene, 30.2 - 29.5 Ma, Seiffert 2006) Fayum, Egypt (Rasmussen *et al.* 1987).
- *Copepteryx hexeris* Olson & Hasegawa 1996 (Plotopteridae) from the Ainoshima Formation at Ainoshima Island (Late Oligocene: 28.4 - 23.03 Ma), Japan.
- *Anhinga walterbolesi* Worthy 2012, left tarsometatarsus (Holotype NMV P166373) from the Etadunna Formation at Snake Dam (late Oligocene: 26 – 24 Ma, Woodburne *et al.* 1994), Clayton River (Lake Eyre Basin), South Australia, Australia.
- *Meganhinga chilensis* Alvarenga 1995 postcranial associated elements (holotype SGO-PV4001) from the Malla Malla Member of the Curamallin Formation (Early Miocene, 18 – 15 Ma) at Cerro Rucañanco, Araucania Region, Chile.
- *Stictocarbo punctatus* and *Leucocarbo carunculatus* multiple material of the two species from the Late Pleistocene at Cape Wanbrow (0.13 - 0.11 Ma), Otago, South Island, New Zealand (Worthy *et al*. 2003).
- *Phalacrocorax africanus* from Antsirabe (latest Pleistocene-Holocene: 0.019 - 0.001 Ma), Madagascar (Goodman 1999).

**Fossil record remarks:** A new species of *Limnofregata hutchisoni* was described from Bitter Creek 22 Level 3, Wasatch Formation, Sweet-water County, Wyoming (Holotype: UCMP 134932 right coracoid and humerus found in articulation) (Stidham 2015). The specimens are crush but show a close resemblance to L. *azygosternon* except that they are much larger. Moreover, they show putative synapomorphies of *Limnofregata*. Stratigraphic analysis suggests that the Bitter Creek fossil is older that the Green River Formation and may be 54 to 55 Ma old (Stidham 2015).

**Clade age estimation:**

One-sample Kolmogorov-Smirnov test

data: Mages

D = 0.24786, p-value = 0.6236

alternative hypothesis: two-sided

Quantiles:

0% 50% 95% 97.5%

51.97 56.61 76.07 82.90

Parameters of the skewStudent function:

offset xi omega alpha nu

51.971 51.972 6.155 514.028 2.239


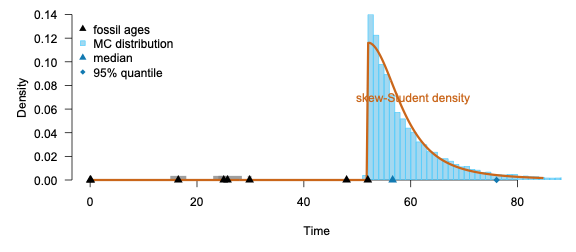


**References:**

Alvarenga, H.M.F., 1995. A large and probably flightless anhinga from the Miocene of Chile. Courier Forschungsinstitut Senckenberg 181, 149– 161.

Goodman, S.M. 1999. Holocene bird subfossils from the sites of Ampasambazimba, Antsirabe and Ampoza, Madagascar:Changes in the avifauna of south central Madagascar over the past few millennia. In: Adams, N.J. & Slotow, R.H. (eds) Proc. 22 Int. Ornithol. Congr., Durban: 3071-3083. Johannesburg: BirdLife South Africa.

Mayr, G. 2002. A skull of a new pelecaniform bird from the Middle Eocene of Messel, Germany. Acta Palaeontologica Polonica, 47: 507-512.

Olson, S. L. 1977 A Lower Eocene frigatebird from the Green River Formation of Wyoming (Pelecaniformes: Fregatidae). Smithsonian Contributions to Paleobiololgy 35: 1–33.

Olson, S. L., & Y. Hasegawa. 1996. A new genus and two new species of gigantic Plotopteridae from Japan (Aves: Pelecaniformes). Journal of Vertebrate Paleontology 16:742-751.

Rasmussen, D. T., Olson, S. L. & E. L. Simons. 1987. Fossil birds from the Oligocene Jebel Qatrani Formation, Fayum Province, Egypt. Smithsonian Contributions to Paleobiology 62:1-20.

Seiffert, E. R. 2006. Revised age estimates for the later Paleogene mammal faunas of Egypt and Oman. Proceedings of the National Academy of Sciences of the United States of America 103:5000-5005.

Smith, N. D. 2010 Phylogenetic analysis of Pelecaniformes (Aves) based on osteological data: implications for waterbid phylogeny and fossil calibration studies. PLoS One 5:e13354.

Smith, M.E., Carroll, A.R., & Singer, B.S. 2008 Synoptic reconstruction of a major ancient lake system: Eocene Green River Formation, western United States. *Geological Society of America Bulletin* 120 (1/2):54-84.

Smith, M.E., Chamberlain, K.R., Singer, B.S. & Carroll, A.R. 2010 Eocene clocks agree: coeval ^40^Ar/^39^Ar, U-Pb, and astronomical ages from the Green River Formation. Geology 38 (6):527-530.

Stidham, T.A. 2015. A new species of *Limnofregata* (Pelecaniformes: Fregatidae) from the Early Eocene Wasatch Formation of Wyoming: implications for palaeoecology and palaeobiology. *Palaeontology* 58(2):239–249.

Worthy, T. H. 2012. A New Species of Oligo-Miocene Darter (Aves: Anhingidae) from Australia. Auk 129:96-104.

Worthy, T. H. & J. A. Grant-Mackie. 2003. Late-Pleistocene avifaunas from Cape Wanbrow, Otago, South Island, New Zealand. Journal of the Royal Society of New Zealand 33(1):427-485.

**Calibration: crown Pelecanoidea**

**Category: A**

**MRCA of:** Pelecanidae, Balaenicipitidae and Scopidae

**Clade definition in tree:** *Balaeniceps_rex, Pelecanus_crispus*

**Oldest fossil:** *Pelecanus* sp. a nearly complete skull on a slab (NT-LBR-039) from Rupelian strata (33.00 - 28.25 Ma) at Pichovet, Luberon, France (Louchart *et al.* 2011)

**Phylogenetic placement justification:** Remarkable similarity in the skull morphology, including the rostrum indicate close affinities with *Pelecanus* (Louchart *et al.* 2011).

**Minimum age:** 28.25 Ma

**Minimum age justification:** based on Rupelian.

**First occurrences:**

- *Pelecanus* sp. partial skull very similar to that of a modern *Pelecanus* from Rupelian strata (33.00 - 28.25 Ma) at Pichovet, Luberon, France (Louchart *et al.* 2011).
- *Goliathia andrewsi* Lambrecht, 1930, ulna and distal tarsometatarsus from the Jebel Qatrani Formation, Quarry M (early Oligocene 30.2 - 29.5 Ma), Fayum, Egypt (Rasmussen *et al.* 1987).
- *Pelecanus tirarensis*, Miller 1966 from the Namba Formation at Lake Pinpa (late Oligocene, 26–24 Ma), Australia (Rich & van Tets 1981).
- *Pelecanus* sp. incomplete quadrate from the Pisco Formation (late Miocene, 6 Ma), Pisco, Peru (Altamirano-Sierra 2013). Larger than *occidentalis* but approaching smaller individuals of *thagus* in size, thus larger than the Luberon fossil; differs from *schreiberi* in details of the morphology of the quadratum (Altamirano-Sierra 2013).
- *Pelecanus schreiberi* Olson 1999, distal femur from the Yorktown Formation (early Pliocene, 5.3 - 3.6 Ma), North Carolina, USA (Olson 1999).
- *Pelecanus cautleyi* Davies 1880 and *Pelecanus sivalensis* Davies 1880, distal ulna, distal and proximal radius and femur from Siwalik Hills (early Pliocene 5.3 - 3.6 Ma), India (Lydekker 1891).
- *Scopus umbretta* remains from Ankilitelo Cave (0.013 - 0.002 Ma) Madagascar (Goodman *et al.* 2013).

**Fossil record remarks:**

**Clade age estimation:**

One-sample Kolmogorov-Smirnov test

data: Mages

D = 0.39991, p-value = 0.1598

alternative hypothesis: two-sided

Quantiles:

0% 50% 95% 97.5%

29.54 34.17 47.23 51.81

Parameters of the skewStudent function:

offset xi omega alpha nu

29.539 30.033 5.589 21.733 3.330

**
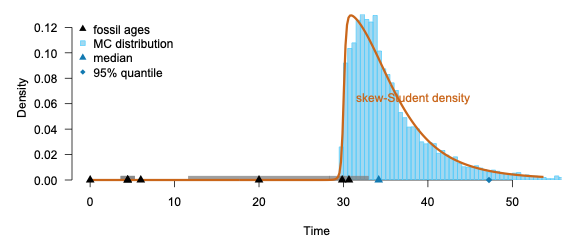
**

**References:**

Altamirano-Sierra, A. J. 2013. Primer registro de pelícano (Aves: Pelecanidae) para el Mioceno tardío de la formación Pisco, Perú. Bulletin de l'Institut Français d'Etudes Andines 42 (1):1-12.

Goodman, S.M., Raherilalao, M.J. and Muldoon, K., 2013. Bird fossils from Ankilitelo Cave: inference about Holocene environmental changes in Southwestern Madagascar. Zootaxa 3750(5):534-548.

Louchart, A., N. Tourment, J. Carrier 2011. The Earliest Known Pelican Reveals 30 Million Years of Evolutionary Stasis in Beak Morphology. Journal of Ornithology 150: 15–20.

Lydekker, R. 1891. Catalogue of the Fossil Birds in the British Museum (Natural History). London, UK.

Olson, S. L. 1999. A new species of pelican (Aves: Pelecanidae) from the lower Pliocene of North Carolina and Florida. Proceedings of the Biological Society of Washington 112:503-509.

Rasmussen, D. T., Olson, S. L. & E. L. Simons. 1987. Fossil birds from the Oligocene Jebel Qatrani Formation, Fayum Province, Egypt. Smithsonian Contributions to Paleobiology 62:1-20. Seiffert, E. R. 2006. Revised age estimates for the later Paleogene mammal faunas of Egypt and Oman. Proceedings of the National Academy of Sciences of the United States of America 103:5000-5005.

Rich, P. V. & J. van Tets. 1981. The fossil pelicans of Australia. Records of the South Australian Museum 18:235–264.

**Calibration: crown Ardeidae**

**Category: A**

**MRCA of:** *Cochlearius cochlearius* and *Egretta garzetta*

**Clade definition in tree:** *Cochlearius cochlearius*, *Egretta garzetta*

**Oldest fossil:** *Proardea deschutteri* Mayr *et al.* 2018, a left distal tarsometatarsus (Holotype: Institut Royal des Sciences Naturelles de Belgique Av 129) from the Boutersem Sand Member of the Borgloon Formation at Hoogbutsel near Leuven, Belgium.

**Phylogenetic placement justification:** Two cladistic analyses (54 characters for 26 species and 88 characters for 34 species) placed *P. deschutteri* within crown Ardeidae (Mayr *et al.* 2018). The second analysis, in particular, shows *Cochlearius* as the sister group including the rest of Ardeidae including *P. deschutteri*.

**Minimum age:** 32.02 Ma

**Minimum age justification:** the Borgloon Formation corresponds with nannoplankton zone NP22 (Vandenberghe *et al.* 2002) and thus is between 32.02 and 32.92 Ma in age (Agnini *et al.* 2017).

**First occurrences:**

- *Proardea deschutteri* Mayr *et al.* 2018, a left distal tarsometatarsus (Holotype: Institut Royal des Sciences Naturelles de Belgique Av 129) from the Boutersem Sand Member of the Borgloon Formation at Hoogbutsel near Leuven, Belgium.
- *Nycticorax* sp., right tarsometatarsus from the Qatrani Formation, Quarry M (30.2 - 29.5 Ma, Seiffert 2006), Fayum, Jebel, Egypt (Rasmussen *et al.* 1987).
- *Matuku otagoense* Scofield *et al.* 2010 and *Pikaihao bartlei* Worthy *et al.* 2013 multiple fragmented bones from the Bannockburn Formation at Site HH4, Home Hills Station (early Miocene, 18.26–15.97Ma), Otago, New Zealand (Scofield *et al.* 2010, Worthy *et al.* 2013).
- *Proardeola walkeri* from the early Miocene (16 - 11.6 Ma) of Li Mae Long, Lamphun province, Thailand (Cheneval *et al.* 1991).
- *Ardea polkensis*, from the Bone Valley Member of the Peace River Formation (Barstovian 16 - 13.6 Ma) Locality 2, near Brewster, Polk County, Florida, USA (Brodkorb 1955).
- *Ardea picata*, distal tibiotarsus from the Allingham Formation (early Pliocene, 5.3 - 3.6 Ma), northwest of Charters Towers, Queensland, Australia (Boles & Mackness 1994).
- *Syrigma sanctimartini* Campbell 1979 left coracoid from the late Pleistocene (0.126 - 0.0117 Ma) of Talara, Peru (Campbell 1979).
- *Ardea cinerea*, *Ardea purpurea*, *Ardea humboldti* from the Holocene of Ampoza (0.002 - 0.00 Ma), Madagascar (Goodman 1999)

**Fossil record remarks:**

**Clade age estimation:**

One-sample Kolmogorov-Smirnov test

data: Mages

D = 0.27606, p-value = 0.4924

alternative hypothesis: two-sided

Quantiles:

0% 50% 95% 97.5%

32.06 35.40 46.89 50.72

Parameters of the skewStudent function:

offset xi omega alpha nu

32.056 32.296 4.227 29.836 2.688


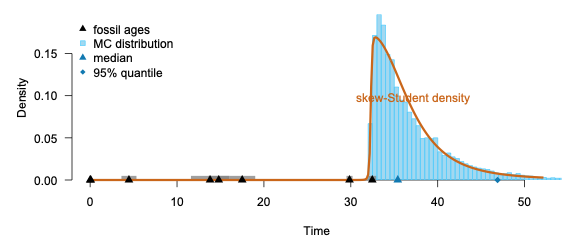


**References:**

Agnini, C., S. Monechi & I. Raffi. 2017. Calcareous nannofossil biostratigraphy: historical background and application in Cenozoic chronostratigraphy. Lethaia 50(3):447-463.

Boles, W. E. & B. Mackness 1994 Birds from the bluff downs local fauna, Allingham formation, Queensland, Rec. South Austral. Mus. 27(2):139-149.

Brodkorb, P. 1955. The avifauna of the Bone Valley Formation. Florida Geological Survey Report of Investigations 14:1-57.

Campbell, K. E. 1979. The non-passerine Pleistocene avifauna of the Talara Tar Seeps, northwestern Peru. Royal Ontario Museum Life Sciences Contribution 118:1-203.

Cheneval, J., L. Ginsburg, C. Mourer-Chauvire, B. Ratanasthien 1991. The Miocene avifauna of the Li Mae Long locality, Thailand: systematics and paleoecology.Journal of Southeast Asian Earth Sciences 6:117–126.

Goodman, S.M. 1999. Holocene bird subfossils from the sites of Ampasambazimba, Antsirabe and Ampoza, Madagascar:Changes in the avifauna of south central Madagascar over the past few millennia. In: Adams, N.J. & Slotow, R.H. (eds) Proc. 22 Int. Ornithol. Congr., Durban: 3071-3083. Johannesburg: BirdLife South Africa.

Harrison C. J. O. 1979 The herons (Ardeidae) of the Old World Tertiary. In C. J. O. Harrison & C. A.Walker (eds.) Studies in Tertiary avian paleontology. Tertiary Research, Special Paper 5: 11-17.

Mayr, G., V. L. de Pietri, R. P. Scofield & T. Smith 1919. A fossil heron from the early Oligocene of Belgium: the earliest temporally well-constrained record of the Ardeidae. Ibis 161:79–90.

Rasmussen, D. T., Olson, S. L. & E. L. Simons. 1987. Fossil birds from the Oligocene Jebel Qatrani Formation, Fayum Province, Egypt. Smithsonian Contributions to Paleobiology 62:1-20.

Scofield, R.P., T. H. Worthy & A. J. D.Tennyson 2010 A heron (Aves: Ardeidae) from the early Miocene St Bathans Fauna of southern New Zealand, Rec. Austral. Mus. 62:89-104.

Seiffert, E. R. 2006. Revised age estimates for the later Paleogene mammal faunas of Egypt and Oman. Proceedings of the National Academy of Sciences of the United States of America 103:5000-5005.

Vandenberghe, N., J. Herman & E. Steurbaut 2002. Detailed analysis of the Rupelian Ru-1 transgressive surface in the Type area (Belgium). Pp. 67-83 in Gürs, K. (ed) Northern European Cenozoic Stratigraphy. Proceeding of the 8^th^ Biannual Meeting of the RCBBS/RCNPS, Flintbek.

Worthy, T. H., J. P. Worthy, A. J. Tennyson & R. P. Scofield 2013. A bittern (Aves: Ardeidae) from the early Miocene of New Zealand. Paleontological Journal 47(11):1331-1343.

##

## TELLURAVES

**Calibration: crown Accipitriformes**

**Category: B**

**MRCA of:** Sagittariidae and Accipitridae

**Clade definition in tree:** *Sagittarius serpentarius*, *Aquila chrysaetos*

**Oldest fossil:** ?Accipitridae, gen. et sp. indet., proximal end of right tarsometatarsus (IRSNB Av 182), ungual pedal phalanges (IRSNB 184, IRSNB 183, Mary 2019)

**Minimum age**: 50.5 Ma

**Minimum age justification:** “from marine sediments of Tielt Formation in northwestern Belgium, Ampe Quarry (51^o^ 00’ 45” N, 3 ^o^ 13’ 56” E)…[found] in 20 to 80 centimeter thick layers of shelly glauconite sands at the base of the Egemkapel Clay Member (Steurbaut 1998; Smith & Smith 2013). Based on microfossil analyses, the Egemkapel Clay Member was referred to the Upper NP nannoplankton zone (subzone VI; mid Ypresian, about 50.5-52 Ma; Steurbaut 1998; King et al 2016)…a few remains of mammals, which allowed biochronological referral of the Egemkapel Clay Member to the reference level MP8+9 of the Mammalian scale for the European Paleogene (Smith & Smith 2013; King et al 2016).” Mayr, G., & Smith, T. 2019

**Phylogenetic placement justification:** IRSNB Av 182**: “**The hypotarsus corresponds to that of extant Accipitridae in that it formed two widely separated crests, with the lateral one being mediolaterally broader than the medial crest (although the crests themselves are broken, the remaining portions allow inferences on their position and size). The small foramen vascularis proximalia are situated close together in the deep sulcus extensorius, with the dorsal opening of the lateral foramen being positioned slightly farter proximally than that of the medial foramen. The impressus retinaluci extensorii for two low and short ridges. The tuberositas musculi tibialis cranialis is centrally positioned as in extant Accipitridae, but it is much less prominent in the fossil. The crista medianoplantaris is better developed than in extant Accipitridae. The preserved section of the shaft gradually narrows towards the distal end of the bone. The dorsoplantarly flat shaft forms a very narrow medial margin and a much deeper lateral one.” (Mayr 2019)

IRSNB 184, IRSNB 183: “The phalanges correspond to the tarsometatarsus fragment in size and resemble the ungual phalanges of extant Accipitridae. … All three [*sic*] phalanges differ from the ungual phalanges of strigiform birds in that the plantar surface of the corpus is flat (rounded in Strigiformes). The sulcus neurovascularis is laterally closed and exits with a foramen next to the tuberculum flexorium.” (Mary 2019)

In sum, the specimens share important similarities with accipitrids, however, similarities and differences with other members of the Accipitriformes (Sagittarius, Pandion) were not analyzed. While Sagittarius lacks hypotarsal crests, Pandion has full ossified tendinal canalas. Because the hypotarsal crests in the fossil are actually broken, a condition similar to that of Pandion cannot be ruled out. Finally, it is also possible that an incompletely ossified hypotarsal canal is ancestral to the Accipitridae-Pandionidae clades. Therefore, adopted a conservative approach and assign the fossils to crown Accipitriformes.

**First occurrences:**

- IRSNB Av 182-184, proximal end of tarsometatarsus and ungual pedal phalanges from the Tilet Formation at Ampe Quarry (52 - 50.5 Ma), Belgium (Mary 2019).
- *Buteo grangeri* Wetmore & Case 1934, from Pass Creek Oreodon Zone (Early Oligocene 33.9 - 33.3 Ma), South Dakota, USA..
- Duke University Primate Center 3082, distal humerus from the Jebel Qatrani Formation, Quarry M, (Early Oligocene, 30.2 - 29.5 Ma, Seiffert 2006), Fayum, Egypt (Olson & Simons 1987).
- *Venerator dementjevi* Kurochkin 1968, Hsanda Gol Formation (Oligocene 33.9 - 23.03 Ma), Mongolia.
- *Archaehierax sylvestris* Matcher et al. 2021, 63 associated elements and fragments from a single skeleton from the Namba Formation (Late Oligocene 26 - 24 Ma), Lake Pinpa, Australia.
- MNZ S42811 distal tibiotarsus from the Bannockburn Formation, St. Bathans Fauna (Early Miocene, Altonian, 18.26-15.97 Ma) Otago, New Zealand (Worthy et al. 2007).
- *Geranoaetus* sp. from La Pastosa, Puerto Madryn Formation (16 - 5.3 Ma, Dozo et al. 2010), Chubut, Argentina (Agnolín 2006).
- *Accipiter madagascariensis*, Ankilitelo Cave (latest Pleistocene-Holocene, 0.013 - 0.003 Ma), Madagascar (Goodman *et al.* 1993, 2013).

**Clade age estimation:**

One-sample Kolmogorov-Smirnov test

data: Mages

D = 0.18806, p-value = 0.8934

alternative hypothesis: two-sided

Quantiles:

0% 50% 95% 97.5%

58.94 65.78 87.89 95.10

Parameters of the skewStudent function:

offset xi omega alpha nu

58.941 59.868 7.981 17.620 2.705


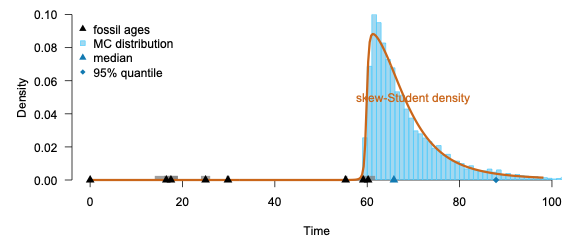


**References:**

Agnolín, F.L. 2006. Notas sobre el registro de Accipitridae (Aves, Accipitriformes) fósiles argentinos. Stud. Geol. Salmant 42:67–80.

Dozo, M. T., P. Bouza, A. Monti, L. Palazzesi, V. Barreda, G. Massaferro, R.A. Scasso and C.P. Tambussi. 2010. Late Miocene continental biota in Northeastern Patagonia (Peninsula Valdés Chubut Argentina). Palaeogeography, Palaeoclimatology, Palaeoecology 297:100-109

Goodman, S. M., & Ravoavy, F. 1993. Identification of bird subfossils from cave surface deposits at Anjohibe, Madagascar, with a description of a new giant Coua (Cuculidae: Couinae). Proceedings of the Biological Society of Washington, 106(1):24-33.

Goodman, S.M., Raherilalao, M.J. and Muldoon, K., 2013. Bird fossils from Ankilitelo Cave: inference about Holocene environmental changes in Southwestern Madagascar. Zootaxa 3750(5):534-548.

King, C., Barry, T.L., & Gale, A.S. (2016) A revised correlation of Tertiary rocks in the British Isles and adjacent areas of NW Europe. Geological Society of Europe Special Reports, 27:1-719.

Kurochkin, E. N. 1968. [Fossil remains of birds from Mongolia]. Ornitologija 9:323-330.

Mather, E. K. Michael S. Y. Lee, Aaron B. Camens & Trevor H. Worthy (2021): An exceptional partial skeleton of a new basal raptor (Aves: Accipitridae) from the late Oligocene Namba formation, South Australia. Historical Biology DOI: 10.1080/08912963.2021.1966777

Mayr, G., & Smith, T. 2019 Adiverse bird assemblage from the Ypresian of Belgium furthers knowledge of Early Eocene avifaunas of the North Sea Basin. N. Jb. Geol. Paläont. Abh. 291/3 (2019), 253-281.

Rasmussen, D. T., Olson, S. L. & E. L. Simons. 1987. Fossil birds from the Oligocene Jebel Qatrani Formation, Fayum Province, Egypt. Smithsonian Contributions to Paleobiology 62:1-20.

Seiffert, E. R. 2006. Revised age estimates for the later Paleogene mammal faunas of Egypt and Oman. Proceedings of the National Academy of Sciences of the United States of America 103:5000-5005.

Smith, T., & Smith, R. 2013 A land micro-fauna from the Early Eocene marine Egem deposits (NP12, Belgium) and the first occurrence of the peradectid marsupial *Armintodelphys* outside of North America. Geol. Belg., 16: 302-310.

Steurbaut,E. 1998. High-resolution holostratigraphy of Middle Paleocene to Early Eocene strata in Belgium and adjacent areas. Palaeontolographica Abt. A Paläozool. Stratigr., 247: 91-156.

Wetmore A. & E. C. Case. 1934. A new fossil hawk from the Oligocene beds of South Dakota. Contributions from the Museum of Paleontology, University of Michigan 4:129-132.

Worthy, T. H., A. J. D. Tennyson, C. Jones, J. A. McNamara, and B. J. Douglas. 2007. Miocene waterfowl and other birds from Central Otago, New Zealand. Journal of Systematic Palaeontology 5:1–39.

**Calibration: stem Strigiformes**

**Category: B** (only used in trees in which Strigiformes and Accipitriformes are sister taxa)

**MRCA of:** Strigiformes + Accipitriformes

**Clade definition in tree:** *Tyto alba, Aquila chrysaetos*

**Oldest fossil: *Ogygoptynx wetmorei***, tarsometatarsus (AMNH 2653) from the Late Paleocene of Mason Pocket (Ti-4a, Tiffanian NALMA), Colorado, USA (Rich & Bohaska 1976).

**Phylogenetic placement justification:** Broadly and deeply excavated anterior metatarsal groove; the single, slender calcaneal ridge; the shallow, narrow middle trochlea relative to the inner and outer trochleae; and the inner trochlea extending distally nearly as far as, or farther than, the middle trochlea.

**Minimum age:** 58.8 Ma.

**Age justification:** *Plesiadapis churchilli* biozone, Chron C26r: 58.8-59.5 Ma (Secord et al. 2006).

**First occurrences:**

- *Ogygoptynx wetmorei*, tarsometatarsus (AMNH 2653) from the Late Paleocene of Mason Pocket (Ti-4a, Tiffanian NALMA, 58.8-59.5 Ma Secord et al. 2006), Colorado, USA (Rich & Bohaska 1976).
- *Berruornis halbedeli* Mayr 2007 Right tarsometatarsus, lacking articular ends from (61.7 - 58.7 ), Germany: Walbeck (Mayr (2007, 2009)
- *Eostrix tsaganica* Kurochkin & Dyke 2011 (Protostrigidae) distal tarsometatarsus from the Bumban member of the Naranbulag Formation at Tsagaan Khushuu (55.7 - 54.97 Ma) Mongolia.
- cf. Selenornithidae from the Jebel Qatrani Formation (30.2 - 29.5 Ma), Fayum, Egypt (Smith et al. 2020).
- *Archaehierax sylvestris* (Accipitridae) multiple bones from the Namba Formation (26 - 24 ), Lake Pinpa, Australia (Mather et al. 2021).
- Accipitridae indet., tibiotarsus and ulna from Bannockburn Formation, St Bathans Fauna (1826-15.97 Ma), Otago, New Zealand (Worthy et al. 2007).
- Strigidae indet. from the Pinturas Formation (19 - 14 Ma), Santa Cruz, Argentina (Chiappe 1991).
- *Asio madagascariensis*, *Otus rutilus*, *Ninox superciliaris* remains from Ankilitelo Cave (0.013 - 0.005 Ma), Madagascar (Goodman et al. 2013).

**Clade age estimation:**

One-sample Kolmogorov-Smirnov test

data: Mages

D = 0.28731, p-value = 0.4429

alternative hypothesis: two-sided

Quantiles:

0% 50% 95% 97.5%

50.54 55.83 74.43 80.54

Parameters of the skewStudent function:

offset xi omega alpha nu

50.540 51.049 6.467 24.212 2.615

**
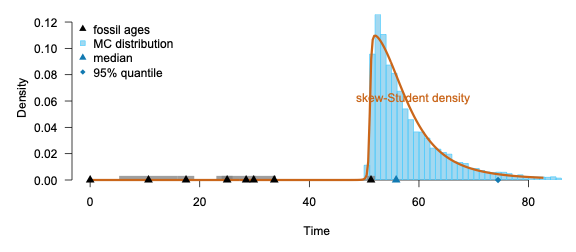
**

**References:**

Chiappe, L. M. 1991. Fossil birds from the Miocene Pinturas Formation of Southern Argentina. Jour- nal of Vertebrate Paleontology 11, 21A–22A.

Goodman, S. M., Raherilalao, M. J. and Muldoon, K., 2013. Bird fossils from Ankilitelo Cave: inference about Holocene environmental changes in Southwestern Madagasca. Zootaxa 3750(5):534-548.

Kurochkin EN, Dyke GJ 2011 The first fossil owls (Aves: Strigiformes) from the Paleogene of Asia and a review of the fossil record of Strigiformes. Paleontol J 45:445–458.

Mather, E. K., Michael S. Y. Lee, Aaron B. Camens & Trevor H. Worthy (2021): An exceptional partial skeleton of a new basal raptor (Aves: Accipitridae) from the late Oligocene Namba formation, South Australia, Historical Biology, DOI: 10.1080/08912963.2021.1966777

Mayr G. 2002. An owl from the Paleocene of Walbeck, Germany. Mitteilungen aus dem Museum für Naturkund in Berlin. *Geowissenschaftliche Reihe Foss. Rec.* **5**, 283–288

Mourer-Chauviré, C. 1994. A large owl from the Palaeocene of France. *Palaeontology* **37**, 339–348..

Rich,P. V., D. J. Bohaska, The world’s oldest owl: A new strigiform from the Paleocene of southwestern Colorado. *Smithson. Contrib. Paleobiol.* **27**, 87–93 (1976).

Secord, R., P. D. Gingerich, M. E. Smith, W. C. Clyde, P. Wilf, and B. S. Singer (2006) Geochronology and mammalian stratigraphy of middle and upper Paleocene continental strata, Bighorn Basin, Wyoming. American Journal of Science, 306: 211–245.

Seiffert, E. R. **2006** Revised Age Estimates for the Later Paleogene Mammal Faunas of Egypt and Oman. Proc. Natl. Acad. Sci. USA 103, 5000–5005.

Smith,N. A., T. A. Stidham, J. S. Mitchell (2020) The First Fossil Owl (Aves, Strigiformes) From the Paleogene of Africa. Diversity 2020, 12, 163; doi:10.3390/d12040163.

Worthy, T. H., A. J. D. Tennyson, C. Jones, J. A. McNamara, and B. J. Douglas. 2007. Miocene waterfowl and other birds from Central Otago, New Zealand. Journal of Systematic Palaeontology 5:1–39.

**Calibration: crown Coraciimorphae**

**Category: A**

**MRCA of:** Coliiformes and Coraciiformes

**Clade definition in tree:** *Colius_striatus, Eurystomus_gularis*

**Oldest fossil:** *Tsidiiyazhi abini* Ksepka *et al.* 2017, an associated partial skeleton from the Ojo Encino Member of the Nacimiento Formation at the West Flank of Torreon Wash, New Mexico, USA.

**Phylogenetic placement justification:** A cladistic analysis of 111 osteological characters and 48 species representing all major groups of Telluraves, including 16 fossil taxa, placed *Tsidiiyazhi* in a clade of stem Coliiformes with low bootstrap support (59%) but robust to variations in the basal topology of Telluraves (Ksepka *et al.* 2017)

**Minimum age**: 62.2 Ma

**Minimum age justification:** L-6898 was located within magnetochron C27N, constraining the absolute age to between 62.221 and 62.517 Ma (Ksepka *et al.* 2017).

**First occurrences:**

- *Tsidiiyazhi abini* Ksepka *et al.* 2017 associated partial skeleton from the Ojo Encino Member of the Nacimiento Formation (62.52 - 62.22 Ma), New Mexico, USA.
- *Septentrogon madseni* Kristoffersen 2002, fragmentary cranium without rostrum or palatines from levels containing ash layers +25 through +30 in the Silstrup Mb. (55.4 Ma, see justification under *Septencoracias*), Fur Formation, Denmark.
- *Ueekenkcoracias tambussiae* Degrange, Pol, Puerta & Wilf 2021, incomplete right hind limb, preserved in two slabs as part and counterpart (MPEF-PV 10991) from the Huitrera Formation at Laguna del Hunco, quarry LH27 (52.2 Ma) Chubut prov., Argentina.
- *Foshanornis songi* Zhao *et al.* 2015, semiarticulated nearly complete skeleton from the Buxin Formation (55.8 - 48.6 Ma), China.
- Coliidae indet, fragmented distal tarsometatarsus from Grillental (20 Ma), Northern Sperrgebiet, Namibia (Mourer-Chauviré 2008).
- Halcyonidae, a complete carpometacarpus from Last Minute Site, Faunal Zone C (16 - 11.6 Ma), Riversleigh, Australia (Boles 1997). The bone differs from that of all modern genera examined including *Halcyon* (Boles 1997).
- *Leptosomus discolor* remains from Ankilitelo Cave (0.013 - 0.005 Ma), Madagascar (Goodman *et al.* 2013).
- *Halcyon sancta* two right humeri from the Holocene of Gilles Cave (0.0036 - 0.0032 Ma), New Caledonia (Balouet & Olson 1989)

**Fossil record remarks:**

**Clade age estimation:**

One-sample Kolmogorov-Smirnov test

data: Mages

D = 0.24984, p-value = 0.6142

alternative hypothesis: two-sided

Quantiles:

0% 50% 95% 97.5%

62.24 67.99 91.89 100.30

Parameters of the skewStudent function:

offset xi omega alpha nu

62.240 62.334 7.442 137.954 2.258

**
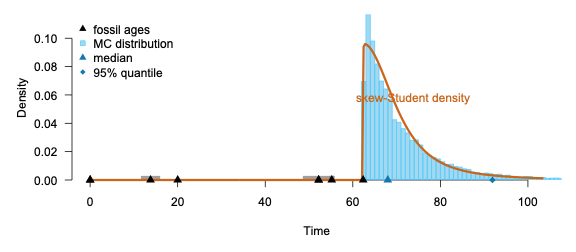
**

**References:**

Balouet, J. C., & S. L. Olson 1989 Fossil birds from late Quaternary deposits in New Caledonia. Smithsonian Contributions to Zoology 469:1-38.

Boles, W. E., 1997. A kingfisher (Halcyonidae) from the Miocene of Riversleigh, northwestern Queensland, with comments on the evolution of kingfishers in Australo-Papua. Memoirs of the Queensland Museum 41:229-234.

Degrange, F. J., D. Pol, P. Puerta & P. Wilf 2021. Unexpected larger distribution of paleogene stem‐rollers (Aves, Coracii): new evidence from the Eocene of Patagonia, Argentina. Scientific Reports 11:1363.

Goodman, S. M., M. J. Raherilalao & K. Muldoon 2013. Bird fossils from Ankilitelo Cave: inference about Holocene environmental changes in Southwestern Madagascar. Zootaxa 3750(5):534-548.

Kristoffersen, A. V. 2002. An early Paleocene Trogon (Aves: Trogoniformes) from the Fur Formation, Denmark. Journal of Vertebrate Paleontology 22: 661–666.

Ksepka, D. T., T. A. Stidham & T. E. Williamson. 2017 Early Paleocene landbird supports rapid phylogenetic and morphological diversification of crown birds after the K–Pg mass extinction. Proceedings of the National Academy of Sciences 114(30):8047-8052.

Mourer-Chauviré, C. 2008. Birds (Aves) from the Early Miocene of the Northern Sperrgebiet, Namibia. Geology and Palaeobiology of Namib Desert, Southwestern Africa 3:147-167.

Zhao, T., G. Mayr, M. Wang & W. Wang. 2015. A trogon-like arboreal bird from the early Eocene of China. Alcheringa 39:287-294.

**Calibration: Bucerotes**

**Category: A**

**MRCA of:** Bucertotiformes and Upupiformes

**Clade definition in tree:** *Upupa epops, Buceros rhinoceros*

**Oldest fossil:** *Messelirrisor grandis* pectoral girdle and limb (Type specimen: SMNH PAL 3803) and referred complete skeleton and pigmented feathers from the Lower Middle Eocene (MP11; 47 MY), Grube Messel, Hessen, Germany (Mayr 1998, 2000, 2006).

**Phylogenetic placement justification:** A cladistic analysis of 122 anatomical and molecular characters for 34 avian taxa placed *Messelirrisor grandis* as sister to the Upupiformes (Mayr 2006).

**Minimum age**: 47.41 Ma

**Minimum age justification:** Correlations between astronomical orbital cycles and the cyclical changes in pollen deposits together with a revised calibration of ^40^Ar/^39^Ar dates for the initial volcanic explosion that formed Messel’s maar lake indicate that fossil deposition in the oil shales started at 48.05 Ma or 48.25 Ma, depending on the calibration, and ended at 47.41 or 47. 61 Ma (Lenz *et al*. 2015).

**First occurrences:**

- *Messelirrisor grandis* pectoral girdle and limb (Type specimen: SMNH PAL 3803) and referred complete skeleton and pigmented feathers from the Grube Messel (48.25 - 47.41 Ma), Hessen, Germany (Mayr 1998, 2000, 2006).
- cf. *Tockus*, right coracoid lacking extremitas sternalis (KNM-MB 21997) from Maboko Main, bed 3, green sand (pit 32), Middle Miocene (15.97 - 11.60 Ma) of Kenya (Mayr 2014).
- *Upupa marginata* from Ankilitelo Cave (13 - 5 ka), Madagascar (Goodman et al. 2013).
- *Aceros plicatus* from Balof ( 0.014 - 0 Ma), New Ireland, Papua New Guinea (Steadman et al. 1999).
- Several extant species of Bucerotidae from the Great Cave of Niah (0.036 - 0 Ma), Sarawak (Stimpson 2012)

**Fossil record remarks:** Record not uniform, One-sample Kolmogorov-Smirnov test: D = 0.6, p-value = 0.03. Quaternary record excluded

Quantiles:

0% 50% 95% 97.5%

47.46 62.00 163.70 230.70

Parameters of the skewStudent function:

offset xi omega alpha nu

47.457 47.719 15.571 83.627 1.266

**
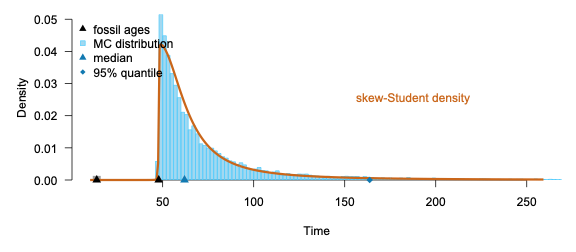
**

**References:**

Goodman, S.M., Raherilalao, M.J. and Muldoon, K., 2013. Bird fossils from Ankilitelo Cave: inference about Holocene environmental changes in Southwestern Madagasca. Zootaxa 3750(5):534-548.

Ksepka, D.T. and Clarke, J.A. 2015. Phylogenetically vetted and stratigraphically constrained fossil calibrations within Aves. Palaeontologia Electronica, 18.1.3FC.

Lenz, O. K., Wilde, V., Mertz, D. F., & Riegel, W. 2015. New palynology-based astronomical and revised ^40^Ar/^39^Ar ages for the Eocene maar lake of Messel (Germany). International Journal of Earth Sciences 104(3):873-889.

Mayr, G. 1998. Coraciiforme" und "piciforme" Kleinvogel aus dem Mittel-Eozan der Grube Messel (Hessen, Deutschland). Courier Forschungsinstitut Senckenberg 205:1-101.

Mayr. 2000. Tiny hoopoe-like birds from the Middle Eocene of Messel (Germany). *The Auk* 117(4):964-970

Mayr, G. 2006. New specimens of the Eocene Messelirrisoridae (Aves: Bucerotes), with comments on the preservation of uropygial gland waxes in fossil birds from Messel and the phylogenetic affinities of Bucerotes. Paläontologische Zeitschrift, 80:390-405.

Steadman, D. W., White, J. P., & Allen, J. 1999. Prehistoric birds from New Ireland, Papua New Guinea: extinctions on a large Melanesian island. Proceedings of the National Academy of Sciences 96(5):2563-2568.

Stimpson, C. M. 2012. Local scale, proxy evidence for the presence of closed canopy forest in North-western Borneo in the late Pleistocene: Bones of Strategy I bats from the archaeological record of the Great Cave of Niah, Sarawak. Palaeogeography, Palaeoclimatology, Palaeoecology 331–332:136–149

**Calibration: crown Coracii**

**Category: A**

**MRCA of:** Meropidae and Coracioidea

**Clade definition in tree:** *Merops nubicus*, *Eurystomus gularis*

**Oldest fossil:** *Septencoracias morsensis* Bourdon et al. 2016 nearly complete 3-D-preserved skeleton lacking sternum and most shoulder girdle (MGUH.VP 9509) from the Fur Formation at Moclay pit (Klovbakker), Island of Mors, Jutland, Denmark.

**Phylogenetic placement justification:** Assigned to the extinct roller family Primobucconidae based on three derived character and a cladistics analysis placing *Septencoracias* as a stem Coraci sister to *Primobucco* (Bourdon *et al.* 2016). The Coraciiformes are paraphyletic in relation to Bucerotiformes and Piciformes in the osteological tree because of the basal position of Coraciidae and Brachypteraciidae (Bourdon *et al.* 2016). A referred specimen revealed some similarities with Meropidae (Mayr 2021), which may represent ancestral conditions.

**Minimum age**: 55.4 Ma

**Minimum age justification:** MGUH.VP 9509 derives from immediately above ash layer +30 in Fur Formation’s Silstrup Mb. Ash layer +30 is only slightly younger than ash layer +19, which has been estimated to be 0.2 Ma younger than the radiometrically dated ash layer -17 (Westerhold et al. 2009), of absolute date 55.6 ± 0.12 Ma (corrected ages based on ^40^Ar/^39^Ar dating, Stokke *et al.* 2020). Therefore, we adopted the estimated age of layer +19, 55.4 Ma as the fossil age.

**First occurrences:**

- *Septencoracias morsensis* Bourdon et al. 2016 nearly complete 3-D-preserved skeleton lacking sternum and most shoulder girdle (MGUH.VP 9509) from the Fur Formation at Moclay pit (Klovbakker, 55.4 Ma), Island of Mors, Jutland, Denmark.
- *Ueekenkcoracias tambussiae* Degrange, Pol, Puerta & Wilf 2021, incomplete right hind limb, preserved in two slabs as part and counterpart (MPEF-PV 10991) from the Huitrera Formation at Laguna del Hunco, quarry LH27 (52.2 Ma) Chubut prov., Argentina (Degrange *et al.*  2021).
- *Primobucco mcgrewi* Brodkorb 1970, *Paracoracias occidentalis* Clarke *et al.* 2009 (Coraci) from the Fossil Butte Member (51.97 ± 0.16 Ma) of the Green River Formation, USA (Clarke *et al.* 2009, Ksepka & Clarke 2010).
- Halcyonidae, a complete carpometacarpus from Last Minute Site, Faunal Zone C (16 - 11.6 Ma), Riversleigh, Australia (Boles 1997). The bone differs from that of all modern genera examined including *Halcyon* (Boles 1997).
- Alcedinidae gen. et sp. right tarsometatarsus lacking proximal end (KNM-MB 22115) from Maboko Main, bed 5, white clay, pit 51 (Middle Miocene 15.97 - 11.60 Ma), Kenya (Mayr 2014).
- *Halcyon smyrnensis* (Halcyonidae) from Kebara Cave (0.126 Ma - 0.012 Ma), Israel (Tchernov 1962)
- *Eurystomus glaucurus* (Coraciidae) from Ankilitelo Cave (0.013 Ma - 0.005 Ma), Madagascar (Goodman *et al.* 2013).
- *Halcyon sancta*, humerus from upper layer of Gille Cave (Holocene 0.0036 - 0.0032 Ma), New Caledonia (Balouet & Olson 1989).

**Fossil record remarks:**

**Clade age estimation:**

One-sample Kolmogorov-Smirnov test

data: Mages

D = 0.38373, p-value = 0.1439

alternative hypothesis: two-sided

Quantiles:

0% 50% 95% 97.5%

54.46 59.91 79.49 86.59

Parameters of the skewStudent function:

offset xi omega alpha nu

54.458 54.794 6.691 37.261 2.465

**
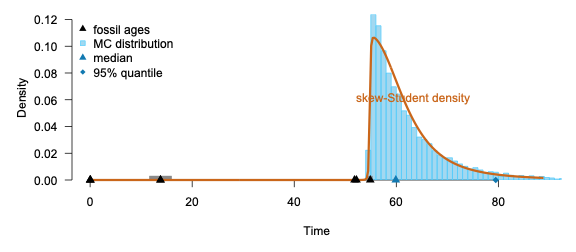
**

**References:**

Boles, W. E. (1997). A kingfisher (Halcyonidae) from the Miocene of Riversleigh, northwestern Queensland, with comments on the evolution of kingfishers in Australo-Papua. Memoirs of the Queensland Museum 41:229–234.

Bourdon, E., A. V. Kristoffersen & N. Bonde. 2016. A roller-like bird (Coracii) from the Early Eocene of Denmark. Scientific Reports 6(34050):1-9.

Clarke, J. A., D. T. Ksepka, N. A. Smith & M. A. Norell. 2009 Combined phylogenetic analysis of a new North American fossil species confirms widespread Eocene distribution for stem rollers (Aves, Coracii). Zool. J. Linn. Soc. 157:586-611.

Degrange, F. J., D. Pol, P. Puerta & P. Wilf 2021. Unexpected larger distribution of paleogene stem‐rollers (Aves, Coracii): new evidence from the Eocene of Patagonia, Argentina. Scientific Reports 11:1363.

Dyke & Lindow 2009. Taphonomy and abundance of birds from the lower Eocene Fur Formation Denmark. Geological Journal 44:365-373.

Goodman, S.M., Raherilalao, M.J. and Muldoon, K., 2013. Bird fossils from Ankilitelo Cave: inference about Holocene environmental changes in Southwestern Madagascar. Zootaxa 3750(5):534-548.

King, C. 2016. A revised correlation of Tertiary rocks in the British Isles and adjacent areas of NW Europe. Geol. Soc. Lond. Spec. Rep. 27: 1–719.

Ksepka, D. T., & J. A. Clarke 2010. *Primobucco mcgrewi* (Aves: Coracii) from the Eocene Green River Formation: New Anatomical Data from the Earliest Constrained Record of Stem Rollers. Journal of Vertebrate Paleontology 30(1):215-225.

Mayr, G., 2014. On the middle Miocene avifauna of Maboko Island, Kenya. Geobios 47(3):133-146.

Mayr, G., 2021. A partial skeleton of *Septencoracias* from the early Eocene London Clay reveals derived features of bee-eaters (Meropidae) in a putative stem group roller (Aves, Coracii). Palaeobiodiversity and Palaeoenvironments: 1-15.

Stokke, E. W., E. Liu & M. T. Jones 2020a. Evidence of explosive hydromagmatic eruptions during the emplacement of the North Atlantic Igneous Province. Volcanica 3(2):227-250.

Tchernov E. 1962. Palaeolithic avifauna in Palestine. Bulletin of the Research Council of Israel (B) 11: 95-131.

Westerhold, T., U. Röhl, H. K. McCarren, and J. C. Zachos 2009 Latest on the absolute age of the Paleocene–Eocene Thermal Maximum (PETM): New insights from exact stratigraphic position of key ash layers 19 and -17. Earth and Planetary Science Letters 287:412–419

**Calibration: Todidae - Alcedinidae**

**Category: A**

MRCA of: *Todus mexicanus, Chloroceryle aenea*

**Oldest fossil:** *Paleotodus escampsensis* [Mourer-Chauviré 1985](https://paleobiodb.org/classic/checkTaxonInfo?taxon_no=430350&is_real_user=1) (Late Eocene, Phosporites du Quercy, France)

**Phylogenetic placement justification:** Cladistic analysis of *Palaeotodus* *itardensis* confirmed a position of the genus as a stem Todidae (Ksepka et al. 2017, 2019)

**Minimum age:** 33.9

**Age justification:** MP19 (35.1 - 33.9, Mourer-CHauvire 2006).

**First occurrences:**

- *Paleotodus escampsensis* [Mourer-Chauviré 1985](https://paleobiodb.org/classic/checkTaxonInfo?taxon_no=430350&is_real_user=1) Phosporites du Quercy MP19 (Late Eocene 35.1 - 33.9 Ma), France.
- *Palaeotodus emryi* Olson 1976, Brule Formation (Early Oligocene 33.9 - 33.3 Ma), Wyoming (Olson 1976).
- Alcedinidae, right tarsometatarsus lacking proximal end (KNM-MB 22115) from Maboko Main, bed 5, white clay, pit 51 (Middle Miocene 15.97 - 11.60 Ma), Kenya (Mayr 2014).
- Halcyonidae, a complete carpometacarpus from Last Minute Site, Faunal Zone C (16 - 11.6 Ma), Riversleigh, Australia (Boles 1997). The bone differs from that of all modern genera examined including *Halcyon* (Boles 1997).
- *Halcyon smyrnensis* (Halcyonidae) from Kebara Cave (0.126 Ma - 0.012 Ma), Israel (Tchernov 1962)
- *Halcyon sancta*, humerus from upper layer of Gille Cave (Holocene 0.0036 - 0.0032 Ma), New Caledonia (Balouet & Olson 1989).

**Fossil record remarks:**

One-sample Kolmogorov-Smirnov test

data: Mages

D = 0.33029, p-value = 0.4343

alternative hypothesis: two-sided

Quantiles:

0% 50% 95% 97.5%

33.98 39.98 58.53 66.60

Parameters of the skewStudent function:

offset xi omega alpha nu

33.978 35.045 6.616 10.741 2.610


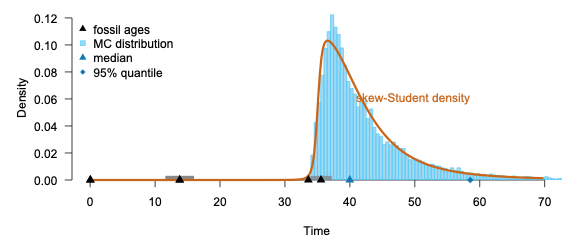


**References:**

Boles, W. E. (1997). A kingfisher (Halcyonidae) from the Miocene of Riversleigh, northwestern Queensland, with comments on the evolution of kingfishers in Australo-Papua. Memoirs of the Queensland Museum 41, 229–234.

Balouet, J. C., & S. L. Olson 1989 Fossil birds from late Quaternary deposits in New Caledonia. Smithsonian Contributions to Zoology 469:1-38.

Ksepka, D. T., T. A. Stidham & T. E. Williamson. 2017 Early Paleocene landbird supports rapid phylogenetic and morphological diversification of crown birds after the K–Pg mass extinction. Proceedings of the National Academy of Sciences 114(30):8047-8052.

Ksepka, D.T., L. Grande & G. Mayr 2019. Oldest finch-beaked birds reveal parallel ecological radiations in the earliest evolution of passerines. Current Biology 29(4):657-663.

Mayr, G. 2009b. Paleogene fossil birds. Berlin: Springer.

Mayr, G., 2014. On the middle Miocene avifauna of Maboko Island, Kenya. Geobios 47(3):133-146.

G. Mayr and N. Micklich. 2010. New specimens of the avian taxa Eurotrochilus (Trochilidae) and Palaeotodus (Todidae) from the early Oligocene of Germany. *Palaeontologische Zeitschrift* **84(10.1007/s1)**:387-395

Mourer-Chauviré, C. (1985) Les Todidae (Aves, Coraciiformes) des Phosphorites du Ouercy (France). *Palaeontology,* *Proceedings B* 88 (4), 407-414.

Olson, S. L. 1976. Oligocene fossils bearing on the origins of the Todidae and the Momotidae (Aves: Coraciiformes). In: Olson, S. L. (Ed.), Collected papers in avian paleontology honoring the 90th birthday of Alexander Wetmore. *Smithsonian Contributions to Paleobiology* 27: 111-119.

Tchernov E. 1962. Palaeolithic avifauna in Palestine. *Bulletin of the Research Council of Israel* (B) 11: 95-131.

**Calibration: crown Psittacopasseres**

**Category: A**

**MRCA of:** Psittaciformes and Passeriformes

**Clade definition in tree:** *Nestor notabilis*, *Passer domesticus*

**Oldest fossil:** *Eozygodactylus americanus* Weidig, 2010, partial skeleton (holotype USNM 299821). It was collected from the Tynsky Quarry (F2) near Kemmerer, in Lincoln County, Wyoming, United States of America.

**Phylogenetic placement justification:**  *Eozygodactylus americanus* belongs to the extinct Zygodactylidae, which is considered to be the sister group to crown Passeriformes. This sister relationship and monophyly of Zygodactylidae were supported in phylogenetic analyses of morphological data only and in analyses that enforced a molecular constraint (e.g., Mayr 2008, 2015, 2020; Smith et al. 2018).

*Eozygodactylus americanus* shares four apomorphies with the clade formed by *Primozygodactylus* and *Zygodactylus* (Weidig 2010). A phylogenetic analysis including the holotype supports its inclusion in Zygodactylidae (64% bootstrap support), and strongly supports a sister relationship between *E. americanus* and *Zygodactylus grandei* (87% bootstrap support; Smith et al. 2018)*.* Additional material of *E. americanus* will help to determine if *Eozygodactylus* is a junior synonym of *Zygodactylus* (Smith et al. 2018).

**Minimum age**: 51.81 Ma

**Minimum age justification:** The holotype derives from the Fossil Butte Member of the early Eocene (Ypresian) Green River Formation. Radiometric ^40^Ar/^39^Ar dating of the potassium feldspar rich tuff overlying the member yielded an estimated age of 51.97 ± 0.16 Ma (Smith et al. 2010). We use 51.81 Ma as a minimum age for the fossil.

**First occurrences:**

- *Eozygodactylus americanus* Weidig, 2010 (Zygodactylidae), partial articulated skeleton from the Fossil Butte Member of the Green River Formation (Early Eocene, 52.13–51.81 Ma), in Wyoming, USA.
- *Primozygodactylus danielsi* Mayr, 1998, complete articulated skeleton from Messel (early Eocene, 48.25–47.41 Ma), Hessen, Germany.
- *Namapsitta praeruptorum* Mourer-Chauvire *et al.*, 2015 (Psittaciformes?), distal tarsometatarsus from Eocliff (Middle Eocene, Bartonian, 41.03–37.71 Ma), Namibia.
- *Nelepsittacus minimus* Worthy et al., 2011 (Nestoridae), isolated long bones from the Bannockburn Formation, St Bathans Fauna (early Miocene, Altonian, 18.26–15.97 Ma), Otago, New Zealand. Family assignment based on putative apomorphies (Worthy et al. 2011).
- *Cacatua* sp. (Cacatuidae), incomplete rostrum from RSO site (radiometrically dated at 16.86–16.24 Ma, Woodhead *et al.* 2016), Riversleigh, Australia (Boles 1993). The fossil’s rostral morphology is “as modern as any extant member of the genus” (Boles 1993).
- Distal tarsometatarsus of an indeterminate “psittacoid” from the Khalagay Formation (late Early Miocene 13.7–11.6 Ma) at Olkhon island, Russia (Zelenkov 2016).
- cf. Tyrannidae, proximal right ulna (GHUNLPam 19865/15), from the Cerro Azul Formation at Caleufú (late Miocene, Huayquerian, 8.7–6.8 Ma), La Pampa, Argentina (Cenizo et al. 2011).
- *Coracopsis vasa* (Psittacidae), from Antsirabe (latest Pleistocene-Holocene, 0.020) Madagascar (Goodman 1999).

**Fossil record remarks:**

**Clade age estimation:**

One-sample Kolmogorov-Smirnov test

data: Mages

D = 0.32767, p-value = 0.2886

alternative hypothesis: two-sided

Quantiles:

0% 50% 95% 97.5%

51.84 56.68 75.30 81.98

Parameters of the skewStudent function:

offset xi omega alpha nu

51.841 51.917 6.263 131.552 2.380


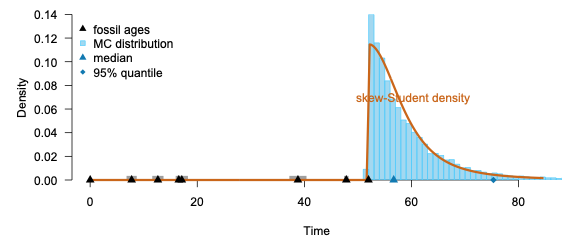


**References:**

Boles, W. E. 1993. A new cockatoo (Psittaciformes: Cacatuidae) from the Tertiary of Riversleigh, northwestern Queensland, and an evaluation of rostral characters in the systematics of parrots. Ibis 135:8-18.

Cenizo, M. M., C. P. Tambussi & C. I. Montalvo 2012. Late Miocene continental birds from the Cerro Azul Formation in the Pampean region (central-southern Argentina). Alcheringa 36(1), 47-68.

Goodman, S.M. 1999. Holocene bird subfossils from the sites of Ampasambazimba, Antsirabe and Ampoza, Madagascar: Changes in the avifauna of south central Madagascar over the past few millennia. In: Adams, N.J. & Slotow, R.H. (eds) Proc. 22 Int. Ornithol. Congr., Durban: 3071-3083. Johannesburg: BirdLife South Africa.

Mayr G. 1998. “Coraciiforme” und “piciforme” Kleinvögel aus dem Mittel- Eozän der Grube Messel (Hessen, Deutschland). Cour Forsch-Inst Senckenberg. 205:1–101.

Mayr, G. 2008. Phylogenetic affinities of the enigmatic avian taxon Zygodactylus based on new material from the early Oligocene of France. Journal of Systematic Palaeontology, 6, 333–344.

Mayr, G., 2015. A reassessment of Eocene parrotlike fossils indicates a previously undetected radiation of zygodactyl stem group representatives of passerines (Passeriformes). Zoologica Scripta 44(6):587-602.

Mayr, G. 2020. A remarkably complete skeleton from the London Clay provides insights into the morphology and diversity of early Eocene zygodactyl near-passerine birds. Journal of Systematic Palaeontology, 18, 1891–1906.

Mourer-Chauvire, C., M. Pickford & B. Senut. 2015. Stem group galliform and stem group psittaciform birds (Aves, Galliformes, Paraortygidae, and Psittaciformes, family incertae sedis) from the Middle Eocene of Namibia. Journal of Ornithology 156:275-286.

Smith, M. E., Chamberlain, K. R., Singer, B. S., Carroll, A. R. 2010. Eocene clocks agree: Coeval 40Ar/39Ar, U-Pb, and astronomical ages from the Green River Formation. Geology, 38, 527–530.

Smith, N. A., DeBee, A. M., & Clarke, J. A. 2018. Systematics and phylogeny of the Zygodactylidae (Aves, Neognathae) with description of a new species from the early Eocene of Wyoming, USA. PeerJ, 6, e4950.

Weidig, I. 2010. New Birds from the Lower Eocene Green River Formation, North America. Records of the Australian Museum 62: 29–44.

Woodhead, J., S. J. Hand, M. Archer, I. Graham, K. Sniderman, D. A. Arena, K. H. Black, H. Godthelp, P. Creaser & Price, E. (2016). Developing a radiometrically-dated chronologic sequence for Neogene biotic change in Australia, from the Riversleigh World Heritage Area of Queensland. Gondwana Research 29(1):153-167.

Worthy, T. H., A. J. D. Tennyson & R. P. Scofield. 2011. An early Miocene diversity of parrots (Aves, Strigopidae, Nestorinae) from New Zealand. Journal of Vertebrate Paleontology 31:1102-1116.

Zelenkov, N. 2016. The first fossil parrot (Aves, Psittaciformes) from Siberia and its implications for the historical biogeography of Psittaciformes. Biology letters 12(10):20160717

**Calibration: crown Eupasseres**

**Category: A**

**MRCA of:** Tyranni and Passeri

**Clade definition in tree:** *Tyrannus savana, Passer domesticus*

**Oldest fossil:** *Wieslochia weissi* Mayr & Manegold, 2006, dissociated nearly complete skeleton in two slabs from the Bott-Eder GmbH clay pit (Rauenberg clay pits) near Frauenweiler, south of Wiesloch, Baden−Württemberg, Germany.

**Phylogenetic placement justification:** Affinities of *Wieslochia* with the suborder Tyranni are indicated by the presence of a well-developed *processus procoracoideus* of the coracoid and a well-developed *tuberculum ligamenti collateralis ventralis* of the ulna (Mayr & Manegold 2004, 2006, Claramunt & Cracraft 2015), and additional putative synapomorphies suggest affinities within the Eurylaimidae (Stervander *et al.* 2020). However, other characters suggest a more basal position in the Passeriformes (Mayr & Manegold 2006). A cladistic analysis of stem Passeriformes suggested a position as a stem Tyranni (Ksepka *et al.* 2019), although this relationship was only supported in the majority rule consensus tree (70%) and taxon sampling for crown Passeriformes was limited to six species.

**Minimum age**: 30.2 Ma

**Minimum age justification:** Biostratigraphic correlations of calcareous nannofossils and dinoflagellate cysts place the youngest Hochberg Member present in Rauenberg in the intersection of NP23 and Subzone D14a (Maxwell *et al.* 2016), indicating an age between 30.2 and 32.0 Ma (Speijer *et al.* 2020).

**First occurrences:**

- *Wieslochia weissi* Mayr & Manegold, 2006, dissociated nearly complete skeleton from the Rauenberg clay pits (early Oligocene, Rupelian, 32–30.2 Ma) near Frauenweiler, south of Wiesloch, Baden−Württemberg, Germany.
- *Orthonyx kaldowinyeri* Boles, 1993, right tarsometatarsus (QM F56329) from Neville’s Garden Site (18.53–17.72 Ma, Woodhead *et al.* 2016), Riversleigh, Australia (Nguyen *et al.* 2014).
- Artamidae indet, proximal scapula fragment (NMNZ S41061) from the Bannockburn Formation (early Miocene, Altonian, 18.26–15.97 Ma) in Otago, New Zealand (Worthy *et al.* 2007).
- Corvides indet., partial skeleton impression from the Fuganji Member of the Iwami Formation (17.6–16.0 Ma, Micklich *et al.* 2017), Miyanoshita, Japan (Kakegawa & Hirao 2003)
- Passeriformes indet., fragmentary distal humerus (Kenyan National Museum-SO 5466) from the Early Miocene (23.04 - 15.99 Ma) of Songhor, Kenya (Mayr 2014).
- *Miocitta galbreathi* Brodkorb, 1972 from the middle Miocene (Barstovian, 14.78–14.07 Ma) Pawnee Creek Formation in Logan County, Colorado, USA.
- cf. Tyrannidae, proximal right ulna (GHUNLPam 19865/15) from the Cerro Azul Formation at Caleufú, La Pampa, Argentina (Cenizo et al. 2012). This formation is Huayquerian, possibly extending earlier to Chasicoan, and Caleufú is associated with the rodent *Xenodontomys* *elongatus* biozone (late Huayquerian). The estimated age of this fossil would be 8.7–6.8 Ma, based on Huayquerian boundaries (Hilgen et al. 2012).
- *Phedina borbonica, Corvus albus*, *Ploceus sakalava* remains from Ankilitelo Cave (0.0112–0.005 Ma) Madagascar (Goodman et al. 2013).

**Fossil record remarks:**

**Clade age estimation:**

One-sample Kolmogorov-Smirnov test

data: Mages

D = 0.22691, p-value = 0.726

alternative hypothesis: two-sided

Quantiles:

0% 50% 95% 97.5%

30.23 33.98 45.73 49.90

Parameters of the skewStudent function:

offset xi omega alpha nu

30.233 30.841 4.250 12.623 2.737


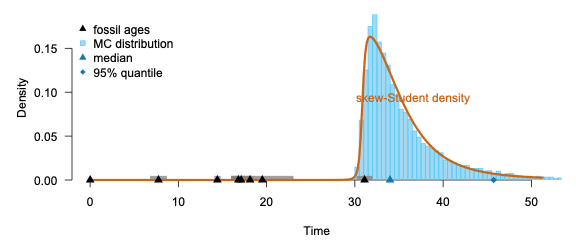


**References:**

Boles, W. E. 1993. A logrunner *Orthonyx* (Passeriformes, Orthonychidae) from the Miocene of Riversleigh, north-western Queensland. Emu, 93(1), 44-49.

Brodkorb, P. 1972. Neogene fossil jays from the Great Plains. Condor 347-349.

Cenizo, M. M., C. P. Tambussi & C. I. Montalvo 2012. Late Miocene continental birds from the Cerro Azul Formation in the Pampean region (central-southern Argentina). Alcheringa 36(1), 47-68.

Claramunt, S., & J. Cracraft, J. (2015). A new time tree reveals Earth history’s imprint on the evolution of modern birds. Science Advances 1(11):e1501005.

Goodman, S.M., Raherilalao, M.J. and Muldoon, K., 2013. Bird fossils from Ankilitelo Cave: inference about Holocene environmental changes in southwestern Madagascar. Zootaxa 3750(5):534-548.

Kakegawa, Y. & K. Hirao 2003 A Miocene passeriform bird from the Iwami Formation, Tottori Group, Tottori, Japan. Bulletin of the National Science Museum, Series C 29:33–37.

Ksepka, D.T., L. Grande & G. Mayr 2019. Oldest finch-beaked birds reveal parallel ecological radiations in the earliest evolution of passerines. Current Biology 29(4):657-663.

Mayr, G. 2014. On the middle Miocene avifauna of Maboko Island, Kenya. Geobios 47(3):133-146.

Mayr, G., & A. Manegold. 2004. The oldest European fossil songbird from the early Oligocene of Germany. Naturwissenschaften 91:173–177.

Mayr, G., & A. Manegold. 2006. New specimens of the earliest European passeriform bird. Acta Palaeontologica Polonica 51:315–323.

Maxwell, E. E., Alexander, S., Bechly, G., Eck, K., Frey, E., Grimm, K., Kovar-Eder, J., Mayr, G., Micklich, N., Rasser, M. and Roth-Nebelsick, A. 2016. The Rauenberg fossil Lagerstätte (Baden-Württemberg, Germany): A window into early Oligocene marine and coastal ecosystems of Central Europe. Palaeogeography, Palaeoclimatology, Palaeoecology 463:238-260.

Micklich, N., Bannikov, A. F., & Yabumoto, Y. (2017). First record of ponyfishes (Perciformes: Leiognathidae) from the Oligocene of the Grube Unterfeld (“Frauenweiler”) clay pit. PalZ, 91(3), 375-398.

Nguyen, J. M., Boles, W. E., Worthy, T. H., Hand, S. J., & Archer, M. (2014). New specimens of the logrunner *Orthonyx kaldowinyeri* (Passeriformes: Orthonychidae) from the Oligo-Miocene of Australia. Alcheringa 38(2): 245-255.

Speijer, R. P., H. Palike, C.J. Hollis, J.J. Hooker & J.G. Ogg. 2020 The Paleogene period. Pp. 1087-1140 in F. M. Gradstein, J. G. Ogg, M. D. Schmitz & G. M. Ogg (eds.) The geologic time scale. Elsevier.

Stervander M, Fjeldså J, Christidis L, Ericson PGP, Ohlson JI, Alström P. 2020. An updated chronology of passerine birds. In: Fjeldså J, Christidis L & Ericson PGP (eds). The Largest Avian Radiation: The Evolution of Perching Birds, or the Order Passeriformes (pp 387–396). Lynx Edicions, Barcelona.

Worthy, T. H., A. J. D. Tennyson, C. Jones, J. A. McNamara & B. J. Douglas. 2007. Miocene waterfowl and other birds from Central Otago, New Zealand. Journal of Systematic Palaeontology 5:1–39.

**Calibration: Menurides**

**Category: A**

**MRCA of:** Menuridae and Atrichornithidae

**Clade definition in tree:** *Menura novaehollandiae, Atrichornis clamosus*

**Oldest fossil:** *Menura tyawanoides* Boles 1995, left carpometacarpus (QM F20887) from the Riversleigh Faunal Zone B at Upper Site, Riversleigh, Australia.

**Phylogenetic placement justification:** The original description does not explicitly list apomorphies that place *M. tyawanoides* in Menuridae and the fossil has yet to be analysed in a phylogenetic context. From direct examination of the fossil and comparisons with extant taxa, the fossil shows the following diagnostic suite of features for Menuridae: 1) the fovea carpalis caudalis is located well proximally of the level of the processus cranialis; 2) the fossa ligamenti ventralis is very shallow; 3) the processus intermetacarpalis does not protrude beyond the caudal edge of the os metacarpale minus; 4) the distal edge of the trochlea carpalis ventralis is proximally of the level of the distal edge of processus alularis (Nguyen, pers. obs.).

**Minimum age:** 15.99 Ma

**Minimum age justification:** Upper Site is allocated to Faunal Zone B (formerly System B) in Riversleigh biochronology. Based on stratigraphy and biocorrelation of fossil vertebrate local faunas, Faunal Zone B is interpreted to be early Miocene (Aquitanian–Burdigalian) in age (e.g. Woodhead et al. 2016). A biostratigraphic study of Riversleigh mammal faunas allocated Upper Site to faunal interval B3, the youngest subdivision of Faunal Zone B (Arena et al. 2016). We use the upper boundary of the Burdigalian (15.99 Ma in Raffi et al. 2020) as a minimum age for the fossil.

**First occurrences:**

- *Menura tyawanoides* Boles, 1995, left carpometacarpus (QM F20887) from Upper Site (early Miocene, 23.04–15.99 Ma), Riversleigh, Australia (Boles 1995).
- *Atrichornis rufescens*, complete left tarsometatarsus (NMV P183110) and incomplete left tarsometatarsus (NMV P183111) from Pyramids Cave M-89 (Late Pleistocene-Holocene, 0.033–0.0024 Ma), Victoria, Australia (Baird 1993).

**Fossil record remarks:**

Quantiles:

0% 50% 95% 97.5%

16.09 26.77 84.62 115.00

Parameters of the skewStudent function:

offset xi omega alpha nu

16.087 17.537 11.027 14.070 1.538

**
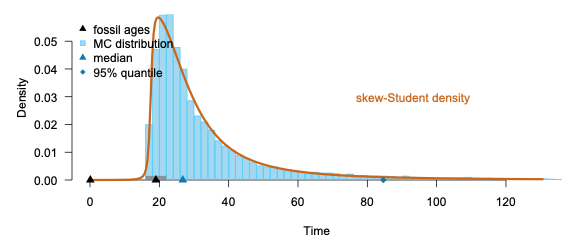
**

**References:**

Arena, D. A., Travouillon, K. J., Beck, R. M. D., Black, K. H., Gillespie, A. K., Myers, T. J., Archer, M., & Hand, S. J. 2016. Mammalian lineages and the biostratigraphy and biochronology of Cenozoic faunas from the Riversleigh World Heritage Area, Australia. Lethaia, 49, 43–60.

Baird, R. F. 1993 Pleistocene avian fossils from Pyramids Cave (M-89), eastern Victoria, Australia. Alcheringa 17(4):383-404.

Boles, W. E. 1995. A preliminary analysis of the Passeriformes from Riversleigh, northwestern Queensland, Australia, with the description of a new species of Lyrebird. Courier Forschungsinstitut Senckenberg 181:163–170.

Raffi, I., Wade, B. S., Pälike, H., Beu, A. G., Cooper, R., Crundwell, M. P., Krijgsman, W., Moore, T., Raine, I., Sardella, R., & Vernyhorova, Y. V. 2020. The Neogene Period. In F. M. Gradstein, J. G. Ogg, M. D. Schmitz, & G. M. Ogg (Eds.), Geologic Time Scale 2020 (pp. 1141–1215). Elsevier B.V.: Amsterdam.

Woodhead, J., S. J. Hand, M. Archer, I. Graham, K. Sniderman, D. A. Arena, K. H. Black, H. Godthelp, P. Creaser & E. Price. 2016. Developing a radiometrically-dated chronologic sequence for Neogene biotic change in Australia, from the Riversleigh World Heritage Area of Queensland. Gondwana Research 29(1):153-167.

**Calibration: Meliphagides minus Maluridae**

**Category: A**

**MRCA of:** Meliphagidae and Dasyornithidae

**Clade definition in tree:** *Dasyornis broadbenti, Pardalotus punctatus*

**Oldest fossil:** *Dasyornis walterbolesi* Nguyen, 2019, associated left femur, right tibiotarsus, and left tarsometatarsus (holotype QM F50580). It derives from Camel Sputum Site, Godthelp Hill in the Riversleigh World Heritage Area, Queensland, Australia.

**Phylogenetic placement justification:** A phylogenetic analysis including the holotype strongly supports the placement of *D. walterbolesi* in Dasyornithidae (93% bootstrap support). High support (98% bootstrap support) for this relationship is retained when a molecular backbone constraint is enforced. The interfamilial relationships of *D. walterbolesi* are less resolved; an analysis that enforced a molecular constraint recovered low bootstrap support (63%) for a sister relationship with *D. broadbenti* (Nguyen 2019). The holotype exhibits a combination of two features that is autapomorphic for Dasyornithidae: 1) a large, prominent bony projection that is fused to the proximomedial corner of the condylus medialis of the femur; 2) a shallow furrow on the distal and plantar surfaces of the trochlea metatarsi II of the tarsometatarsus.

**Minimum age:** 16.97 Ma

**Minimum age justification:** Based on biocorrelation and stratigraphy, Camel Sputum Site is allocated to the early Miocene Faunal Zone B (interval B3) in Riversleigh biochronology (Archer et al. 1989, 1997; Travouillon et al. 2006; Arena et al. 2016). Radiometric U-Pb dating of speleothem within the fossil deposit returned an estimated age of 17.75 ± 0.78 Ma (Woodhead et al. 2016). We use a minimum age of 16.97 Ma for the fossil.

**First occurrences:**

- *Dasyornis walterbolesi* Nguyen, 2019 (Dasyornithidae), associated femur, partial tibiotarsus, and partial tarsometatarsus (holotype QM F50580) from Camel Sputum Site (early Miocene, 18.53–16.97 Ma), Riversleigh, Australia.
- *Philemon* sp., distal tarsometatarsus fragment (LB-Av-740) from Liang Bua (Late Pleistocene, 0.060–0.050 Ma), Flores, Indonesia (Meijer *et al.* 2017).
- *Prosthemadera novaeseelandiae* (Meliphagidae) one specimen from Cliffs at Old Rifle Butts (late Pleistocene, 0.129–0.0117 Ma), Cape Wanbrow, South Island, Otago, New Zealand (Worthy & Grant-Mackie 2003).

**Fossil record remarks:**

One-sample Kolmogorov-Smirnov test

data: Mages

D = 0.66408, p-value = 0.07584

alternative hypothesis: two-sided

Quantiles:

0% 50% 95% 97.5%

17.00 22.24 48.27 60.57

Parameters of the skewStudent function:

offset xi omega alpha nu

16.997 17.523 5.763 19.032 1.656

**
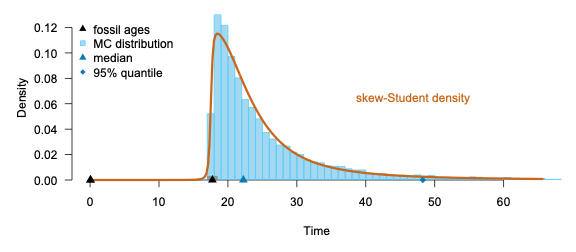
**

**References:**

Archer, M., Godthelp, H., Hand, S. J., & Megirian, D. 1989. Fossil mammals of Riversleigh, northwestern Queensland: Preliminary overview of biostratigraphy, correlation and environmental change. *Australian Zoologist*, *25*, 29–65.

Archer, M., Hand, S. J., Godthelp, H., & Creaser, P. 1997. Correlation of the Cainozoic sediments of the Riversleigh World Heritage fossil property, Queensland, Australia. In J.-P. Aguilar, S. Legendre, & J. Michaux (Eds.), *Actes du congrès BiochroM’97*, *Mémoires et Travaux de l’Ecole Pratique des Hautes Etudes*, *21*, 131–152. Institut de Montpellier: Montpellier.

Arena, D. A., Travouillon, K. J., Beck, R. M. D., Black, K. H., Gillespie, A. K., Myers, T. J., Archer, M., & Hand, S. J. 2016. Mammalian lineages and the biostratigraphy and biochronology of Cenozoic faunas from the Riversleigh World Heritage Area, Australia. *Lethaia, 49*, 43–60.

Meijer, H. J., Due, R. A., Sutikna, T., Saptomo, W., Wasisto, S., Tocheri, M. W., & Mayr, G. (2017). Late Pleistocene songbirds of Liang Bua (Flores, Indonesia); the first fossil passerine fauna described from Wallacea. PeerJ 5:e3676.

Nguyen, J. M. 2019. A new species of bristlebird (Passeriformes, Dasyornithidae) from the early Miocene of Australia. Journal of Vertebrate Paleontology, e1575838.

Travouillon, K. J., Archer, M., Hand, S. J., & Godthelp, H. 2006. Multivariate analyses of Cenozoic mammalian faunas from Riversleigh, northwestern Queensland. *Alcheringa*, *30*(S1), 323–349.

Woodhead, J., S. J. Hand, M. Archer, I. Graham, K. Sniderman, D. A. Arena, K. H. Black, H. Godthelp, P. Creaser & E. Price. 2016. Developing a radiometrically-dated chronologic sequence for Neogene biotic change in Australia, from the Riversleigh World Heritage Area of Queensland. Gondwana Research 29(1):153-167.

Worthy, T. H., & J. A. Grant-Mackie. 2003. Late-Pleistocene avifaunas from Cape Wanbrow, Otago, South Island, New Zealand. Journal of the Royal Society of New Zealand 33:427-485.

**Calibration: Orthonychides**

**Category: A**

**MRCA of**: Orthonychidae and Pomatostomidae

**Clade definition in tree:** *Orthonyx spaldingii, Pomatostomus ruficeps*

**Oldest fossil*:*** *Orthonyx kaldowinyeri* Boles, 1993, right tarsometatarsus (QM F56329) (Nguyen et al. 2014). The fossil was collected from Neville’s Garden Site in the Riversleigh World Heritage Area, Queensland, Australia.

**Phylogenetic placement justification:** The fossil exhibits two autapomorphies of Orthonychidae: 1) the impressio ligamenti collateralis lateralis is low and very large; 2) the plantar surface immediately proximal to the incisura intertrochlearis medialis is very shallowly excavated, whereas the plantar surface immediately proximal to the lateral incisura is deeply excavated (Nguyen et al. 2014).

**Minimum age:** 17.72 Ma

**Minimum age justification:** Neville’s Garden Site is assigned to Riversleigh Faunal Zone B (interval B3), which is considered to be early Miocene in age based on biocorrelation and stratigraphy (Archer *et al.* 1989, 1997; Travouillon et al. 2006; Arena et al. 2016). Radiometric U-Pb dating of two samples of speleothems in the deposit, including one contemporaneous with fossils, obtained estimated ages of 18.24 ± 0.29 Ma and 17.85 ± 0.13 Ma (Woodhead *et al.* 2016). We use 17.72 Ma as a minimum age for QM F56329.

A distal end of a tarsometatarsus (QM F30244) referred to *O. kaldowinyeri* was collected from Hiatus Site, which is assigned to the older Faunal Zone A (formerly System A) in Riversleigh biochronology. This faunal zone is regarded as late Oligocene in age based on biocorrelation of vertebrate assemblages with those of the Etadunna Formation (Archer et al. 1989). However, Arena et al.’s (2016) biostratigraphic analysis could not resolve the relative position of Hiatus Site in the Riversleigh sequence because of insufficient representation of lineage taxa. Based on the analysis results, Hiatus Site could be allocated to Faunal Zones A (late Oligocene), B (early Miocene), or C (middle Miocene). Because the temporal relationships of Hiatus Site within the Riversleigh biochronology are unresolved, we selected QM F56329 from the radiometrically dated Neville’s Garden Site as a fossil calibration.

**First occurrences:**

- *Orthonyx kaldowinyeri* Boles, 1993, distal right tarsometatarsus (QM F30244) from Neville’s Garden Site (early Miocene, 18.53–17.72 Ma, Woodhead *et al.* 2016), Riversleigh, Australia (Nguyen *et al.* 2014).
- *Pomatostomus* sp., multiple bones from Rackham’s Roost Site (2.69– 1.10 Ma), Riversleigh, Australia (Nguyen *et al.* 2016).
- *Orthonyx hypsilophus* Baird, 1985, incomplete pelvis (NMV P24444) from the middle or late Pleistocene (0.774–0.0117 Ma, Baird 1985, Shute et al. 2016) of Green Waterhole Cave (=Fossil Cave), Victoria, Australia.
- *Orthonyx wakefieldi* Baird, 1993, incomplete right femur (NMV P183118), incomplete right humerus (NMV P183112), and multiple other referred bones from the Late Pleistocene (0.040 – 0.0024 Ma) of Pyramids Cave M-89, Victoria, Australia.

**Fossil record remarks:** Fossil record not uniform (One-sample Kolmogorov-Smirnov test *D* = 0.63, *P* = 0.046). Given that the two oldest fossils belong to different subclades, we used the ghost-lineage method (Norris et al. 2015).

**Clade age estimation:**

Quantiles:

0% 50% 95% 97.5%

17.76 26.25 173.40 322.50

Parameters of the skewStudent function:

offset xi omega alpha nu

17.758 17.920 7.171 109.573 0.788


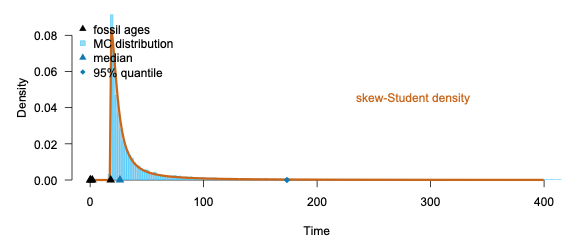


**References:**

Archer, M., Godthelp, H., Hand, S. J., & Megirian, D. 1989. Fossil mammals of Riversleigh, northwestern Queensland: Preliminary overview of biostratigraphy, correlation and environmental change. Australian Zoologist, 25, 29–65.

Archer, M., Hand, S. J., Godthelp, H., & Creaser, P. 1997. Correlation of the Cainozoic sediments of the Riversleigh World Heritage fossil property, Queensland, Australia. In J.-P. Aguilar, S. Legendre, & J. Michaux (Eds.), Actes du congrès BiochroM’97, Mémoires et Travaux de l’Ecole Pratique des Hautes Etudes, 21, 131–152. Institut de Montpellier: Montpellier.

Arena, D. A., Travouillon, K. J., Beck, R. M. D., Black, K. H., Gillespie, A. K., Myers, T. J., Archer, M., & Hand, S. J. 2016. Mammalian lineages and the biostratigraphy and biochronology of Cenozoic faunas from the Riversleigh World Heritage Area, Australia. Lethaia, 49, 43–60.

Boles, W. E. 1993. A logrunner *Orthonyx* (Passeriformes: Orthonychidae) from the Miocene of Riversleigh, north-western Queensland. Emu 93: 44–49.

Baird, R. F. 1993 Pleistocene avian fossils from Pyramids Cave (M-89), eastern Victoria, Australia. Alcheringa 17(4):383-404.

Baird, R. F. 1985 Avian Fossils from Quaternary Deposits in 'Green Waterhole Cave', South-eastern South Australia. Records of the Australian Museum 37: 353-370.

Nguyen, J. M.T.,W. E. Boles,T. H. Worthy, S. J. Hand & M. Archer 2014. New specimens of the logrunner *Orthonyx kaldowinyeri* (Passeriformes: Orthonychidae) from the Oligo-Miocene of Australia. Alcheringa 38:245-255.

Nguyen, J. M. T., Hand, S. J., & Archer, M. 2016. The Late Cenozoic Passerine Avifauna from Rackham’s Roost Site, Riversleigh, Australia. Records of the Australian Museum 68(5):201-230.

Shute, E., Prideaux, G.J., Worthy, T.H. 2016. Three terrestrial Pleistocene coucals (*Centropus*: Cuculidae) from southern Australia: biogeographical and ecological significance, *Zoological Journal of the Linnean Society*, 177, 964–1002.

Travouillon, K. J., Archer, M., Hand, S. J., & Godthelp, H. 2006. Multivariate analyses of Cenozoic mammalian faunas from Riversleigh, northwestern Queensland. Alcheringa 30(S1):323–349.

Woodhead, J., S. J. Hand, M. Archer, I. Graham, K. Sniderman, D. A. Arena, K. H. Black, H. Godthelp, P. Creaser & E. Price. 2016. Developing a radiometrically-dated chronologic sequence for Neogene biotic change in Australia, from the Riversleigh World Heritage Area of Queensland. Gondwana Research 29(1):153-167.

**Calibration: part Malaconotoidea (minus Machaerirhynchidae)**

**Category: A**

**MRCA of:** Malaconotoidea minus Machaerirhynchidae

**Clade definition in tree:** *Gymnorhina tibicen*, *Mystacornis crossleyi*

**Oldest fossil:** *Kurrartapu johnnguyeni* Nguyen, 2013, a proximal right tarsometatarsus (holotype QM F56251) from Price is Right Site, Riversleigh, Queensland, Australia (Nguyen *et al.* 2013)

**Phylogenetic placement justification:** *Kurrartapu johnnguyeni* shows derived states of Cracticinae that excludes it from *Peltops* and *Artamus* (Nguyen et al. 2013). The proximal scapula (NMNZ S41061) from the Altonian (18.26–15.97 Ma) St Bathans Fauna in Otago, New Zealand, shows a pneumatic foramen like cracticines, but *Peltops* and even several specimens of *Artamus* also show a pneumatic foramen (Nguyen pers. com., contra Worthy *et al.* 2007). Therefore, NMNZ S41061 cannot be unequivocally assigned to crown Cracticinae but can also be in the stem or even in Artamidae, although its size seems to be well above the size of extant Artamidae.

**Minimum age:** 14.8 Ma

**Minimum age justification:** Based on stratigraphy and biocorrelation, Price is Right Site is allocated to Riversleigh Faunal Zone B and is regarded as early Miocene in age (Archer et al. 1989, 1997; Travouillon et al. 2006). A biostratigraphic analysis by Arena et al. (2016) could not resolve the relative position of Price is Right Site within the Riversleigh sequence due to insufficient representation of lineage taxa at the site. The analysis results suggested that this site could be part of upper Faunal Zone B (intervals B2 or B3) or lower Faunal Zone C (interval C1), which is middle Miocene in age (Arena et al. 2016). Taking into account this uncertainty, we use the upper boundary of lower Faunal Zone C (14.8 Ma, from Figure 6 in Woodhead et al. 2016) as a minimum age for the fossil.

**First occurrences:**

- *Kurrartapu johnnguyeni* Nguyen, 2013, a proximal right tarsometatarsus (holotype QM F56251) from Price is Right Site (23.04–14.8 Ma), Riversleigh, Queensland, Australia.
- Artamidae indet., proximal scapula fragment (NMNZ S41061) from the Bannockburn Formation at Croc Site L1 (Altonian, 18.26 – 15.97 Ma), near St Bathans, Otago, New Zealand (Worthy et al. 2007).

**Fossil record remarks:**

Quantiles:

0% 50% 95% 97.5%

16.00 19.52 27.84 32.32

Parameters of the skewStudent function:

offset xi omega alpha nu

15.997 16.614 3.741 9.209 3.031

**
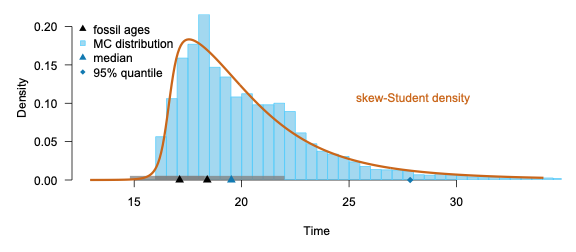
**

**References:**

Archer, M., Godthelp, H., Hand, S. J., & Megirian, D. 1989. Fossil mammals of Riversleigh, northwestern Queensland: Preliminary overview of biostratigraphy, correlation and environmental change. Australian Zoologist, 25, 29–65.

Archer, M., Hand, S. J., Godthelp, H., & Creaser, P. 1997. Correlation of the Cainozoic sediments of the Riversleigh World Heritage fossil property, Queensland, Australia. In J.-P. Aguilar, S. Legendre, & J. Michaux (Eds.), Actes du congrès BiochroM’97, Mémoires et Travaux de l’Ecole Pratique des Hautes Etudes, 21, 131–152. Institut de Montpellier: Montpellier.

Arena, D. A., Travouillon, K. J., Beck, R. M. D., Black, K. H., Gillespie, A. K., Myers, T. J., Archer, M., & Hand, S. J. 2016. Mammalian lineages and the biostratigraphy and biochronology of Cenozoic faunas from the Riversleigh World Heritage Area, Australia. Lethaia, 49, 43–60.

Nguyen, J. M.T., T. H. Worthy, W. E. Boles, S. J. Hand & M. Archer 2013 A new cracticid (Passeriformes : Cracticidae) from the Early Miocene of Australia. Emu 113:374-382.

Travouillon, K. J., Archer, M., Hand, S. J., & Godthelp, H. 2006. Multivariate analyses of Cenozoic mammalian faunas from Riversleigh, northwestern Queensland. Alcheringa, 30(S1), 323–349.

Worthy, T. H., A. J. D. Tennyson, C. Jones, J. A. McNamara, and B. J. Douglas. 2007. Miocene waterfowl and other birds from Central Otago, New Zealand. Journal of Systematic Palaeontology 5:1–39.

**Calibration: Certhioidea + Muscicapoidea**

**Category: B**

**MRCA of:** Certhioidea and Muscicapoidea

**Clade definition in tree:** *Certhia familiaris, Erithacus rubecula*

**Oldest fossil:** *Certhiops rummeli* Manegold, 2008, right tarsometatarsus (holotype NMA 2007/51/2021) from ‘Petersbuch 62’, near Petersbuch, north of Eichstatt, Bavaria, Germany.

**Phylogenetic placement justification:** *Certhiops rummeli* was described as a member of the “climbing Certhioidea”, a group comprising Sittidae and Certhiidae (Manegold 2008). The fossil shares the following suite of derived features with scansorial members of Certhioidea: 1) the crista lateralis hypotarsi extends farther proximally than the crista medialis hypotarsi; 2) a characteristic arrangement of hypotarsal canals; 3) hypotarsal canal for fp3 and fp4 tendons is is plantarly open; 4) the medial edge of the shaft, distally of the fossa metatarsi I, bears a protrusion (except in *Tichodroma*; 5) trochleae metatarsorum are short and widely separated; 6) the trochlea metatarsi III is large, protrudes both dorsally and plantarly, and 7) bears a deep furrow.

Recent molecular studies have shown, however, that Sittidae and Certhiidae do not form a monophyletic clade (e.g., Claramunt & Cracraft 2015; Oliveros et al. 2019; this study). We therefore regard the relationships of *C. rummeli* within Certhioidea uncertain and use this fossil to constrain the minimum age of the most recent common ancestor of Certhioidea and its sister group, the Muscicapoidea.

**Minimum age:** 17.2 Ma

**Minimum age justification:** The Petersbuch 62 site is correlated with European Mammal Neogene Zone 3 (MN3, early Miocene) based on the occurrence of specific faunal elements, such as the mammal *Galerix aurelianensis*. The predator fauna and absence of cricetid rodent taxa characteristic of MN4 suggests that this site is of similar age to Wintershof West, which is assigned to MN3 (Rosina & Rummel 2012; Goelich 2017). The MN3 zone has a lower boundary of ~19.5 Ma and an upper boundary of 17.2 Ma (Hilgen et al. 2012); we therefore use 17.2 Ma as a minimum age for the fossil.

**First occurrences:**

- *Certhiops rummeli* Manegold, 2008 (Certhioidea), right tarsometatarsus (NMA 2007/51/2021) from Petersbuch 62 (early Miocene, MN3, 19.5–17.2 Ma), near Petersbuch, north of Eichstatt, Bavaria, Germany.
- *Mimus polyglottos* premaxilla (UM 52094) from the Rexroad Formation (early Blancan, 4.8–4.3 Ma) at Fox Canyon, Kansas, USA (Emslie 2007).
- Sturnidae gen. et sp. indet., fossils from the upper Varswater Formation at Langebaanweg (Early Pliocene, 5.33–3.6 Ma), Cape Province, South Africa (Manegold et al. 2013).
- *Sitta* sp. (Sittidae) distal part of right tarsometatarsus (PIN 2975/162), *Turdus* (Turdidae) three partial tarsometatarsi (PIN 2975/169, 170, 171), and *Saxicola* (Muscicapidae) four distal tarsometatarsi (PIN 2975/164, 165, 166, 167), from the Early Villafranchian (late Pliocene, MN16b, 3.6–2.588 Ma) at Beregovaya, Transbaikalia, Republic of Buryatia, Russia (Zelenkov & Kurochkin 2012).
- *Mimus longicaudatus* from the Talara Tar Seeps (Late Pleistocene, 0.018–0.015 Ma), Piura, Peru (Oswald & Steadman 2015).

**Fossil record remarks:**

**Clade age estimation:** Kolmogorov-Smirnov test: D = 0.5837, p-value = 0.0378

Quantiles:

0% 50% 95% 97.5%

17.23 25.21 151.70 292.30

Parameters of the skewStudent function:

offset xi omega alpha nu

17.2348 17.8318 6.7350 24.0828 0.8304

**
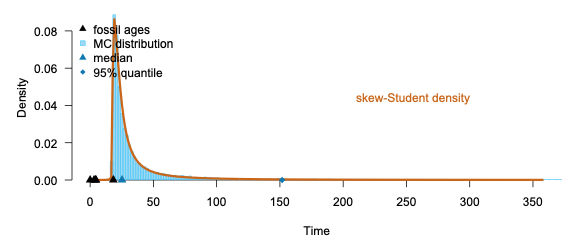
**

**References:**

Claramunt, S., & Cracraft, J. 2015. A new time tree reveals Earth history’s imprint on the evolution of modern birds. Science Advances, 1(11), e1501005.

Emslie, S. D. (2007). Fossil passerines from the early Pliocene of Kansas and the evolution of songbirds in North America. *The Auk*, *124*(1), 85-95.

Goelich, U. B. 2017. Catalogue of the fossil bird holdings of the Bavarian State Collection of Palaeontology and Geology in Munich. Zitteliana, 89, 331–349.

Hilgen, F. J., Lourens, L. J., Van Dam, J. A., Beu, A. G., Boyes, A. F., Cooper, R. A., Krijgsman, W., Ogg, J. G., Piller, W. E., & Wilson, D. S. 2012. The Neogene Period. In F. M. Gradstein, J. G. Ogg, M. D. Schmitz, & G. M. Ogg (Eds.), The Geologic Time Scale 2012 (pp. 923–978). Elsevier B.V.: Oxford.

Manegold, A. 2008. Earliest fossil record of the Certhioidea (treecreepers and allies) from the early Miocene of Germany. Journal of Ornithology 149:223-228.

Manegold, A., A. Louchart, J. Carrier & A. Elzanowski 2013 The early Pliocene avifauna of Langebaanweg (South Africa): a review and update. In Paleornithological Research 2013: Proceedings of the 8th International Meeting of the Society of Avian Paleontology and Evolution pp. 135-152.

Oliveros, C. H., Field, D. J., Ksepka, D. T., Barker, F. K., Aleixo, A., Andersen, M. J., Alström, P., Benz, B. W., Braun, E. L., Braun, M. J., Bravo, G. A., Brumfield, R. T., Chesser, R. T., Claramunt, S., Cracraft, J., Cuervo, A. M., Derryberry, E. P., Glenn, T. C., Harvey, M. G., Hosner, P. A., Joseph, L., Kimball, R. T., Mack, A. L., Miskelly, C. M., Peterson, A. T., Robbins, M. B., Sheldon, F. H., Silveira, L. F., Smith, B. T., White, N. D., Moyle, R. G., & Faircloth, B. C. Earth history and the passerine superradiation. *Proceedings of the National Academy of Sciences*, *116*, 7916–7925

Oswald, J. A., & Steadman, D. W. 2015. The changing diversity and distribution of dry forest passerine birds in northwestern Peru since the last ice age. The Auk 132(4):836-862.

Rosina, V.V. & Rummel, M. 2012. The bats (Chiroptera, Mammalia) from the Early Miocene of Petersbuch (Bavaria, Southern Germany). Geobios, 45, 463–478.

Zelenkov, N. V., & E. N. Kurochkin. 2012. The first representative Pliocene assemblages of passerine birds in Asia (Northern Mongolia and Russian Transbaikalia). Geobios 45:323-334.

**Calibration: Passerellidae and allies**

**Category: A**

**MRCA of:** Passerellidae and Icteridae + Parulidae

**Clade definition in tree:** *Zonotrichia albicollis, Molothrus ater*

**Oldest fossil:** *Palaeostruthus* *hatcheri* (Shufeldt, 1913), rostrum maxillae (holotype USNM 6647). It was collected from "Quarry E" near Long Island in Phillips County, Kansas, United States of America.

**Phylogenetic placement justification:** Shufeldt (1913) provisionally referred the fossil to the extinct genus *Palaeospiza*, which was previously in Passeriformes but it is now in Coliiformes (Mayr 2001; Ksepka & Clarke 2009). Wetmore (1925) erected a new genus for the fossil, *Palaeostruthus*, and proposed affinities with *Pipilo* (Passerellidae). Steadman (1981) noted that the fossil is very similar to the extant sparrow *Ammodramus savannarum* and considered *Palaeostruthus* to be a junior synonym of *Ammodramus.* We agree that *Palaeostruthus* shows the typical rostrum of Passerellidae (different from that of Emberizidae and Calcariidae), but consider the similarity with *Ammodramus savannarum* superficial, as the palatal surface of the rostrum extends more caudally than in any extant species of *Ammodramus* (Claramunt pers. obs.). Therefore, because relationships within the family are better considered uncertain, we applied the calibration the time of origin (stem age) of Passerellidae, which corresponds to the MRCA of Passerellidae, Icteridae and Parulidae.

**Minimum age:** 7.5 Ma

**Minimum age justification:** The holotype is from the Barstovian–Hemphillian (Miocene-Pliocene) Ogallala Formation. The deposits at Long Island quarry are early Hemphillian (Hh1; late Miocene) (Janis et al. 2008). This gives an estimated age of 9.0–7.5 Ma for the fossil, based on Hh1 boundaries in Hilgen et al. (2012).

**First occurrences:**

- *Palaeostruthus hatcheri* (Shufeldt, 1913), complete rostrum (USNM 6647) from Quarry E (late Miocene, 9.0 –7.5 Ma) near Long Island, Phillips County, Kansas.
- *Zonotrichia robusta* Tonni, 1970, fragmentary beak from the Ensenadan (early Pleistocene, 1.2–0.781 Ma) of S.E. Buenos Aires Province, Argentina.

**Fossil record remarks:**

Quantiles:

0% 50% 95% 97.5%

7.534 11.370 34.540 49.900

Parameters of the skewStudent function:

offset xi omega alpha nu

7.534 7.948 3.953 17.621 1.418


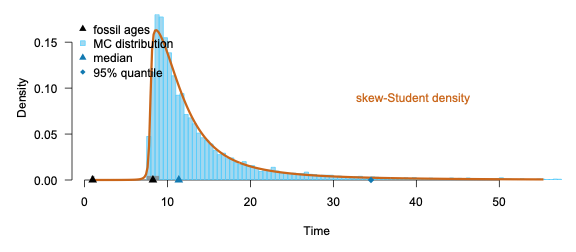


**References:**

Hilgen, F. J., Lourens, L. J., Van Dam, J. A., Beu, A. G., Boyes, A. F., Cooper, R. A., Krijgsman, W., Ogg, J. G., Piller, W. E., & Wilson, D. S. 2012. The Neogene Period. In F. M. Gradstein, J. G. Ogg, M. D. Schmitz, & G. M. Ogg (Eds.), The Geologic Time Scale 2012 (pp. 923–978). Elsevier, Oxford.

Janis C, Gunnell G, Uhen M 2008. Evolution of Tertiary mammals of North America. Volume 2:

Small mammals, Xenarthrans, and Marine mammals. Cambridge University Press, Cambridge.

Ksepka, D. T., & Clarke, J. A. 2009. Affinities of *Palaeospiza bella* and the phylogeny and biogeography of mousebirds (Coliiformes). Auk 126:245–259.

Mayr, G. 2001. New specimens of the Middle Eocene fossil mousebird Selmes absurdipes Peters, 1999. Ibis, 143, 427–434.

Steadman, D. W. 1981. A re-examination of *Palaeostruthus hatcheri* (Shufeldt), a late Miocene sparrow from Kansas. Journal of Vertebrate Paleontology 1:171-173.

Shufeldt, R. W. 1913. Further studies of fossil birds with descriptions of new and extinct species. Bulletin of the American Museum of Natural History 32:285-306.

Tonni, E. P. 1970. *Zonotrichia robusta* n. sp. (Aves, Passeriformes) del Pleistoceno Medio de Buenos Aires. Ameghiniana 11:366-372.

Wetmore, A. 1925. The systematic position of *Palaeospiza bella* Allen, with observations on other fossil birds. Bulletin of the Museum of Comparative Zoology 67:183-193.

#

#

# **Supplementary Methods**

##

We extracted different genomic data types with different workflows, which are described in detail in the following sections. The genome-wide windows and the intronic regions were extracted from the Cactus whole genome alignment [^1^](https://paperpile.com/c/QHZ1Z3/mJFTa). The genome-wide windows were further cleaned from any overlap with exons or introns to obtain intergenic regions. The exonic and UCE loci were extracted using separate pipelines as described below.

For all loci irrespective of data type, we employed the same minimal filtering prior to alignment for exons and UCEs, or prior to gene tree analysis for intergenic regions and introns.

1. We excluded fragmentary sequences, i.e. sequences shorter than 50% of the median length of all sequences of the locus because these fragmentary sequences can impact alignment accuracy and contain fewer parsimony informative sites than the remaining sequences. For the intergenic regions, which all had the same length (1kb), sequences with >500 gaps were removed.
2. We removed loci with fewer than 4 sequences as this is the minimum number of sequences needed to construct a tree.

## **Extraction of intergenic regions and filtering**

In order to investigate phylogenetic patterns across the genome, we designed a genome-wide sampling of putatively intergenic regions. These regions were evenly sampled in regular intervals, as follows. We first built a pipeline to convert the Cactus whole genome alignment from HAL to MAF format using chicken as the reference and to extract the best aligned synteny blocks from each query species using 10 kb windows (<https://github.com/Secretloong/Cactus_Alignments_Tools>). We selected consecutive regions of 10 kb length while skipping regions that were repetitive in chicken or those only present in Galliformes but not in other birds. Among the first 2 kb of each 10 kb window, the 1 kb portion with the most site-wise occupancy was selected to avoid regions with few sequences. Each locus used for gene tree estimation was therefore 8-9 kb away from the next locus, avoiding strong linkage between loci. These 1 kb loci were used in gene tree reconstruction.

The decision to use 1 kb windows from which to estimate gene trees was made after preliminary assessments ([Extended Data Fig. 1](https://docs.google.com/document/d/1bj4uF4M3_FdVb8tKTfPkT2Y5HV5k0cjKcQt4lRsfWv4/edit#equ_Method)d). When building gene trees from genomic regions, there are trade-offs between the length of the locus (and hence its informativeness) and the probability of spanning recombination breaks [^2^](https://paperpile.com/c/QHZ1Z3/csSu2): Too short of a locus contains too little information and gene tree estimation error will be high, too long of a locus and the chance of containing multiple evolutionary histories increases. We extracted 500 alignments of 10 kb and selected from these windows of 5, 2.5, 1.5, 1, 0.5, 0.25 kb length, each minimizing missing data. We then estimated gene trees using IQTREE [^3^](https://paperpile.com/c/QHZ1Z3/yIWkD) v.1.6.12 with 1000 ultrafast bootstraps [^4^](https://paperpile.com/c/QHZ1Z3/m3Mf3). We then assessed the number of nodes that had high bootstrap support of >75% as a proxy for the information content of the locus. We found that at 1kb the proportion of highly supported nodes started to taper and increases were less pronounced. We also compared how support in individual loci changed when their alignment length was increased. We found that going from 0.25 to 0.5 and from 0.5 to 1 kb, most gene trees improved in their number of highly supported nodes. At increasing lengths, we found that an increasing number of loci did not change the number of highly supported nodes or that the number of highly supported nodes even decreased. We therefore chose 1 kb as a good solution balancing sufficient information content of the gene tree, while minimizing locus length.

This resulted in 94,402 loci of genome-wide regions, for which we estimated gene trees. We created smaller subsets of this dataset in order to assess the impact of including loci that partly overlapped with exons or introns ([Extended Data Fig. 1](https://docs.google.com/document/d/1bj4uF4M3_FdVb8tKTfPkT2Y5HV5k0cjKcQt4lRsfWv4/edit#equ_Method)b). Based on the chicken genomic annotation and coordinates, we identified 1 kb loci which had any overlap with exons (14,355 loci) or introns (16,617 loci). Subtracting these from the total loci resulted in a dataset of 63,430 purely intergenic loci, which were used to construct the main tree.

## **Extraction of introns and filtering**

Extraction of introns followed previously described procedures [^1^](https://paperpile.com/c/QHZ1Z3/mJFTa). We constructed individual gene trees for each intron of the same gene. This resulted in a total of 44,846 loci of introns. The number of assessed introns and total alignment length ([Extended Data Fig. 1](https://docs.google.com/document/d/1bj4uF4M3_FdVb8tKTfPkT2Y5HV5k0cjKcQt4lRsfWv4/edit#equ_Method)) is a significant increase from the Jarvis et al. [^5^](https://paperpile.com/c/QHZ1Z3/itxOX) dataset (2516 intron loci, 19 Mb filtered and aligned).

## **Alignment and filtering of protein-coding regions**

Protein-coding regions were obtained from available genome annotations [^1^](https://paperpile.com/c/QHZ1Z3/mJFTa). Due to the difficulty in gene prediction, protein-coding regions extracted from genomes can include potentially non-orthologous segments [^6^](https://paperpile.com/c/QHZ1Z3/2iYzq). We devised a strategy to minimize these artifacts in our analyses, without aggressively trimming alignments as the loss in data can be worse than imperfect orthology. After removal of fragmentary sequences and exclusion of loci with fewer than 4 taxa as described above, we employed the following filtering steps:

1. After initial alignment, we aimed to remove sequences which appeared to be mostly unrelated to others. This was done using TreeShrink [^7^](https://paperpile.com/c/QHZ1Z3/4F4J7) v1.3.1, which removes sequences leading to uncharacteristically long branches for the species.
2. We observed that regions of questionable orthology in the protein-coding alignments often involved fragments of individual sequences that appeared unaligned with respect to other sequences. To identify these segments, we used MAFFT [^8^](https://paperpile.com/c/QHZ1Z3/ppElH) v7.149b with the G-INS-i algorithm with a variable scoring matrix (VSM), which expects that sequences are globally conserved but locally contaminated by unrelated segments such as non-orthologous regions [^9^](https://paperpile.com/c/QHZ1Z3/1lpmu). The approach isolates blocks of aligned sequences from unrelated segments, which can then be removed from the alignment.

Each locus was first aligned as amino acids to facilitate better alignment. The initial alignment was performed with the iterative aligner PASTA [^10^](https://paperpile.com/c/QHZ1Z3/PkwWy) v1.8.5 using two iterations of MAFFT G-INS-i alignment (command: mafft --globalpair --unalignlevel 0.8 --allowshift --maxiterate 1000). This was followed by filtering step 1 in a wrapper including TreeShrink as part of PASTA (<https://github.com/uym2/TreeShrink/tree/master/related_scripts>). A second round of alignment was performed using one iteration of MAFFT G-INS-i. Amino acid alignments were back-translated to result in nucleotide alignments. To perform filtering step 2, strongly gapped regions were removed as they can be indicative of being non-homologous. We therefore removed codons that were gaps in >99% of sequences. Lastly, we excluded the third codon position, as its inclusion was previously shown to result in trees with different topologies [^5^](https://paperpile.com/c/QHZ1Z3/itxOX). This resulted in a final set of 14,972 alignments of the first and second codon position.

The number of protein-coding regions represents a significant increase in protein-coding data compared to the Jarvis et al. [^5^](https://paperpile.com/c/QHZ1Z3/itxOX) dataset (8251 filtered ortholog loci).

## **Extraction, alignment, and filtering of Ultraconserved Elements (UCEs)**

UCE loci were extracted from genome assemblies using commands implemented in PHYLUCE [^11^](https://paperpile.com/c/QHZ1Z3/5nznw) v1.6.3 (commit 185b705) targeting 5060 UCE loci and 1000 bp flanking regions. Of 5031 UCEs, 5006 loci remained after the common minimal filtering. Alignment and exclusion of outliers was conducted following a three-step process similar to the processes described for protein-coding regions. Specifically, an initial alignment was performed using PASTA v1.8.5 with MAFFT v7.149b and the L-INS-i algorithm, followed by one round of TreeShrink v1.3.1 (-b 10, -s 2,5) for outlier removal, followed by a final alignment using PASTA with MAFFT L-INS-i. After the removal of outliers, a total of 4985 loci of UCEs remained.

## **Inference of gene trees and summary into species trees**

A total of 159,205 gene trees across all datatypes were estimated using Pargenes [^12^](https://paperpile.com/c/QHZ1Z3/nL24V) v.1.1.0, which employs substitution model selection using Modeltest-NG [^13^](https://paperpile.com/c/QHZ1Z3/6E13u) v0.1.3 and RAXML-NG [^14^](https://paperpile.com/c/QHZ1Z3/hnU8h) v.0.9.0 with 10 random starting trees and 10 parsimony starting trees and scaled branch lengths (command: pargenes-hpc.py -a $IN -o $OUT -d nt -c $CORES -s 10 -p 10 -m -R “--brlen scaled”). We did not obtain statistical support for individual gene trees through bootstrap resampling because of the computational cost on such a large number of loci. Instead, we were primarily interested in identifying poorly supported nodes, in order to collapse these before analyzing them in summary methods as this is known to reduce the impact of these low supported gene tree nodes on species tree reconstruction [^15^](https://paperpile.com/c/QHZ1Z3/wozwn). To identify poorly supported nodes, we used IQTREE [^3^](https://paperpile.com/c/QHZ1Z3/yIWkD) v.1.6.12 to perform parametric approximate likelihood ratio tests (aLRT), which are fast tests of the three possible nearest-neighbor resolutions around a branch [^16^](https://paperpile.com/c/QHZ1Z3/aD5yf). The best-fit substitution model chosen by Modeltest-NG and the best ML tree from RAxML-NG were used for computing aLRT scores (command: iqtree -s $ALIGNMENT -te $RAXML_TREE -m $MODELTEST_MODEL -alrt 0 -nt 1 -pre alrt_support/$OUT). Poorly supported inner branches were contracted to polytomies using newick-utilities [^17^](https://paperpile.com/c/QHZ1Z3/ESscM) v.1.6 if the respective aLRT value was <0.95 (command: cat $ALRT_TREES | sed "s/\///g" | nw_ed - 'i & (b<0.95)' o > $COLLAPSED_TREES). Collapsed gene trees were summarized into a coalescent-based species tree using ASTRAL-III [^15^](https://paperpile.com/c/QHZ1Z3/wozwn) v.5.14.5.

ASTRAL species trees are already furnished with estimated coalescent unit lengths and a local posterior probability (PP) on internal branches [^18^](https://paperpile.com/c/QHZ1Z3/3dpNt). We used the --gene-only option of ASTRAL to also perform gene-only multi-locus bootstrapping to obtain global support (globalBS), which is known to be more conservative than the generally more accurate local PP [^18^](https://paperpile.com/c/QHZ1Z3/3dpNt). We report the bootstrapping results in cases where uncertainty is not local (e.g., two placements many branches away both result in high quartet support), a scenario that can mislead the local PP support. Additionally, we used the -t 10 option to test a polytomy null hypothesis [^19^](https://paperpile.com/c/QHZ1Z3/QmVia), and the -t 2 option to evaluate the quartet score of the three alternative nearest neighbor interchanges (NNI) around each branch. These quartet scores were visualized using DiscoVista [^20^](https://paperpile.com/c/QHZ1Z3/QOWlO). We evaluated alternative species trees (e.g., moving Phaetontimorphae) by scoring these trees against the same input gene trees using the -q option of ASTRAL.

## **Concatenation-based species tree**

We used RAXML-NG [^14^](https://paperpile.com/c/QHZ1Z3/hnU8h) v.1.0.1 to perform maximum likelihood (ML) tree inference from a concatenated alignment of the 63k intergenic loci. We used one partition per locus (63k partitions) with respective evolutionary models determined by Modeltest-NG as described above. We ran 20 independent searches from 20 random starting trees and picked the highest-scoring tree as our best-known ML tree. We then ran 50 tree searches on bootstrap (BS) pseudo-replicate alignments, and drew bootstrap support values on the best-known ML tree. We applied the MRE bootstrap convergence criterion [^21^](https://paperpile.com/c/QHZ1Z3/9tS3W) to confirm that 50 replicates were sufficient to obtain stable support values. To save time and energy, we used a topological constraint for all tree searches (ML and BS). The constraint tree was a strict consensus of the trees from the 63k intergenic regions, exons, introns, and UCEs obtained with ASTRAL and of an initial run with RAxML-NG on the 63k intergenic loci (based on 10 tree searches with 5 random+5 parsimony, no bootstraps). This consensus left the backbone nodes free to be inferred while constraining uncontroversial nodes within orders (317 nodes resolved, 45 nodes collapsed).
Commands:
ML tree search (20): raxml-ng-mpi --search --msa $ALIGNMENT --tree rand{1} --tree-constraint $CONS_TREE --prefix $NAME --seed $SEED --threads $CORES
Bootstrap search (50): raxml-ng-mpi --bootstrap --msa $ALIGNMENT --tree rand{1} --bs-trees 1 --tree-constraint $CONS_TREE --prefix $NAME --seed $SEED --threads $CORES

BS convergence test: raxml-ng-mpi --bsconverge --bs-trees bs50.nw --prefix bsconverge

Plot support: raxml-ng-mpi --support --tree $BEST_TREE --bs-trees $BS50_TREES --prefix $NAME --bs-metric fbp

## **Fossil calibrations and derivation of calibration densities**

For node-based fossil calibrations, we derived calibration distributions empirically using the recently described CladeDate method [^22,23^](https://paperpile.com/c/QHZ1Z3/jBoVw+P3XpQ). We first selected crown clades that were well supported across phylogenomic analyses and that had a good-quality fossil record. Within each clade, we identified the oldest fossil that fulfilled best practices criteria for fossil calibrations [^24^](https://paperpile.com/c/QHZ1Z3/SXwH6). In particular, we identified fossils that were composed of multiple bones, were chronologically well constrained, and whose phylogenetic position within the crown-clade was supported by explicit phylogenetic analyses or strong apomorphy-based evidence.

To generate calibration densities, CladeDate requires a sample of the fossil record of each calibration clade. For widespread clades, we generated this sample by identifying the oldest fossil in each continental landmass. Restricting the sample to one fossil per landmass minimizes problems of spatial non-independence and confounding histories of dispersal [^22^](https://paperpile.com/c/QHZ1Z3/jBoVw). For clades restricted to single landmasses, we used the oldest fossils of different species in different geological formations.

With a set of fossil ages for each clade, we then used CladeDate to generate distributions representing the uncertainty of the age of the calibration clades. CladeDate uses various estimators of the location of the upper bound (age) of truncated distributions to generate estimates of the age of clades based on the sample of fossils. When the distribution of fossils ages did not depart significantly from a uniform distribution, we used the Strauss and Sadler [^25^](https://paperpile.com/c/QHZ1Z3/BigLV) estimator, otherwise, we excluded the Quaternary record to improve uniformity or used various estimators that do not assume sample uniformity [^23^](https://paperpile.com/c/QHZ1Z3/P3XpQ). In addition to point estimates, CladeDate uses a Monte Carlo procedure for generating a distribution of ages representing estimation uncertainty. In addition, CladeDate accounts for fossil age uncertainty by resampling fossil ages from their chronostratigraphic intervals as part of a Monte Carlo procedure. The resultant Monte Carlo distribution of clade ages was used to estimate the parameters of a Student-skew distribution that was then used to parameterize calibration priors in MCMCtree (see below). For further details about the CladeDate method see [^23^](https://paperpile.com/c/QHZ1Z3/P3XpQ). Details of the calibration clades, fossils used, and empirical density estimation are provided in the section ‘Fossil Calibrations’.

## **Molecular dating of the species tree**

To infer a time-calibrated tree, we performed molecular dating using a subset of the genomic data. First, we estimated phylograms using maximum likelihood in IQTREE [^26^](https://paperpile.com/c/QHZ1Z3/d02Dh) v2.0.4 for all 63k alignments, with the tree topology fixed to that of the main tree. We used the GTR+F+R4 substitution model, which was the modal best-fit model of the family across a random sample of 100 loci. Phylograms were then rooted using minimum variance rooting in the FastRoot program [^27^](https://paperpile.com/c/QHZ1Z3/CKoIP). We selected the loci with intermediate evolutionary rates by removing the 25% of loci with the smallest mean root-to-tip distances and the 25% with the largest mean root-to-tip distances. Of the remaining loci, we selected the 40% with the lowest coefficient of variation in root-to-tip distances, thereby retaining the most clocklike loci for phylogenomic dating.

We randomly divided the remaining 10,494 loci into two groups of 5,247. Within each group we partitioned loci into subsets based on the size of the chromosome bearing the locus: macrochromosomes, intermediate chromosomes, and microchromosomes. The two locus groups were used for dating in a Bayesian sequential subtree approach [^28^](https://paperpile.com/c/QHZ1Z3/OkabW), where the 363-taxon tree was divided into a backbone tree (56 tips) containing two representatives of each of 11 subtrees (19–42 tips each). The posterior distributions of the ages of the 11 nodes in the backbone tree that corresponded to the root nodes of the subtrees were fitted with *skew-t* densities using the ﻿R function sn::st.mple v.2.0.0, under the BFGS method for parameter optimisation [^29^](https://paperpile.com/c/QHZ1Z3/5JQEV). The *skew-t* parameters were then used to specify the prior distributions of root ages for the dating analyses of the subtrees. After dating the backbone tree and the 11 subtrees, we attached the subtrees to the backbone tree to assemble a complete timetree of all 363 taxa. Half of the loci were used to date the backbone tree and the other half were used to date the subtrees, thus avoiding data duplication in the likelihood.

Bayesian molecular dating was conducted using MCMCtree v.4.9h from the PAML package [^30^](https://paperpile.com/c/QHZ1Z3/UpIkN), with approximate likelihood calculation [^31^](https://paperpile.com/c/QHZ1Z3/ljAGL). The analyses included all calibration priors plus a minimum bound on the age of the root based on the uncontroversial neornithine fossil of *Waimanu manneringi* [*^32^*](https://paperpile.com/c/QHZ1Z3/AU6CK). Our main analysis also included a soft maximum bound on the root at 86.5 Ma (but see [Extended Data Fig. 5](https://docs.google.com/document/d/1bj4uF4M3_FdVb8tKTfPkT2Y5HV5k0cjKcQt4lRsfWv4/edit#equ_age_estimates)c for results with a Jurassic age bound). Nucleotide substitutions were modeled using the GTR+G model. Nodes without calibrations followed a birth-death process prior [^33^](https://paperpile.com/c/QHZ1Z3/msMrk) with parameters *λ* = *μ* = 1 and sampling fraction *ρ* = 0.1, which gives an approximately uniform kernel. Divergence times were estimated using a relaxed clock with lognormally distributed rates across branches and a gamma-Dirichlet prior on rates across the three subsets of loci [^34^](https://paperpile.com/c/QHZ1Z3/LLzP0).

Markov chain Monte Carlo sampling was used to estimate the posterior distribution of divergence times. Samples were drawn every 2500 steps over a total of 5.5×10^7^ steps after 5×10^6^ steps were discarded as burn-in. The analysis was run in duplicate, to check that independent runs converged to the stationary distribution and to ensure sufficient sampling. Subtrees were then attached to the backbone tree to produce a complete dated tree of all 363 sampled bird taxa.

In addition to the main dating analysis, we performed four dating analyses with alternative settings:

1. Uniform calibration priors. The use of *Skew-t* densities for calibration priors can lead to substantial violations of soft minimum bounds. To remove this possibility, we repeated analyses with uniform distributions with ranges spanning the 95% probability density of the original calibration prior, adding a soft maximum bound with a 2.5% tail of probability.
2. Jurassic age bound. An analysis included a relaxed maximum age bound of 201.3 Ma on the age of the root. This age corresponds to the boundary between the Triassic and Jurassic.
3. Calibration subset. An analysis focused on a subset of 23 calibrations that were considered to be the most reliable.
4. Randomly selected loci. Since data-filtering schemes carry the risk of producing biased subsets of loci, we performed a dating analysis with a set of 10,494 randomly selected loci from the 64k set. These loci were randomly split into two equal groups of 5,247 loci. Within each group, loci were randomly partitioned into three equal subsets of 1,749 loci for analysis using a Bayesian sequential subtree approach.

## **Subsetting analyses**

### **By taxon sampling**

In order to investigate the impact of sampling multiple species for most of the avian orders (which represent the deepest and most contentious branches), we successively reduced the taxon sampling within orders from the available number down to 50, 25, 10, 9, 8, 7, 6, 5, 4, 3, 2, or 1 species per order. We did so by randomly selecting species from the existing gene trees, retaining all species if less than the desired number were available. We then scored the main tree against each of these taxon-reduced gene trees, using ASTRAL annotation tools (-t 2 option) to compute the normalized quartet support for the three alternative topologies around each of the species tree branches. These values add up to one, and the topology with the highest support is one that is most supported by those gene trees. Under an ILS-only model, the second and third topologies are expected to have roughly equal quartet supports, which should be lower than ⅓. Deviations from this pattern are indicative of non-ILS discordance due to methodological factors such as long branch attraction and model violation or biological factors such as hybridization.

These analyses showed substantial impact only for Accipitriformes and that more than 50 species were required to recover the main clade. Since the only order with more than 50 taxa was Passeriformes, we inferred that their sampling impacted the position of Accipitriformes and Strigiformes. In order to test this hypothesis, we randomly shuffled the Passeriformes in two replicates. We then removed the first 1, 3, 5, 7, ..., 171 of the 173 Passeriformes and for each replicate, and used ASTRAL to compute the quartet scores with gene trees restricted to that subset. We drew the support for each topology for both replicates but note that the two replicates produced indistinguishable results.

### **By data quantity**

Of the total 63k gene trees that constituted the main tree, we randomly selected subsets of increasing numbers of gene trees with a maximum sample of half of the available gene trees (1000, 2000, 4000, 8000, 16000, 32000). Each subset was repeated 50 times and for each subset of gene trees, we estimated an ASTRAL species tree. To gauge topological similarity, the subset tree topology was compared to the main topology by counting the number of differing branches (i.e. Robinson-Foulds (RF) distance/2). We also calculated the proportion of highly supported branches (PP≥0.95).

In order to estimate the impact of data quantity on the stability of certain nodes, we traversed over each clade of the main tree and recorded whether the clade was present across the resulting species trees. We recorded how many loci were needed to recover each clade across all 50 replicates of a subset of n loci. For each clade in the main tree, we also counted how many different sister groups were present across the 50 replicates of each subset.

We also performed the same analyses for the other data types, maximally sampling about half of the total available loci. This included exons (repeatedly sampling subsets of 1000, 2000, 4000, 8000 gene trees), introns (1000, 2000, 4000, 8000, 16000, 32000), and UCEs (1000, 2000). We also performed the analyses using all non-coding (80k windows, intron, UCEs, total 129,878 loci) gene trees together (1000, 2000, 4000, 8000, 16000, 32000, 64000).

### **By data type**

In addition to investigating the effect of increasing data quantity for each data type, we also compared the magnitude of differentiation between the resulting trees for each data type. For this analysis, we controlled for the number of gene trees used. As before, we subsampled loci at random (50 replicates). The highest number of gene tree subsets present across all data types was 2000 (because the number of loci was limited by the total number of UCEs). To show the impact of increasing loci, we also performed the analysis for 8000 loci, omitting comparisons with UCEs. For all resulting species trees, we calculated mean pairwise RF distances computed using TreeCmp v.2.0 [^35^](https://paperpile.com/c/QHZ1Z3/xM0Qq).

### **By genomic characteristics**

We calculated a range of metrics for all gene trees and the underlying alignments for each locus of all data types. Metrics pertaining to the gene trees included number of taxa, tree length (the sum of all branch lengths, an indicator overall substitution rate of the locus), tree diameter (the average root-to-tip distance for each terminal using newick-utilities’ nw_distance), stemminess (ratio of the lengths of internal branches to terminal branches), clocklikeness (the coefficient of variation in root-to-tip distance), mean branch support (average aLRT support value across the gene tree), and proportion of branches with aLRT support above 95 and 99. Metrics pertaining to the underlying alignment were locus length, total coverage (the number of gap characters in the alignment compared to nucleotide characters), the number of parsimony informative sites, the proportion of parsimony sites, and the mean and standard deviation of GC content (calculated with seqkit v.2.2.0 [^36^](https://paperpile.com/c/QHZ1Z3/wXPeC)). For each alignment, we additionally predicted the difficulty of phylogenetic estimation under maximum likelihood, i.e. whether the alignment is likely to result in multiple, topologically highly distinct yet statistically indistinguishable topologies, using Pythia [^37^](https://paperpile.com/c/QHZ1Z3/XrScC) v. 1.0.0. In order to estimate the impact of the magnitude of these metrics on phylogenetic reconstruction, we divided loci into four equal-sized quantiles based on their values for each metric. For this, we focused on the intergenic loci without overlap from exons (80,045 loci). Consequently, each quantile consisted of 20,011 loci. We then estimated a species tree with ASTRAL for each quantile and compared them to the main species tree.

### **By chromosome and chromosomal category**

We built separate species trees from gene trees of each chromosome according to their chromosomal assignment in chicken. We excluded very small chromosomes with <1000 gene trees (chr15, chr16, upwards from chr21), resulting in 16 chromosome species trees (5 macro-, 5 intermediate, 5 microchromosome, Z chromosome). We also built a species tree for each of the major chromosome size categories recognized in birds [^38^](https://paperpile.com/c/QHZ1Z3/L2CCT): The species tree for macrochromosomes (50-200 Mb, chr1-5) was built from 50k gene trees; for intermediate chromosomes (20-40 Mb, chr6-10) from 12k gene trees; and for microchromosomes (average size 12 Mb, chr11-38) from 13k gene trees; the Z sex chromosome contained 5672 gene trees.

In order to investigate patterns across the chromosome, we used the 94k regions. For each gene tree (with low support nodes collapsed aLRT<0.95), we calculated RF distances against the main tree with TreeCmp, which we normalized to account for different numbers of nodes in each gene tree. We investigated the potential genomic co-localization of gene tree discordance with two factors. First, we plotted the standard deviation of GC content for each alignment, because high deviations from the GC content of other species violate common model assumptions. Secondly, we incorporated recombination rates estimated for chicken (males so that recombination rates including the Z chromosome could be used), originally estimated for bins of ca. 500 kb [^39^](https://paperpile.com/c/QHZ1Z3/suMAZ). We used the same bins as used in that study to estimate mean normalized RF distances and GC standard deviation for each bin.

## **​​Phylogenetic model adequacy**

We tested for misleading inferences due to phylogenetic model violation. Specifically, we tested for excessive amounts of non-stationary base-composition using Foster’s posterior predictive simulations method [^40^](https://paperpile.com/c/QHZ1Z3/xQQ6C), adapted to maximum likelihood using a parametric bootstrap [^41^](https://paperpile.com/c/QHZ1Z3/WyuMd). We also tested the data for misleading inferences due to substitution saturation using entropy tests on parsimony-informative sites [^42^](https://paperpile.com/c/QHZ1Z3/GMWlq). For both tests, we implemented thresholds of assessment based on simulations, as described in the original studies. These thresholds define loci as having high risk of misleading inferences under simulation scenarios where all simulations yielded inaccurate inferences.

## **CoalHMM analyses**

Instead of reconstructing trees by averaging over a genomic locus, phylogenies can also be inferred on a single-base-pair level. These approaches are computationally demanding and currently limited to four taxa, which need to be tested in all possible configurations. CoalHMM was used to calculate the proportion of ILS of two clades that were difficult to resolve in our main analyses, namely Rheiformes and Strigiformes+Accipitriformes. In brief, CoalHMM is a hidden Markov model that runs along the genome and fits speciation times and ancestral effective population sizes given the multiple alignment of three species, an outgroup, and the underlying species tree. After model fitting, CoalHMM performs posterior decoding on the hidden states in order to assign a probability of each site belonging to each of four different topologies. Thus, it allows estimating the proportion of ILS, which is defined as the proportion of sites that are assigned to either of the two deep coalescence topologies that do not match the species tree. CoalHMM was run using an automated pipeline [^43^](https://paperpile.com/c/QHZ1Z3/1yPpC), where the alignment blocks were filtered and split into 1Mb chunks on which CoalHMM was run independently.

First, we tested all possible combinations of the placement of Rheiformes within the Palaeognathae tree using one representative (selected to be the most contiguous genome) of kiwis (*Apteryx owenii*), cassowaries (*Casuarius casuarius*), tinamous (*Nothoprocta perdicaria*) and rheas (*Rhea americana*). Assuming that kiwi and cassowary are always sister species as indicated by our analyses, then rhea has only three possible placements: ((kiwis, cassowaries), (tinamous, rheas)); or ((kiwis, cassowaries), rheas), tinamous)); and ((kiwis, cassowaries), tinamous), rheas));. Using only three of these species and the chicken as an outgroup, there are 8 possible choices for each of the three possible species trees. CoalHMM was run for each of these combinations and for all chromosomes. Afterwards, the best fitting topology was chosen based on the posterior probabilities. More specifically, under an ILS model and in the absence of other phenomena such as ancient introgression or hybridization, the proportion of the deep coalescence topologies should be equal, and the ILS proportion should not exceed the maximum (2/3).

Using a similar rationale, ILS proportions were calculated for the Strigiformes/Accipitriformes clade. Using Passeriformes (*Sporophila hypoxantha*) as the outgroup, we analyzed all possible combinations of species trees for owls (*Glaucidium brasilianum*), hornbills (*Bucorvus abyssinicus)* and eagles (*Aquila chrysaetos*). There are three possible species trees, namely (Passeriformes, (owls, (hornbills, eagles))); or (Passeriformes, (hornbills, (owls, eagles))); and (Passeriformes, (eagles, (hornbills, owls)));. The best-fitting species tree was chosen as described for rheas.

## **GC content differences within Palaeognathae**

To interrogate the impact of convergent GC content on gene trees, we defined a measure of GC similarity. For any three groups of taxa, X, Y, and Z, we could use

$\frac{1}{(|X|+|Y|)(|Z|)}\sum_{a\in X\cup Y} \sum_{z\in Z} (a-z)^{2}-\frac{1}{|X||Y|}\sum_{x\in X} \sum_{y\in Y} (x-y)^{2}$

to measure the similarity of X and Y beyond their similarity to Z in terms of any measure, including the GC content. To use this approach, we computed GC content per species across the 63k intergenic loci, of which 54651 loci had all relevant species present. We averaged the values for each of the four clades: Tinamiformes ($T$), Rheiformes ($R$), Apterygiformes ($A$), and Casuariiformes ($C$). Then, we defined a quantity measuring how similar T and R are compared to how similar either T or R are to either A or C:

$\Delta GC = \left( (T-C)^{2}+(T-A)^{2}+ (R-C)^{2}+(R-A)^{2} \right)/4 - (T-R)^{2}$

This quantity ($\Delta GC$) should be zero under the stationary models of evolution used for gene tree inference. It will have high positive values when *T* and *R* are similar to each other but different from *C* and *A*. It will have low negative values when *T* and *R* are dissimilar compared to their similarity to *A* and *C*. Either positive or negative values inculcate deviation from the model, with positive values pulling *T* and *R* towards each other and negative values having the reverse effect.

We created subset of loci by removing those that had $\Delta GC$values that diverged substantially from 0. We created nine subsets of the loci: those with $\Delta GC < 0.0001$ (51731 loci), $\Delta GC < 0.001$ (62321 loci), $\Delta GC < 0.0025$ (63108 loci), $\Delta GC > -0.0001$ (38956 loci), $\Delta GC > -0.001$ (54969 loci), $\Delta GC > -0.0025$ (59867 loci), $-0.0001<\Delta GC < 0.0001$ (27257 loci), $-0.001<\Delta GC < 0.001$ (53860 loci), and $-0.0025<\Delta GC < 0.0025$ (59545 loci). We ran ASTRAL on each subset, and all of them united *R* and *T*. We computed a normalized quartet score around the branch uniting *R* and *T* for each subset to investigate whether subsets with high $\Delta GC$ removed had lower quartet support for uniting *R* and *T*.

## **Inference of effective population size**

We compared the time-calibrated tree with the ASTRAL tree where all internal branches have a calculated estimated coalescent unit length [^18^](https://paperpile.com/c/QHZ1Z3/3dpNt). For each internal branch, we computed the ratio of the branch length in time units to the branch length in coalescent units. If we ignore errors in these estimates, by coalescent theory, this ratio is equal to:

$\frac{time unit}{coalescent unit}=\frac{generation time \times number of generations}{number of generations/{2N}_{e}}=\frac{1}{2}generation time\times N_{e}$.

Higher values are indicative of higher population size (*N*_e_) or change in generation time. Ignoring changes to generation time, higher time/coalescent ratios can be attributed to higher population sizes. Around the K-Pg-boundary, the generation times are presumed to have decreased, which makes the increases in our measured quantity indicative of even larger population size growth than what would be inferred if generation times are assumed constant. Note that summary methods such as ASTRAL are known to under-estimate coalescent unit length in the presence of high gene tree estimation error. However, we only compare branches to each other, without claiming to estimate the true *N*_e_. Thus, as long as gene tree estimation error is not particularly concentrated on specific nodes, it should not impact the relative values.

## **Analysis of molecular evolutionary rates**

Genome-wide evolutionary rates were estimated for each branch of the species tree using the 63k set of intergenic loci. To minimize the estimation bias in substitution rates arising from discordance between the species tree and gene trees [^44^](https://paperpile.com/c/QHZ1Z3/zLQ1U), we only considered branches across gene trees that were concordant with the species tree [^45^](https://paperpile.com/c/QHZ1Z3/moKex). Each concordant branch length from gene trees was divided by the time duration of the branch from the main timetree analysis, leading to a rate estimate for each species-tree branch for each locus. For comparison, we estimated branch lengths of the gene trees while fixing the topology to that of the main species tree, which would maximize the number of rate estimates but reduce the accuracy of estimates of individual branch rates. This approach led to broadly similar estimates of mean rates across branches.

## **Analysis of phylogenetic signal**

Phylogenetic signal was measured for morphological traits using Pagel’s lambda [^46^](https://paperpile.com/c/QHZ1Z3/PFisH) based on the main topology of this study and the Prum et al. [^47^](https://paperpile.com/c/QHZ1Z3/i3xhR) topology, respectively. Given the different species numbers contained in the two topologies, we also randomly sampled species from the main topology to match the sample size of Prum (N=198). We did not include the Jarvis et al. [^5^](https://paperpile.com/c/QHZ1Z3/itxOX) topology because it was too sparsely sampled for this analysis, nor the Kuhl et al. [^48^](https://paperpile.com/c/QHZ1Z3/ofM7l) topology because the time-calibrated tree was not publicly available. We sampled traits from the near-complete AVONET [^49^](https://paperpile.com/c/QHZ1Z3/oCCRP) dataset: body mass, beak length, depth, and width, tarsus length, wing length, Kipp’s distance, and tail length. As Apterygiformes do not have wings or tails, these species were excluded when analyzing wing length, Kipp’s distance, and tail length. We calculated Pagel’s lambda for each trait using 100 simulations (boot=100) of continuous characters on the phylogeny using the R package phylolm [^50^](https://paperpile.com/c/QHZ1Z3/q80ox). One-sided t-tests were performed to test for statistical significance of differences between the main topology or the subsampled main topology against the Prum topology.

In order to further account for the differences in taxon sampling between our study as that of Prum et al. study, we performed two tests. First, we pruned both trees to the 124 taxonomic families that overlapped between the two studies. This assured that both trees have the same number of taxa, representing the same lineages. Second, we pruned all but one species of Passeriformes (*Menura novaehollandiae*) from both tree topologies. This test aimed at testing whether the large number of passeriform taxa in our study impacted the results. Pagel’s lambda was estimated as described above. Results are given in the Supplementary Results below.

To investigate the effect of an incorrect species tree topology on Pagel’s lambda, we simulated a list of continuous traits on the main tree (N=363) under a Brownian motion model using the R package fastBM [^51^](https://paperpile.com/c/QHZ1Z3/7v8KQ). The true value of Pagel’s lambda on the simulated data was 0.96. We then randomly changed the position of a 1%, 5%, 10%, and 20% of the species on the phylogeny to represent incorrect species relationships. We repeated each analysis 100 times. Pagel’s lambda values were estimated under these incorrect topologies and compared to the true value of the simulation. To investigate the effect of convergent evolution on the estimation of Pagel’s lambda, we introduced convergence in trait values in distantly related species. We randomly selected species pairs, each consisting of one passeriform and one non-passeriform, representing 1%, 5%, 10% and 20% of the 363 species. Each species pair was then given the same trait value to simulate the action of convergent evolution. This way, an increasing number of Passeriformes and non-Passeriformes had identical trait values due to convergence. After repeating the analysis 100 times, we estimated Pagel’s lambda and compared them to the true value of the simulation.

## **Analysis of body mass and brain size evolution**

We obtained body mass data for 363 species [^49,52^](https://paperpile.com/c/QHZ1Z3/oCCRP+udxrK) and estimated brain size (volume of the brain case) for 228 species based on endocast volume, or back-calculated from brain mass using the formula: brain volume = brain mass/1.036 [^53^](https://paperpile.com/c/QHZ1Z3/IoEg1). For body mass and brain size, we used the average of males and females or mean unsexed values when available. For the brain size, we then used the missForest R package [^54^](https://paperpile.com/c/QHZ1Z3/TMdYO) to impute missing values based on phylogenetic relatedness among species, and finally obtained a dataset of brain size containing 363 species with an Out of Box error (OOBerror) of 0.0003. The relative brain size was calculated as the residual from a log-log phylogenetic Generalized Least Square regression of absolute brain size against body mass. Ancestral states of body mass (log-transformed) and the relative brain size were reconstructed by Evomap using a multiple variance Brownian motion approach [^55^](https://paperpile.com/c/QHZ1Z3/ATlXJ). The variations in body mass and the relative brain size were summarized by dividing the phylogeny into one-million-year time bins, then computing the mean values of all branches present in each bin.

The rates of evolution in body mass (log-transformed) and relative brain size were analyzed using BayesTraits v4 [^56^](https://paperpile.com/c/QHZ1Z3/D3ZGp) with variable rates models and default priors. The variable rates model allows the rate of change to vary through time and identifies areas of the tree where the rate of evolution differs significantly based on the posterior probability. Each analysis ran for 110,000,000 iterations with a burn-in of 10,000,000, and was carried out three times to confirm that the Markov Chain reached convergence. We used the Gelman and Rubin’s convergence diagnostic test statistics implemented as the gelman.diag function in the R package coda [^57^](https://paperpile.com/c/QHZ1Z3/KtezS). The run with the highest mean marginal likelihood was selected. In addition to BayesTraits, we compared the fit of three single-process models (Brownian motion (BM), early burst (EB) and Ornstein–Uhlenbeck (OU)) using the fitContinuous function in the R package Geiger v2.0 [^58^](https://paperpile.com/c/QHZ1Z3/Qau5T). As the BayesTraits and the single-process models are not fitted in a common framework with consistent likelihood calculations, we used the mean of the rate-scaled trees output by BayesTraits and calculated the likelihood of a BM model fit to this tree with the same trait data [^59^](https://paperpile.com/c/QHZ1Z3/hYkUY). If there is evidence for non-BM trait evolution (e.g. rate heterogeneity) within the tree, a BM model applied to the rate-scaled tree should return a higher likelihood than a BM model applied to the timetree [^59^](https://paperpile.com/c/QHZ1Z3/hYkUY). Model fit was assessed using AIC ([Extended Data Fig. 10](https://docs.google.com/document/d/1bj4uF4M3_FdVb8tKTfPkT2Y5HV5k0cjKcQt4lRsfWv4/edit#equ_traits)ef).

To investigate whether our sampling of one species per family could impact ancestral reconstructions, we performed 100 ancestral reconstructions each with tip values modified to reflect the family’s range in body size. To achieve this, we simplified our timetree to one species per family and drew a body size value for each tip from the range within its family (calculated using the entire AVONET database). This way, the sample size remained the same in each run, while the trait values reflected the variations of the trait within each family. ([Extended Data Fig. 10](https://docs.google.com/document/d/1bj4uF4M3_FdVb8tKTfPkT2Y5HV5k0cjKcQt4lRsfWv4/edit#equ_traits)g). We also confirmed that inclusion of the imputed brain size values did not change the shape of the ancestral reconstruction compared to a dataset containing only values from the literature ([Extended Data Fig. 10](https://docs.google.com/document/d/1bj4uF4M3_FdVb8tKTfPkT2Y5HV5k0cjKcQt4lRsfWv4/edit#equ_traits)h).

# **Supplementary Results**

## **Relationships within Passeriformes**

We report several arrangements in Passeri that differed from studies based on UCEs or 5′-UTR sequences [^48,60–62^](https://paperpile.com/c/QHZ1Z3/5Hs5O+ofM7l+8BGye+grD26), including the positions for Orioloidea, Malaconotoidea, Corvoidea, Mohouidae, and Neosittidae, Regulidae, Urocynchramidae, Irenidae and *Chloropsis* (highlighted with asterisks in [Fig. 3](https://docs.google.com/document/d/1bj4uF4M3_FdVb8tKTfPkT2Y5HV5k0cjKcQt4lRsfWv4/edit#fig_dataQuantity)d). We recovered Orioloidea as sister to Neosittidae, which together were sister to Malaconotoidea. Mohouidae was the sister group to Malaconotoidea+Neosittidae+Orioloidea, while Corvoidea was sister to the clade comprising all the aforementioned Corvides lineages. This differed from previous consistent placement of Orioloidea as sister to Malaconotoidea+Corvoidea [^48,60–62^](https://paperpile.com/c/QHZ1Z3/5Hs5O+ofM7l+8BGye+grD26) and suggestions of Mohouidae as sister to Neosittidae [^60,62^](https://paperpile.com/c/QHZ1Z3/5Hs5O+grD26) or to all of Corvides [^61^](https://paperpile.com/c/QHZ1Z3/8BGye). The difficulty in resolving the position of Mohouidae can be attributed to an extremely short branch (0.18 Ma). Within Muscicapida, we found Regulidae as sister to Muscicapoidea+Certhioidea, while previous studies placed Regulidae as sister to Certhioidea [^60,62^](https://paperpile.com/c/QHZ1Z3/5Hs5O+grD26) or as sister to Bombycilloidea [^48^](https://paperpile.com/c/QHZ1Z3/ofM7l). Within Passerida, the enigmatic monotypic Urocynchramidae was inferred as sister to Ploceidae+Viduidae+Estrildidae, in contrast to previous analyses [^60^](https://paperpile.com/c/QHZ1Z3/5Hs5O). Irenidae and *Chloropsis* have been reported as sisters before [^48,60,62^](https://paperpile.com/c/QHZ1Z3/5Hs5O+ofM7l+grD26) but were successive sister groups in our tree.

## **Impact of taxon sampling on phylogenetic signal**

When both the main tree and the tree from the Prum et al. study were pruned to have the same families represented (n=124), Pagel’s lambda values were significantly higher for the main topology for 7 out of 9 morphological characters. Kipp’s Distance and Tarsus Length showed no significant difference in inferred Pagel’s lambda values between the two trees (average λ main tree = 0.88, average λ Prum et al. = 0.78, *p* value = 0.0014691). When trees were pruned to remove all but one species of Passeriformes (main tree n=191, Prum et al. n=155), all 9 morphologically characters fit significantly better to the main tree than to the Prum et al. topology (average λ main tree = 0.98, average λ Prum et al. = 0.86, *p* value = 0.0000868).

# References

1. [Feng, S. *et al.* Dense sampling of bird diversity increases power of comparative genomics. *Nature* **587**, 252–257 (2020).](http://paperpile.com/b/QHZ1Z3/mJFTa)

2. [Springer, M. S. & Gatesy, J. Delimiting coalescence genes (C-genes) in phylogenomic data sets. *Genes* **9**, (2018).](http://paperpile.com/b/QHZ1Z3/csSu2)

3. [Nguyen, L.-T., Schmidt, H. A., von Haeseler, A. & Minh, B. Q. IQ-TREE: a fast and effective stochastic algorithm for estimating maximum-likelihood phylogenies. *Mol. Biol. Evol.* **32**, 268–274 (2015).](http://paperpile.com/b/QHZ1Z3/yIWkD)

4. [Hoang, D. T., Chernomor, O., von Haeseler, A., Minh, B. Q. & Vinh, L. S. UFBoot2: improving the ultrafast bootstrap approximation. *Mol. Biol. Evol.* **35**, 518–522 (2018).](http://paperpile.com/b/QHZ1Z3/m3Mf3)

5. [Jarvis, E. D. *et al.* Whole-genome analyses resolve early branches in the tree of life of modern birds. *Science* **346**, 1320–1331 (2014).](http://paperpile.com/b/QHZ1Z3/itxOX)

6. [Springer, M. S. & Gatesy, J. On the importance of homology in the age of phylogenomics. *System. Biodivers.* **16**, 210–228 (2018).](http://paperpile.com/b/QHZ1Z3/2iYzq)

7. [Mai, U. & Mirarab, S. TreeShrink: fast and accurate detection of outlier long branches in collections of phylogenetic trees. *BMC Genomics* **19**, 272 (2018).](http://paperpile.com/b/QHZ1Z3/4F4J7)

8. [Katoh, K., Misawa, K., Kuma, K.-I. & Miyata, T. MAFFT: a novel method for rapid multiple sequence alignment based on fast Fourier transform. *Nucleic Acids Res.* **30**, 3059–3066 (2002).](http://paperpile.com/b/QHZ1Z3/ppElH)

9. [Katoh, K. & Standley, D. M. A simple method to control over-alignment in the MAFFT multiple sequence alignment program. *Bioinformatics* **32**, 1933–1942 (2016).](http://paperpile.com/b/QHZ1Z3/1lpmu)

10. [Mirarab, S. *et al.* PASTA: ultra-large multiple sequence alignment for nucleotide and amino-acid sequences. *J. Comput. Biol.* **22**, 377–386 (2015).](http://paperpile.com/b/QHZ1Z3/PkwWy)

11. [Faircloth, B. C. PHYLUCE is a software package for the analysis of conserved genomic loci. *Bioinformatics* **32**, 786–788 (2016).](http://paperpile.com/b/QHZ1Z3/5nznw)

12. [Morel, B., Kozlov, A. M. & Stamatakis, A. ParGenes: a tool for massively parallel model selection and phylogenetic tree inference on thousands of genes. *Bioinformatics* (2018) doi:](http://paperpile.com/b/QHZ1Z3/nL24V)[10.1093/bioinformatics/bty839](http://dx.doi.org/10.1093/bioinformatics/bty839)[.](http://paperpile.com/b/QHZ1Z3/nL24V)

13. [Darriba, D. *et al.* ModelTest-NG: a new and scalable tool for the selection of DNA and protein evolutionary models. *Mol. Biol. Evol.* **37**, 291–294 (2020).](http://paperpile.com/b/QHZ1Z3/6E13u)

14. [Kozlov, A. M., Darriba, D., Flouri, T., Morel, B. & Stamatakis, A. RAxML-NG: a fast, scalable and user-friendly tool for maximum likelihood phylogenetic inference. *Bioinformatics* **35**, 4453–4455 (2019).](http://paperpile.com/b/QHZ1Z3/hnU8h)

15. [Zhang, C., Sayyari, E. & Mirarab, S. ASTRAL-III: increased scalability and impacts of contracting low support branches. in *Comparative Genomics. RECOMB-CG 2017. Lecture Notes in Computer Science* (eds. Meidanis, J. & Nakhleh, L.) 53–75 (Springer International Publishing, 2017).](http://paperpile.com/b/QHZ1Z3/wozwn)

16. [Anisimova, M. & Gascuel, O. Approximate likelihood-ratio test for branches: A fast, accurate, and powerful alternative. *Syst. Biol.* **55**, 539–552 (2006).](http://paperpile.com/b/QHZ1Z3/aD5yf)

17. [Junier, T. & Zdobnov, E. M. The Newick utilities: high-throughput phylogenetic tree processing in the UNIX shell. *Bioinformatics* **26**, 1669–1670 (2010).](http://paperpile.com/b/QHZ1Z3/ESscM)

18. [Sayyari, E. & Mirarab, S. Fast coalescent-based computation of local branch support from quartet frequencies. *Mol. Biol. Evol.* **33**, 1654–1668 (2016).](http://paperpile.com/b/QHZ1Z3/3dpNt)

19. [Sayyari, E. & Mirarab, S. Testing for polytomies in phylogenetic species trees using quartet frequencies. *Genes* **9**, (2018).](http://paperpile.com/b/QHZ1Z3/QmVia)

20. [Sayyari, E., Whitfield, J. B. & Mirarab, S. DiscoVista: interpretable visualizations of gene tree discordance. *Mol. Phylogenet. Evol.* **122**, 110–115 (2018).](http://paperpile.com/b/QHZ1Z3/QOWlO)

21. [Pattengale, N. D., Alipour, M., Bininda-Emonds, O. R. P., Moret, B. M. E. & Stamatakis, A. How many bootstrap replicates are necessary? *J. Comput. Biol.* **17**, 337–354 (2010).](http://paperpile.com/b/QHZ1Z3/9tS3W)

22. [Claramunt, S. & Cracraft, J. A new time tree reveals Earth history’s imprint on the evolution of modern birds. *Sci Adv* **1**, e1501005 (2015).](http://paperpile.com/b/QHZ1Z3/jBoVw)

23. [Claramunt, S. CladeDate: calibration information generator for divergence time estimation. *Methods Ecol. Evol.* **13**, 2331–2338 (2022).](http://paperpile.com/b/QHZ1Z3/P3XpQ)

24. [Parham, J. F. *et al.* Best practices for justifying fossil calibrations. *Syst. Biol.* **61**, 346–359 (2012).](http://paperpile.com/b/QHZ1Z3/SXwH6)

25. [Strauss, D. & Sadler, P. M. Classical confidence intervals and Bayesian probability estimates for ends of local taxon ranges. *Math. Geol.* **21**, 411–427 (1989).](http://paperpile.com/b/QHZ1Z3/BigLV)

26. [Minh, B. Q. *et al.* IQ-TREE 2: new models and efficient methods for phylogenetic inference in the genomic era. *Mol. Biol. Evol.* **37**, 1530–1534 (2020).](http://paperpile.com/b/QHZ1Z3/d02Dh)

27. [Mai, U., Sayyari, E. & Mirarab, S. Minimum variance rooting of phylogenetic trees and implications for species tree reconstruction. *PLoS One* **12**, e0182238 (2017).](http://paperpile.com/b/QHZ1Z3/CKoIP)

28. [Álvarez-Carretero, S. *et al.* A species-level timeline of mammal evolution integrating phylogenomic data. *Nature* **602**, 263–267 (2022).](http://paperpile.com/b/QHZ1Z3/OkabW)

29. [Azzalini, A. A. *The R package sn: The skew-normal and related distributions such as the skew-t and the SUN (version 2.1.1)*. (2019).](http://paperpile.com/b/QHZ1Z3/5JQEV)

30. [Yang, Z. PAML 4: phylogenetic analysis by maximum likelihood. *Mol. Biol. Evol.* **24**, 1586–1591 (2007).](http://paperpile.com/b/QHZ1Z3/UpIkN)

31. [Thorne, J. L., Kishino, H. & Painter, I. S. Estimating the rate of evolution of the rate of molecular evolution. *Mol. Biol. Evol.* **15**, 1647–1657 (1998).](http://paperpile.com/b/QHZ1Z3/ljAGL)

32. [Slack, K. E. *et al.* Early penguin fossils, plus mitochondrial genomes, calibrate avian evolution. *Mol. Biol. Evol.* **23**, 1144–1155 (2006).](http://paperpile.com/b/QHZ1Z3/AU6CK)

33. [Yang, Z. & Rannala, B. Bayesian estimation of species divergence times under a molecular clock using multiple fossil calibrations with soft bounds. *Mol. Biol. Evol.* **23**, 212–226 (2006).](http://paperpile.com/b/QHZ1Z3/msMrk)

34. [Dos Reis, M., Zhu, T. & Yang, Z. The impact of the rate prior on Bayesian estimation of divergence times with multiple loci. *Syst. Biol.* **63**, 555–565 (2014).](http://paperpile.com/b/QHZ1Z3/LLzP0)

35. [Bogdanowicz, D., Giaro, K. & Wróbel, B. TreeCmp: comparison of trees in polynomial time. *Evol. Bioinform. Online* **8**, EBO.S9657 (2012).](http://paperpile.com/b/QHZ1Z3/xM0Qq)

36. [Shen, W., Le, S., Li, Y. & Hu, F. SeqKit: a cross-platform and ultrafast toolkit for FASTA/Q file manipulation. *PLoS One* **11**, e0163962 (2016).](http://paperpile.com/b/QHZ1Z3/wXPeC)

37. [Haag, J., Höhler, D., Bettisworth, B. & Stamatakis, A. From easy to hopeless-predicting the difficulty of phylogenetic analyses. *Mol. Biol. Evol.* **39**, (2022).](http://paperpile.com/b/QHZ1Z3/XrScC)

38. [International Chicken Genome Sequencing Consortium. Sequence and comparative analysis of the chicken genome provide unique perspectives on vertebrate evolution. *Nature* **432**, 695–716 (2004).](http://paperpile.com/b/QHZ1Z3/L2CCT)

39. [Elferink, M. G., van As, P., Veenendaal, T., Crooijmans, R. P. M. A. & Groenen, M. A. M. Regional differences in recombination hotspots between two chicken populations. *BMC Genet.* **11**, 11 (2010).](http://paperpile.com/b/QHZ1Z3/suMAZ)

40. [Foster, P. G. Modeling compositional heterogeneity. *Syst. Biol.* **53**, 485–495 (2004).](http://paperpile.com/b/QHZ1Z3/xQQ6C)

41. [Duchêne, D. A., Duchêne, S. & Ho, S. Y. W. New Statistical Criteria Detect Phylogenetic Bias Caused by Compositional Heterogeneity. *Mol. Biol. Evol.* **34**, 1529–1534 (2017).](http://paperpile.com/b/QHZ1Z3/WyuMd)

42. [Duchêne, D. A., Mather, N., Van Der Wal, C. & Ho, S. Y. W. Excluding Loci With Substitution Saturation Improves Inferences From Phylogenomic Data. *Syst. Biol.* **71**, 676–689 (2022).](http://paperpile.com/b/QHZ1Z3/GMWlq)

43. [Rivas-González, I. *et al.* Pervasive incomplete lineage sorting illuminates speciation and selection in primates. *Science* **in review**,.](http://paperpile.com/b/QHZ1Z3/1yPpC)

44. [Mendes, F. K. & Hahn, M. W. Gene tree discordance causes apparent substitution rate variation. *Syst. Biol.* **65**, 711–721 (2016).](http://paperpile.com/b/QHZ1Z3/zLQ1U)

45. [Walker, J. F., Smith, S. A., Hodel, R. G. J. & Moyroud, E. Concordance-based approaches for the inference of relationships and molecular rates with phylogenomic data sets. *Syst. Biol.* **71**, 943–958 (2022).](http://paperpile.com/b/QHZ1Z3/moKex)

46. [Pagel, M. Inferring the historical patterns of biological evolution. *Nature* **401**, 877–884 (1999).](http://paperpile.com/b/QHZ1Z3/PFisH)

47. [Prum, R. O. *et al.* A comprehensive phylogeny of birds (Aves) using targeted next-generation DNA sequencing. *Nature* **526**, 569–573 (2015).](http://paperpile.com/b/QHZ1Z3/i3xhR)

48. [Kuhl, H. *et al.* An unbiased molecular approach using 3’-UTRs resolves the avian family-level Tree of Life. *Mol. Biol. Evol.* **38**, 108–127 (2021).](http://paperpile.com/b/QHZ1Z3/ofM7l)

49. [Tobias, J. A. *et al.* AVONET: morphological, ecological and geographical data for all birds. *Ecol. Lett.* **25**, 581–597 (2022).](http://paperpile.com/b/QHZ1Z3/oCCRP)

50. [Ho, L. si T. & Ané, C. A linear-time algorithm for Gaussian and non-Gaussian trait evolution models. *Syst. Biol.* **63**, 397–408 (2014).](http://paperpile.com/b/QHZ1Z3/q80ox)

51. [Revell, L. J. phytools: an R package for phylogenetic comparative biology (and other things). *Methods Ecol. Evol.* **3**, 217–223 (2012).](http://paperpile.com/b/QHZ1Z3/7v8KQ)

52. [Székely, T. *et al.* Sex roles in birds: influence of climate, life histories and social environment. (2022) doi:](http://paperpile.com/b/QHZ1Z3/udxrK)[10.5061/dryad.fbg79cnw7](http://dx.doi.org/10.5061/dryad.fbg79cnw7)[.](http://paperpile.com/b/QHZ1Z3/udxrK)

53. [Iwaniuk, A. N. & Nelson, J. E. Can endocranial volume be used as an estimate of brain size in birds? *Can. J. Zool.* **80**, 16–23 (2002).](http://paperpile.com/b/QHZ1Z3/IoEg1)

54. [Stekhoven, D. J. & Bühlmann, P. MissForest--non-parametric missing value imputation for mixed-type data. *Bioinformatics* **28**, 112–118 (2012).](http://paperpile.com/b/QHZ1Z3/TMdYO)

55. [Smaers, J. B., Mongle, C. S. & Kandler, A. A multiple variance Brownian motion framework for estimating variable rates and inferring ancestral states. *Biol. J. Linn. Soc. Lond.* **118**, 78–94 (2016).](http://paperpile.com/b/QHZ1Z3/ATlXJ)

56. [Pagel, M., Meade, A. & Barker, D. Bayesian estimation of ancestral character states on phylogenies. *Syst. Biol.* **53**, 673–684 (2004).](http://paperpile.com/b/QHZ1Z3/D3ZGp)

57. [Plummer, M., Best, N., Cowles, K., Vines, K. & Others. CODA: convergence diagnosis and output analysis for MCMC. *R news* **6**, 7–11 (2006).](http://paperpile.com/b/QHZ1Z3/KtezS)

58. [Pennell, M. W. *et al.* geiger v2.0: an expanded suite of methods for fitting macroevolutionary models to phylogenetic trees. *Bioinformatics* **30**, 2216–2218 (2014).](http://paperpile.com/b/QHZ1Z3/Qau5T)

59. [Cooney, C. R. *et al.* Mega-evolutionary dynamics of the adaptive radiation of birds. *Nature* **542**, 344–347 (2017).](http://paperpile.com/b/QHZ1Z3/hYkUY)

60. [Oliveros, C. H. *et al.* Earth history and the passerine superradiation. *Proc. Natl. Acad. Sci. U. S. A.* **116**, 7916–7925 (2019).](http://paperpile.com/b/QHZ1Z3/5Hs5O)

61. [Jønsson, K. A. *et al.* A supermatrix phylogeny of corvoid passerine birds (Aves: Corvides). *Mol. Phylogenet. Evol.* **94**, 87–94 (2016).](http://paperpile.com/b/QHZ1Z3/8BGye)

62. [Moyle, R. G. *et al.* Tectonic collision and uplift of Wallacea triggered the global songbird radiation. *Nat. Commun.* **7**, 12709 (2016).](http://paperpile.com/b/QHZ1Z3/grD26)
