# Supplementary material for: Complexity of avian evolution revealed by family-level genomes
Source: Nature. 2024 Apr 1;629(8013):851–60. doi: 10.1038/s41586-024-07323-1 (PMC11111414; doi:10.1038/s41586-024-07323-1)
Supplement: Supplementary file 2 — Reporting Summary [file 41586_2024_7323_MOESM2_ESM.pdf]

nature portfolio

Corresponding author(s): Stiller, Mirarab, Zhang

Last updated by author(s): Mar 6, 2024

Reporting Summary

Nature Portfolio wishes to improve the reproducibility of the work that we publish. This form provides structure for consistency and transparency in reporting. For further information on Nature Portfolio policies, see our Editorial Policies and the Editorial Policy Checklist.

Statistics

For all statistical analyses, confirm that the following items are present in the figure legend, table legend, main text, or Methods section.

n/a	Confirmed	
☐	☒	The exact sample size (*n*) for each experimental group/condition, given as a discrete number and unit of measurement
☐	☒	A statement on whether measurements were taken from distinct samples or whether the same sample was measured repeatedly
☐	☒	The statistical test(s) used AND whether they are one- or two-sided<br>*Only common tests should be described solely by name; describe more complex techniques in the Methods section.*
☒	☐	A description of all covariates tested
☐	☒	A description of any assumptions or corrections, such as tests of normality and adjustment for multiple comparisons
☐	☒	A full description of the statistical parameters including central tendency (e.g. means) or other basic estimates (e.g. regression coefficient) AND variation (e.g. standard deviation) or associated estimates of uncertainty (e.g. confidence intervals)
☐	☒	For null hypothesis testing, the test statistic (e.g. *F*, *t*, *r*) with confidence intervals, effect sizes, degrees of freedom and *P* value noted<br>*Give P values as exact values whenever suitable.*
☐	☒	For Bayesian analysis, information on the choice of priors and Markov chain Monte Carlo settings
☒	☐	For hierarchical and complex designs, identification of the appropriate level for tests and full reporting of outcomes
☒	☐	Estimates of effect sizes (e.g. Cohen's *d*, Pearson's *r*), indicating how they were calculated

Our web collection on statistics for biologists contains articles on many of the points above.

Software and code

Policy information about availability of computer code

Data collection	All open source code and custom code used to collect the data is described in detail with versions in the methods section. Specifically, we used https://github.com/Secretloong/Cactus_Alignments_Tools, https://github.com/uym2/TreeShrink/tree/master/related_scripts, HAL v.2.3, PASTA v.1.8.5, TreeShrink v.1.3.1, MAFFT v7.149b, PHYLUCE v.1.6.3, Pargenes v.1.1.0, Modeltest-NG v.0.1.3, RAXML-NG v.0.9.0, RAXML-NG v.1.0.1, IQTREE v.1.6.12, IQTREE v2.0.4, newick-utilities v.1.6, ASTRAL-III v.5.14.5, FastRoot, CladeDate, MCMCtree v.4.9h, TreeCmp v.2.0, seqkit v.2.2.0, Pythia v. 1.0.0, PhyloMAd, CoalHMM, BayesTraits v.4, . We used the following R packages and functions: sn::st.mple v.2.0.0, phylolm, fastBM, evomap, missForest.
Data analysis	All open source code and custom code used to analyze the data is described in detail with versions in the methods section. Specifically, we used DiscoVista and functions implemented in base R for statistical analysis. Plotting for figures was done in R with dependencies contained in the scripts deposited in the data repository at https://doi.org/10.17894/ucph.85624f66-c8e5-4b89-8e8a-fe984ca89e4a

For manuscripts utilizing custom algorithms or software that are central to the research but not yet described in published literature, software must be made available to editors and reviewers. We strongly encourage code deposition in a community repository (e.g. GitHub). See the Nature Portfolio guidelines for submitting code & software for further information.

Data

Policy information about availability of data

All manuscripts must include a data availability statement. This statement should provide the following information, where applicable:

- Accession codes, unique identifiers, or web links for publicly available datasets
- A description of any restrictions on data availability
- For clinical datasets or third party data, please ensure that the statement adheres to our policy

The genome assemblies analyzed in this study and their whole genome alignment were part of the study by Feng et al. Nature 2020 and accession numbers are given as part of the Supplementary Data. Alignments, gene trees and species trees, in addition to data files produced for their analysis and scripts for plotting figures are available at https://doi.org/10.17894/ucph.85624f66-c8e5-4b89-8e8a-fe984ca89e4a. This repository also contains a file detailing contents and commands to use for individual and batch download of files. The study analyzed morphological trait data from AVONET (https://figshare.com/s/b990722d72a26b5bfead) and from https://doi.org/10.5061/dryad.fbg79cnw7, recombination rates for chicken (https://static-content.springer.com/esm/art%3A10.1186%2F1471-2156-11-11/MediaObjects/12863_2009_758_MOESM5_ESM.XLS), and time-calibrated species trees from Jarvis et al. Science 2014 (http://gigadb.org/dataset/101041) and Prum et al. Nature 2015 (Avian-TimeTree.tre from https://zenodo.org/records/28343).

Research involving human participants, their data, or biological material

Policy information about studies with human participants or human data. See also policy information about sex, gender (identity/presentation), and sexual orientation and race, ethnicity and racism.

Reporting on sex and gender	The study does not involve human participants or human data.
Reporting on race, ethnicity, or other socially relevant groupings	The study does not involve human participants or human data.
Population characteristics	The study does not involve human participants or human data.
Recruitment	The study does not involve human participants or human data.
Ethics oversight	The study does not involve human participants or human data.

Note that full information on the approval of the study protocol must also be provided in the manuscript.

Field-specific reporting

Please select the one below that is the best fit for your research. If you are not sure, read the appropriate sections before making your selection.

☐ Life sciences ☐ Behavioural & social sciences ☒ Ecological, evolutionary & environmental sciences

For a reference copy of the document with all sections, see nature.com/documents/nr-reporting-summary-flat.pdf

Ecological, evolutionary & environmental sciences study design

All studies must disclose on these points even when the disclosure is negative.

Study description	The study investigates phylogenetic relationships among bird species using whole genome sequences, spanning 363 species of birds.
Research sample	The loci for phylogenetic analysis were extracted from an existing whole genome alignment (https://doi.org/10.1038/s41586-020-2873-9) and analyzed using phylogenetic methods.
Sampling strategy	Sampling targeted at least one member for each taxonomic family of extant birds.
Data collection	We collected 159205 genetic loci from the whole genome alignment across the bird species using bioinformatic methods. For each locus we built a gene tree, which were summarized into species trees.
Timing and spatial scale	The data were extracted from the whole genome alignment at a single time point.
Data exclusions	We only excluded minimal amounts of data. We excluded fragmentary sequences, i.e. sequences shorter than 50% of the median length of all sequences of the locus because these fragmentary sequences can impact alignment accuracy and contain fewer parsimony informative sites than the remaining sequences. Secondly, we removed loci with fewer than 4 sequences as this is the minimum number of sequences needed to construct a tree.
Reproducibility	We performed bootstrapping to estimate statistical support on nodes of the best estimated tree. For subsetting analyses sampling a certain fraction of all gene trees, we performed 50 replicates to estimate amount of variation in the replicates.

Randomization	Decisions on groupings were based on bioinformatic cutoffs, therefore randomization was not relevant.
Blinding	Decisions on groupings were based on bioinformatic cutoffs, therefore blinding was not relevant.

Did the study involve field work? ☐ Yes ☒ No

Reporting for specific materials, systems and methods

We require information from authors about some types of materials, experimental systems and methods used in many studies. Here, indicate whether each material, system or method listed is relevant to your study. If you are not sure if a list item applies to your research, read the appropriate section before selecting a response.

Materials & experimental systems

n/a	Involved in the study	
☒	☐	Antibodies
☒	☐	Eukaryotic cell lines
☒	☐	Palaeontology and archaeology
☒	☐	Animals and other organisms
☒	☐	Clinical data
☒	☐	Dual use research of concern
☒	☐	Plants

Methods

n/a	Involved in the study	
☒	☐	ChIP-seq
☒	☐	Flow cytometry
☒	☐	MRI-based neuroimaging

Plants

Seed stocks	Not applicable.
Novel plant genotypes	Not applicable.
Authentication	Not applicable.
